# Programmable direct-patterning assembly enables high-density and surface-conformal integration of fiber Bragg grating sensor arrays

**DOI:** 10.1038/s41467-026-72613-3

**Published:** 2026-05-04

**Authors:** Yin Tao, Wen Xu, Peishi Yu, Aoqi Shen, Yuxiang Zhao, Xin Zhang, Maoyang Li, Junhua Zhao

**Affiliations:** 1 Jiangsu Key Laboratory of Advanced Food Manufacturing Equipment and Technology, School of Mechanical Engineering, Jiangnan University, Wuxi, PR China; 2 Jiangsu Province Engineering Research Center of Micro-Nano Additive and Subtractive Manufacturing, Institute of Advanced Technology, Jiangnan University, Wuxi, PR China

**Keywords:** Mechanical engineering, Mathematics and computing

## Abstract

Fiber Bragg grating (FBG) sensors are widely used in aerospace monitoring and intelligent manufacturing due to their high sensitivity, yet their deployment relies on manual assembly, limiting precision, integration density, and conformality under extreme conditions. Here, we propose a programmable direct-FBG-patterning (DFP) assembly paradigm for one-step integration of intrinsically brittle multiplexed FBG arrays onto arbitrary curved and non-developable surfaces. A mechanics-optics coupled framework identifies three minimum bending radii governed by interfacial debonding, fiber fracture, and optical attenuation, defining the achievable feature size and multidirectional sensing capability. Guided by this framework, cross-fiber routing and conformal assembly strategies enable high-density sensor networks along a single continuous fiber beyond the limits of manual assembly. We further demonstrate robust and versatile FBG sensor integration in applications including structural displacement reconstruction, phonation, and gesture monitoring, establishing a general strategy for quasi-distributed sensing beyond the constraints of manual assembly.

## Abstract (web summary)

This study presents an automated paradigm for assembling high-density fiber Bragg sensor arrays on complex surfaces. The framework ensures signal fidelity and structural integrity, enabling precise real-time monitoring of complex deformations.

## Introduction

In recent years, commercial fiber Bragg grating (FBG) sensors have become indispensable tools in intelligent systems^1,2^, energy storage systems^3–5^, structural health monitoring (SHM)^6,7^, and biomedical engineering^8–10^, owing to their exceptional sensitivity, immunity to electromagnetic interference, and rapid response. These intrinsic advantages make FBG sensors superior to conventional electronic and most flexible sensors, particularly in harsh or safety-critical environments^11,12^. However, the transition of FBG arrays from discrete laboratory components to high-density, system-level sensing networks remains fundamentally constrained by conventional manual assembly methodologies^13–15^. Traditional deployment primarily relies on manual adhesive bonding, which inherently restricts sensing to basic longitudinal or transverse patterns^16–19^. These labor-intensive processes suffer from poor geometric repeatability, limited integration density, and, most critically, an inability to conformally adhere to the complex, non-developable surfaces typical of advanced engineering structures.

The primary scientific challenge in automating FBG deployment lies in the extreme brittleness of silica-based monofilaments, which are prone to mechanical fracture or interfacial debonding when subjected to the dynamic stresses of high-curvature routing^20,21^. Furthermore, macroscopic bending beyond a certain threshold induces significant optical power attenuation, which can compromise the signal integrity of downstream multiplexed gratings^22,23^. While optical fiber (OF) inscription technologies (e.g., femtosecond laser or phase-mask-based writing) have matured significantly^24–26^, a generalizable assembly paradigm that reconciles programmable, one-step assembly with these coupled mechanical and optical constraints has yet to be established.

In this work, we propose a programmable direct-FBG-patterning (DFP) assembly paradigm for the high-precision and one-step conformal integration of pre-inscribed FBGs onto arbitrary curved surfaces. Distinct from optical grating inscription, the proposed DFP approach focuses on the deterministic mechanical assembly and routing of OFs, enabling complex sensing layouts that are inaccessible to conventional manual bonding or planar lamination. Specifically, DFP employs a multi-degree-of-freedom motion platform, in which the fiber is continuously guided along predefined trajectories, analogous to path-defined material deposition in direct ink writing (DIW), but replacing extruded ink with solid-state fibers. Central to this assembly paradigm is a mechanics-optics coupled design framework constituted by three critical minimum bending radii, which collectively define the feasible assembly window for brittle OFs. These radii are derived by jointly considering interfacial adhesion, fiber fracture, and bending-induced optical attenuation, thereby establishing quantitative constraints on routing curvature and minimum feature size. Building upon these constraints, DFP enables the conformal assembly of high-density, single-fiber multiplexed FBG arrays on complex curved surfaces with exceptional angular precision and spatial fidelity. This methodology effectively overcomes the inherent limitations in precision, scalability, and integration density that characterize traditional manual deployment. Leveraging this capability, we have successfully realized large-area and high-precision sensing arrays for dynamic response applications, including torsional displacement reconstruction in wing-like structures as well as human phonation and gesture monitoring. Consequently, this study not only enhances the monitoring accuracy of quasi-distributed sensor networks but also advances the assembly of FBG sensors from conventional SHM toward more complex and intelligent dynamic applications.

## Results

### Programmable DFP assembly paradigm and multi-radius constraint framework

**Fig. 1:**
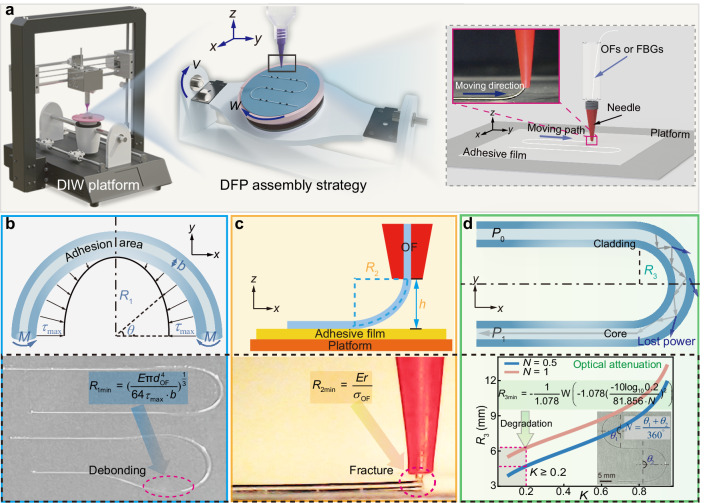
Programmable direct-FBG-patterning (DFP) strategy and multi-radius constraint framework. **a** Schematic illustration of the automated DFP assembly system. A custom-built five-axis platform (*x*, *y*, *z* three translational degrees of freedom on the upper print needle and *xoy* (*W*), *xoz* (*V*) two rotational degrees of freedom on the lower substrate), inspired by direct ink writing (DIW), enables programmable and high-precision path-defined assembly of multiplexed fiber Bragg grating (FBG) arrays or bare optical fibers (OFs) onto planar and curved substrates. **b** Interfacial stability model (*R*_1min_) to prevent OF debonding in the *x*-*y* plane. The continuum modeling accounts for the balance between the fiber’s intrinsic bending moment and the interfacial shear stress (*τ*_max_). The experimental image shows interfacial debonding when the bending radius falls below the theoretical limit. **c** Mechanical integrity model (*R*_2min_) to prevent brittle failure in the *x*-*z* plane during the assembly process. The bending radius *R*_2_ is governed by the relative height (*h*) between the deposition needle and the substrate. Reducing *h* below the critical threshold leads to brittle fracture of the OF, as shown in the optical micrograph. **d** Functional reliability model (*R*_3min_) based on macrobending-induced optical power attenuation. The schematic shows the power loss mechanism as light propagates through a curved waveguide. The corresponding graph identifies the functional patterning window where the output-to-input power ratio (*K* = *P*_1_/*P*_0_) remains ≥ 0.2, ensuring signal integrity for downstream multiplexed gratings. Blue and red lines represent the bending angle proportions *N* = 0.5 and *N* = 1, respectively, where *N* = *θ*/360° represents the proportion of the total bending angle of the OF relative to the entire circumference.

As schematically illustrated in Fig. 1a, a multi-degree-of-freedom motion platform continuously guides OFs along predefined paths, enabling deterministic assembly on both planar and curved substrates. Unlike conventional manual bonding, the DFP assembly paradigm relies on controlled interfacial traction between the fiber and the adhesive film to extract, steer, and secure the fiber along the programmed trajectory in a single automated step. Notably, the achievable routing curvature and feature size are intrinsically limited by the coupled mechanical response of the fiber-substrate system and the optical tolerance of the guided fiber, motivating a quantitative analysis of the underlying constraints.

The geometric resolution and functional reliability of integrated multiplexed FBG arrays are fundamentally governed by coupled mechanical and optical constraints arising during the assembly process. To elucidate these limits, we established a mechanics-optics coupled framework that identifies three distinct failure modes, which together define the feasible bending window of a single OF, as summarized in Fig. 1b–d. The first constraint corresponds to interfacial debonding between the OF and the adhesive film (Fig. 1b). When the in-plane bending radius becomes sufficiently small, interfacial shear stresses exceed the adhesive strength, resulting in local detachment and loss of pattern fidelity. This failure mode defines a minimum in-plane bending radius, denoted as *R*_1min_, which depends on the elastic properties of the fiber and the interfacial adhesion characteristics of the substrate. The corresponding continuum modeling is described by Eq. (1):

1 $${R}_{1\,\min }={(\frac{E{{{\rm{\pi }}}}{d}_{{{\rm{OF}}}}^{4}}{64{\tau }_{\max }\cdot b})}^{\frac{1}{3}}$$

where *E* and *d*_OF_ represent the Young’s modulus and diameter of the OF material, respectively, while* τ*_max_ and *b* denote the maximum adhesive strength at the OF/adhesive film interface and the contact width, respectively.

The second constraint arises from mechanical fracture of the OF during out-of-plane bending in the assembly process (Fig. 1c). As the OF is guided from the deposition needle onto the adhesive substrate, excessive curvature induces stresses that may exceed the intrinsic bending strength of the fiber, leading to brittle failure. This fracture-dominated limit defines a second minimum bending radius, *R*_2min_, which governs process stability and continuous routing of multiplexed arrays, as described in Eq. (2):

2 $${R}_{2\,\min }=\frac{Er}{{\sigma }_{{\mbox{OF}}}}$$

where *r* and *σ*_OF_ represent the radius and intrinsic bending strength of the OF, respectively.

Beyond purely mechanical considerations, macroscopic bending of the OF also induces functional degradation through optical power attenuation (Fig. 1d). Excessive curvature causes significant transmission loss, impairing the ability of downstream multiplexed gratings to operate reliably. By defining an optical failure criterion based on a conservative and application-relevant power attenuation ratio (*K* = *P*_1_/*P*_0_ ≥ 0.2), an optical functional integrity limiting radius (*R*_3min_) is established, as expressed in Eq. (3):

3 $${R}_{3\,\min }=-\frac{1}{1.078}{{{\rm{W}}}}\left(-1.078{\left(\frac{-10{\log }_{10}0.2}{81.856\cdot N}\right)}^{2}\right)$$

where *N* represents the proportion of the total bending angle of the OF relative to the entire circumference, and W represents the Lambert W function.

Together, this coupled mechanics-optics framework defines the achievable feature size, routing density, and functional integrity of quasi-distributed DFP-assembled FBG arrays. To validate its predictive capability, experimental measurements were conducted on several adhesive substrates, including optically clear adhesive (OCA), nanotape, and double-sided tape. The experimentally determined critical minimum bending radii are summarized in Table 1. The close agreement between experimental measurements and theoretical predictions confirms the robustness of the proposed framework. Detailed analytical derivations and experimental procedures are provided in Supplementary Notes 1, 2, Supplementary Figs. 1–6 and Supplementary Table 1. These thresholds establish practical design constraints for the automated DFP assembly process, ensuring structural stability, mechanical integrity, and signal fidelity.

**Table 1:** The minimum radius values on different adhesive films

Adhesive film materials	*R*_1min-e_ / *R*_1min-t_ (mm)	*R*_2min-e_ / *R*_2min-t_ (mm)	*R*_3min-t_ (mm)
OCA	5 / 4.46	1.59 / 1.64	4.7 (*N* = 0.5) 6.3 (*N* = 1)
Nanotape	2.2 / 2.25		
Double-sided tape	3.5 / 3.23		

Note: *R*_1min-e_, *R*_2min-e_ and *R*_2min-e_, *R*_2min-t_ represent the experimental and theoretical minimum radii for interfacial stability and mechanical integrity, respectively. The values of *R*_2min_ and *R*_3min-t_ are determined by the mechanical limits of the optical fiber and optical attenuation constraints, which are independent of the adhesive materials. *N* represents the proportion of the total bending angle of the optical fiber relative to the entire circumference. OCA, optically clear adhesive.

### Geometric patterning capability and functional FBG integration enabled by DFP

**Fig. 2:**
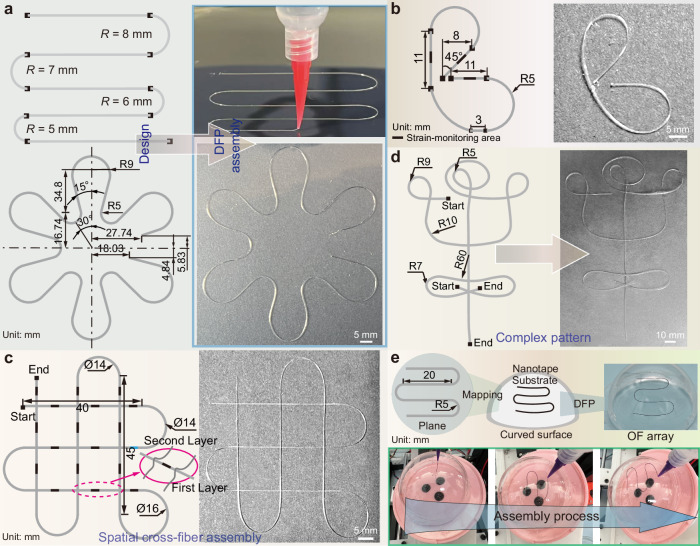
Geometric patterning capability and functional fiber Bragg grating (FBG) integration enabled by direct-FBG-patterning (DFP). **a** Representative planar pattern with continuously varying curvature and sharp turns, demonstrating high-precision assembly layout over large areas. The black squares represent the printing path. **b** Design and assembly of an orientation-defined triaxial strain-rosette, where the spatial layout of optical fiber (OF) segments encodes directional sensing axes. The black line segments schematically indicate the pre-defined locations of integrated FBG sensors for intended strain monitoring. **c** Schematic and optical micrograph of the multilayer fiber stacking process, achieved by precise vertical clearance control of the fiber-guiding needle. **d** Demonstration of a complex pattern composed of intricate arcs, straight segments, and spline curves, highlighting the versatility of DFP for customized routing. **e** Continuous OF array assembled on a spherical shell substrate, validating the interfacial stability and conformity of the patterning process on 3D geometries.

Guided by the established bending constraints, the DFP process facilitates the deterministic patterning of OFs into geometries with spatial densities and routing complexities that surpass the limitations of conventional manual assembly. To systematically evaluate the geometric versatility of the process while decoupling it from grating-related fragility, standard single-mode fibers were first utilized as model filaments. Representative single-layer patterns featuring continuously varying curvature, sharp turns, and complex routing are shown in Fig. 2a, demonstrating stable adhesion and accurate realization of digitally programmed routing paths over large substrate areas. These results underscore the fundamental geometric fidelity and operational robustness of DFP for assembling delicate fiber-based elements. Beyond purely geometric demonstrations, DFP further enables the direct construction of orientation-defined sensing structures through programmable fiber routing. As shown in Fig. 2b, a triaxial strain-rosette layout is assembled by precisely assembling a single OF at predefined angular orientations within a localized domain. While bare fibers were employed here, the spatial arrangement serves as a functional template that intrinsically encodes directional sensing capabilities. Consequently, the DFP process directly translates digital sensing motifs into physical architectures, requiring only the subsequent incorporation of FBGs to activate full multi-axial strain-sensing functionality. This approach eliminates the need for manual alignment or post-assembly adjustment, enabling the scalable and reproducible construction of complex, fiber-integrated sensor systems. To further increase integration density, the DFP process was extended to enable spatial cross-fiber and multilayer assembly. By precisely controlling the relative height and orientation between the fiber-guiding needle and the substrate, fibers were routed across pre-bonded paths without inducing fracture or interfacial delamination (Fig. 2c). Precise control of vertical clearance is critical, as overlap at fiber crossings must be managed to maintain adhesion stability. Balancing these constraints allowed the successful assembly of complex multilayer patterns composed of arcs and spline curves, illustrating the versatility of DFP for customized, high-density fiber patterns (Fig. 2d).

Beyond planar substrates, DFP enables conformal patterning directly onto non-developable curved surfaces. Initial validation was performed using continuous OF assembled on a spherical shell (Fig. 2e), where the high compliance of the adhesive layer accommodated local curvature without inducing OF debonding. The five-axis motion platform accurately mapped the digitally defined routing paths onto the curved geometry, maintaining uniform in-plane spacing across the three-dimensional surface. These results demonstrate the capability of DFP to preserve geometric fidelity during surface-conformal assembly. The transition from bare OF to FBG assembly introduces additional manufacturing constraints due to the increased brittleness of grating-inscribed regions. By leveraging the five-axis DFP platform to dynamically regulate nozzle orientation and minimize local stress concentrations, a functional FBG strain rosette was successfully conformally assembled on a spherical substrate, while the strategy’s robust manufacturability and interfacial stability were further validated across a diverse range of planar and curved substrates with distinct properties (Supplementary Note 3, Supplementary Figs. 7–9). The successful realization of these functional layouts confirms that DFP enables the direct translation of digitally designed sensing architectures onto complex three-dimensional structures without manual positioning or post-assembly correction.

### System-level demonstrations enabled by DFP-assembled FBG arrays

To evaluate the practical reliability and functional integrity of FBG arrays integrated via the DFP process, we conducted a series of system-level demonstrations under diverse mechanical conditions. These experiments were designed not only to illustrate potential applications but, more importantly, to verify that the automated DFP assembly does not damage or degrade the optical and mechanical performance of the FBG sensors. While the use of FBGs for mechanical vibration, speech recognition, and hand gesture monitoring has been previously established^27–29^, these demonstrations are implemented here to confirm that the DFP paradigm preserves the sensors’ full functionality. After assembly, the reflected spectra of the FBG arrays were measured to confirm stable and reliable optical responses, indicating that the programmed fiber routing does not introduce significant spectral distortion or signal loss. In addition, the following demonstrations in structural health monitoring and biomechanical sensing further confirm that the interfacial adhesion between the fiber and substrate is sufficiently robust to enable accurate strain transfer and reliable motion determination under both rigid and soft mechanical loading conditions.

**Fig. 3:**
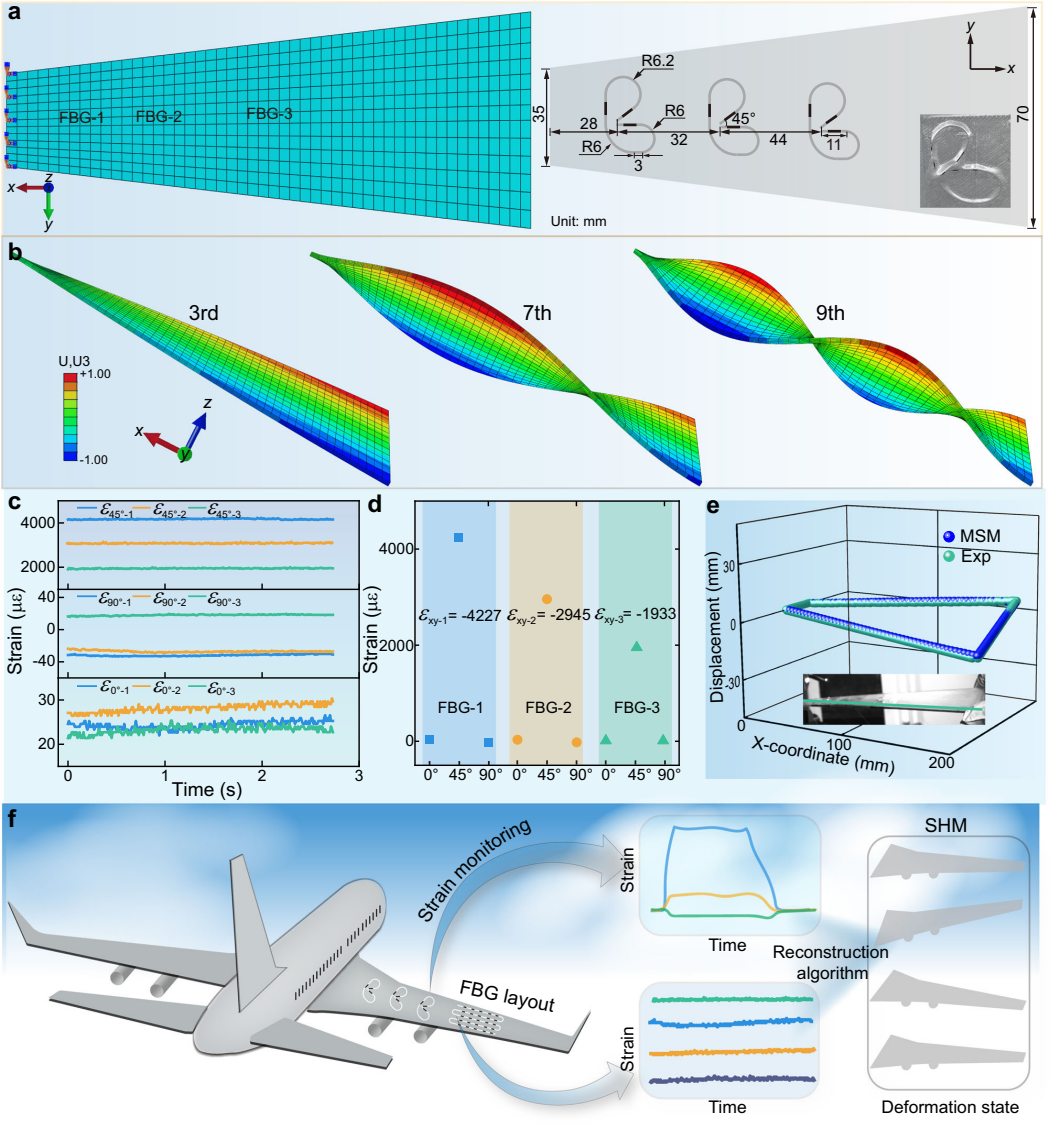
System-level structural displacement reconstruction enabled by direct-FBG-patterning (DFP)-assembled fiber Bragg grating (FBG) strain rosettes. **a** Finite element (FE) model mesh and the corresponding layout of three DFP-assembled FBG strain rosettes (FBG−1, FBG-2, and FBG-3). The specifies the geometric parameters and angular orientations of the sensing units, with an inset showing an optical micrograph of the high-fidelity fiber integrated on the structure. **b** Visualization of the third-, seventh-, and ninth-order torsion-sensitive mode shapes derived from modal analysis, which serve as the mathematical basis for the structural displacement reconstruction algorithm. The color scale represents the normalized displacement (U, U_3_) along the *z*-direction, ranging from -1 to 1. **c** Real-time multi-channel strain signals recorded by the three FBG rosettes across 0°, 45°, and 90° sensing directions during torsional loading. **d** Extracted local shear strain values (*ε*_xy-1_, *ε*_xy-2_, and *ε*_xy-3_) calculated for each sensor node based on the captured strain signatures. **e** Quantitative validation of the sensing performance. The 3D plot compares the displacement field reconstructed via the modal superposition method (MSM, blue dots) with experimental measurements (Exp, green dots), demonstrating strong spatial agreement and efficient strain transfer. **f** Conceptual framework for aerospace applications. The diagram illustrates the integrated sensing pipeline where DFP-assembled layouts enable large-area strain monitoring, algorithmic reconstruction, and real-time deformation state assessment for advanced structural health monitoring.

The functional integrity and system-level applicability of the DFP assembly strategy were rigorously validated through a representative SHM demonstration implemented on an aerospace-inspired wing component^30,31^. To optimize the monitoring efficiency, three FBG strain rosettes (FBG-1, FBG-2, and FBG-3) were precisely patterned at predefined positions using the DFP assembly process, guided by finite element analysis (FEA) to capture the complex multidirectional structural response (Fig. 3a). The modal superposition method (MSM) was adopted as the theoretical framework for displacement reconstruction, employing the third-, seventh-, and ninth-order torsion-sensitive mode shapes as the modal basis (Fig. 3b). Under unknown torsional loading, the DFP-assembled rosettes simultaneously recorded stable and high-fidelity strain signals across the 0°, 45°, and 90° sensing orientations, the results of which are presented in Fig. 3c. Upon analysis, the extracted local shear strains (*ε*_xy_) shown in Fig. 3d exhibited a distinct directional dependence across the sensing locations, revealing the specific strain distribution at each position. Leveraging these high-precision measurements, the structural displacement field was reconstructed with exceptional agreement relative to experimentally measured displacement, as evidenced by the comparison in Fig. 3e. The MSM-derived coordinates closely matched the experimental profile with minimal deviation, confirming that the DFP assembly process ensures robust strain transfer and preserves the full sensing capabilities of pre-inscribed FBGs in realistic, high-load environments. These results collectively demonstrate the scalability of the DFP strategy for deploying large-area intelligent sensing networks. By bridging the gap between automated manufacturing and advanced structural diagnostics, DFP provides a robust pathway for real-time deformation monitoring and health management in next-generation aviation systems (Fig. 3f).

**Fig. 4:**
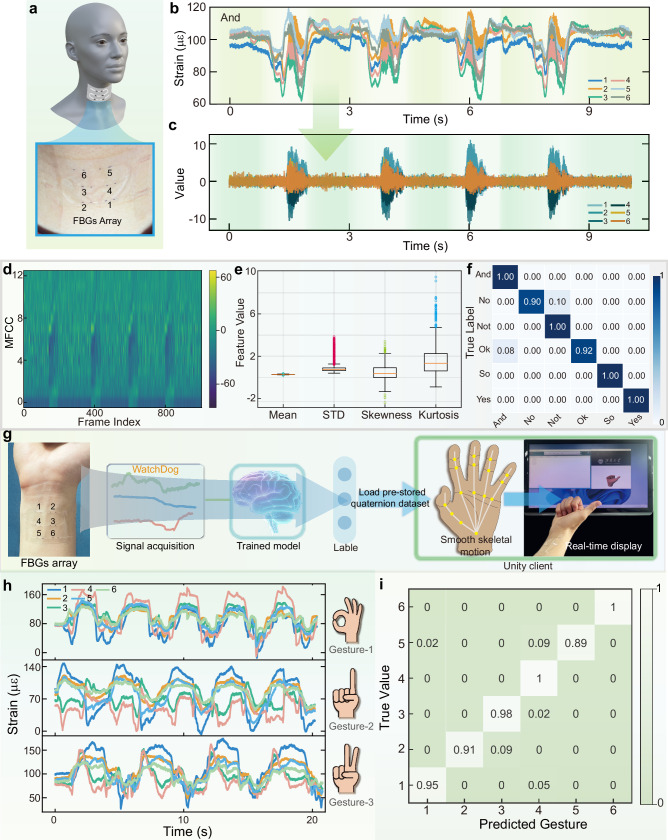
System-level demonstrations of human phonation and gesture recognition enabled by conformal fiber Bragg grating (FBG) sensor arrays. **a** Conformal assembly of a 3 × 2 FBG sensor array onto the laryngeal region for high-frequency phonation monitoring. **b** Representative multi-channel signals collected by the FBG sensor array during the vocalization of the word “And”. Colored lines (numbered 1–6) represent the six independent FBG sensing channels. **c** The extracted periodic oscillations of the word “And” from the raw data, reflecting the subtle vibrational state of the vocal cords. **d** Two-dimensional Mel-frequency cepstral coefficient (MFCC) spectral feature map derived from the temporal strain signals, highlighting acoustic periodicity and formant characteristics. The color scale (from blue to yellow) indicates the normalized power spectral density. **e** Statistical distribution of extracted features (mean, standard deviation (STD), skewness, and kurtosis) utilized for deep-learning classification. **f** Confusion matrix demonstrating a word recognition accuracy of 95% across six distinct categories using a dual-attention convolutional recurrent neural network (CRNN) model. The blue color intensity represents the classification probability from 0 to 1. **g** System frame for epidermal gesture recognition, illustrating the signal acquisition, deep-learning inference, and real-time Unity-based virtual hand animation. **h** Distinct temporal strain response signatures recorded at the volar wrist for three characteristic gestures (OK, one and two), exhibiting a large dynamic range. Colored lines correspond to the six sensing channels. **i** Confusion matrix for gesture classification, achieving a mean accuracy exceeding 90%, confirming the robustness of the direct-FBG-patterning (DFP)-assembled sensing network for complex human-machine interaction. The green color intensity represents the classification probability.

To further demonstrate the versatility and robustness of the DFP assembly strategy, its application was extended from rigid industrial structures to soft, dynamic human surfaces for intelligent activity recognition^32–36^. As shown in Fig. 4, this transition validates the capability of DFP-assembled FBG arrays to maintain stable interfacial bonding and high signal fidelity under heterogeneous biomechanical regimes. Initially, a phonation monitoring system was developed through the conformal assembly of a 3 × 2 multiplexed FBG array onto the laryngeal region (Fig. 4a). During the pronunciation of specific words, such as ‘And’, the array successfully captured distinct multi-channel microstrain signatures (Fig. 4b). Following baseline calibration and denoising, these raw strain data were transformed into clear periodic oscillation waveforms, which reflect the subtle vibrational state of the vocal cords (Fig. 4c). To facilitate deep-learning classification, we extracted Mel-frequency cepstral coefficients (MFCCs) to convert the temporal strain signals into two-dimensional spectral feature maps. Figure 4d illustrates how these maps effectively highlight acoustic periodicity and formant characteristics. Statistical analysis of the extracted features, including mean, standard deviation, skewness, and kurtosis, further confirmed the discriminative nature of the captured vibrations (Fig. 4e). By implementing a convolutional recurrent neural network (CRNN) architecture combined with a dual-attention mechanism, the system achieved a word recognition accuracy of 95% across six distinct categories, highlighting its potential for non-invasive speech assistance (Fig. 4f).

In parallel, the DFP strategy was applied to an epidermal gesture recognition system for monitoring large-amplitude kinematic strains, as illustrated in Fig. 4g. A multiplexed FBG array was assembled on the volar wrist to capture complex skin deformation patterns associated with different hand gestures. The resulting strain signals exhibited gesture-specific temporal signatures with amplitudes substantially larger than those observed during phonation, as shown in Fig. 4h, highlighting the wide dynamic sensing range enabled by DFP-assembled arrays. The robust signal-to-noise ratio and repeatable spatiotemporal signatures provided a reliable hardware foundation for high-level signal interpretation. Subsequently, a real-time processing pipeline was implemented in which strain signals acquired by an FBG interrogator were classified using a trained deep learning model, yielding a mean gesture recognition accuracy exceeding 90%, as summarized in Fig. 4i. The decoded gesture labels were further mapped to a Unity-based virtual environment to realize smooth, real-time skeletal motion visualization (Supplementary Movie 1). Together, these system-level demonstrations confirm that the DFP assembly strategy preserves the full functional integrity of multiplexed FBG sensors while enabling reliable deployment on soft, curved, and dynamically deforming surfaces. By providing a robust hardware foundation for high-level signal interpretation, DFP effectively bridges conformal sensor integration with intelligent human-machine interaction.

## Discussion

This work establishes a programmable DFP paradigm specifically engineered for the high-precision assembly of brittle FBG arrays on complex surfaces. By shifting the focus from grating inscription to an assembly-centric methodology, we address the long-standing challenge of deploying fragile, high-performance sensors in non-planar environments. Unlike conventional manual assembly, which is fundamentally limited by human error and geometric constraints, the DFP paradigm enables the deterministic placement and routing of multiplexed FBGs through digitally programmed paths. This transition from manual pasting to an automated, path-defined assembly allows for the creation of high-density, multi-directional sensing structures that were previously unattainable.

A central outcome of this study is the development of a mechanics-optics-coupled framework that defines three critical minimum bending radii: *R*_1min_ (interfacial debonding), *R*_2min_ (fiber fracture), and *R*_3min_ (bending-induced optical attenuation). These constraints collectively delineate the feasible design space for DFP-assembled FBG patterns, governing the minimum feature size and routing curvature. While the derived models treat adhesive layers as isotropic and homogeneous for theoretical clarity, we acknowledge that practical materials such as nanotape may exhibit viscoelastic behavior and local heterogeneity. Nevertheless, the strong agreement between our predictions and experimental results indicates that this framework provides a reliable design guideline for automated FBG assembly. Critically, while bending typically compromises FBG performance through optical loss, our framework transforms these physical constraints into quantifiable design advantages. By establishing the *R*_3min_ threshold, the DFP paradigm bypasses traditional trial-and-error manual methods, maximizing integration density without compromising signal fidelity. Spectral characterization confirms that the automated routing induces negligible crosstalk or wavelength drift, proving the process preserves the structural and functional integrity of brittle sensors. The reliability of this approach is evidenced by high-fidelity strain transfer in structural displacement reconstruction (Fig. 3e) and superior classification accuracies in speech (95%) and gesture (90%) recognition. Ultimately, the DFP paradigm offers a scalable, programmatic route for integrating fragile fiber electronics onto complex, non-developable surfaces, overcoming the geometric constraints of conventional assembly and paving the way for fully automated manufacturing of intelligent skins and soft robotics.

Temperature sensitivity remains an inherent consideration for the real-world deployment of FBG sensing networks. Although all validation experiments in this study were performed under controlled laboratory conditions at 26 °C, where temperature-induced wavelength drift was negligible, practical environments may require active or passive compensation strategies. Importantly, the DFP assembly paradigm is intrinsically compatible with established temperature compensation approaches, including reference gratings, differential configurations, and co-located sensing structures. The ability to precisely and programmatically position both sensing and reference FBGs within the same conformal layout provides a stable hardware foundation for reliable signal decoupling and downstream data processing. In addition to environmental temperature considerations, the long-term stability of the adhesive interface is equally crucial for practical reliability. While the current demonstrations validate the robustness of DFP-integrated sensors under immediate loading, their resistance to environmental aging and mechanical fatigue under cyclic bending remains an essential avenue for future characterization. In aerospace applications, where bonding must maintain integrity over extended service lives, systematic durability testing will be essential. Future work will focus on quantifying the time-dependent peel strength and interfacial stability to ensure the performance of DFP-assembled networks in demanding field environments.

Beyond the specific demonstrations presented here, the generality of the DFP assembly paradigm suggests broad applicability to a wide range of fiber-based sensing systems and intelligent structures. The programmatic assembly of high-density sensor networks on complex, non-developable surfaces opens new opportunities in SHM, wearable electronics, soft robotics, and human-machine interaction, where conventional manual assembly imposes severe geometric and functional constraints. While these results underscore the technical versatility of the DFP paradigm, its application in human-centric sensing currently serves as a proof of concept. It should be noted that the biological data for phonation and gesture recognition were collected from a single subject. Since inter-subject variability remains a known challenge in epidermal electronics, future work will focus on multi-subject studies and the development of more robust machine learning models to account for physiological variations. Ultimately, by combining these refined algorithms with technical advancements such as adaptive path optimization and real-time feedback, we can achieve the fully automated assembly of smart sensor surfaces with unprecedented spatial resolution and functional complexity.

## Methods

### Materials and FBG specifications

Commercial single-mode OFs (SMF-28e+^TM^, Corning Incorporated) were used throughout this study, either as bare fibers or with pre-inscribed fiber Bragg gratings (FBGs). The nominal fiber diameter, including the polymer coating, was approximately 242 ± 5 µm. Commercially pre-inscribed FBGs were employed for all functional sensing demonstrations. Each individual grating within the multiplexed arrays featured a length of 5 mm, with their respective central Bragg wavelengths distributed across the 1525–1575 nm range. This work focuses on the programmable assembly and integration of pre-inscribed FBG sensors rather than on optical grating fabrication. Therefore, no additional grating inscription or post-processing steps, such as UV inscription, femtosecond laser inscription, or phase mask-based etching, were performed. All FBGs were used directly without further optical processing. Simultaneously, for preliminary geometric patterning and assembly process development, bare OFs without gratings were employed as mechanically representative and cost-effective substitutes. This strategy facilitated a systematic exploration of routing geometries, bending limits, and multilayer assembly protocols while avoiding the risk of damaging brittle grating regions during the initial path optimization phase. These surrogate OFs ensured that the established minimum-radius assembly framework was rigorously validated before being applied to the functional FBG sensors.

### Adhesive films and interfacial preparation

Three types of adhesive films were employed as substrates for fiber assembly: commercially available optically clear adhesive (OCA), nanotape, and double-sided adhesive tape. Prior to assembly, the adhesive films were laminated onto rigid or curved substrates and subjected to light uniform pressure to eliminate trapped air bubbles. No chemical surface modification or plasma treatment was applied. Importantly, these adhesive layers were not treated as passive bonding interfaces. Instead, they played an active mechanical role in the assembly process by providing sufficient interfacial adhesion to anchor the OF during extraction and assembly. The adhesive force ensures that the fiber can be continuously drawn from the feed and conformally deposited along the prescribed trajectory without slippage, buckling, or loss of positional accuracy, thereby enabling deterministic path definition during five-axis assembly. The selection of adhesive films was based on the conformability and adhesion requirements of different application scenarios. OCA was primarily used for planar geometric patterning demonstrations due to its uniform thickness and optical clarity. Nanotape was employed for conformal assembly on curved and non-developable geometries owing to its enhanced compliance. For epidermal sensing applications on the laryngeal and wrist regions, double-sided adhesive tape was selected to ensure stable interfacial bonding under dynamic physiological deformation. All assembly processes were conducted under ambient laboratory conditions.

### Mechanics-optics coupled modeling of minimum bending radii

To ensure reliable assembly and functional integrity during DFP processing, a mechanics-optics coupled modeling framework was established to quantify the critical bending constraints of a single OF. The analysis assumes linear elastic behavior of the fiber, isotropic and homogeneous adhesive properties, and a uniform stress distribution along the fiber–adhesive interface. Within this framework, three distinct minimum bending radii were defined: an interfacial debonding radius governed by the balance between fiber-induced bending moments and adhesive shear strength (*R*_1min_); a mechanical fracture radius associated with bending-induced tensile stress during fiber guidance and placement (*R*_2min_); and a functional bending radius determined by macrobending-induced optical power attenuation (*R*_3min_). These three constraints collectively delineate the feasible design space for DFP-assembled FBG architectures, including minimum feature size, allowable routing curvature, and achievable sensor density. The resulting models provide first-order design guidelines for programmable fiber assembly on planar and curved substrates. Detailed theoretical derivations, material parameters, and experimental calibration procedures are provided in Supplementary Notes 1, 2.

### DFP assembly system and motion protocol

The DFP assembly paradigm was implemented using a custom-built five-degree-of-freedom manufacturing platform. The hardware integrates three translational axes (*x*, *y*, *z*) and two rotational axes (*V*, *W*), coordinated by a synchronized numerical control system with sub-micron positioning resolution. The precise synchronization and kinematic decoupling of these five axes are achieved through a dedicated coordinate transformation algorithm. The mathematical formulation and implementation details of this five-axis coordination algorithm are provided in Supplementary Note 4, Supplementary Figs. 10, 11. A stainless-steel guiding needle with an internal diameter of 260 μm directed the fiber routing, while moving speeds were maintained between 2 and 5 mm/s. During standard patterning, the initial vertical clearance between the needle tip and the adhesive substrate was set at 730 μm. To prevent layer interference in high-density patterns, the z-axis offset was locally increased by 20 μm at fiber crossover points. Under these parameters, the DFP paradigm demonstrates good assembly efficiency. Representative serpentine arrays (~ 211 mm) and annular paths on complex curved surfaces (~ 350 mm) are precisely integrated within 110 s and 190 s, respectively. Except for deviations resulting from structural compliance and kinematic synchronization constraints, the assembly time remains consistent with theoretical linear predictions (Supplementary Note 4, Supplementary Fig. 12). Since the high precision achieved by DFP on non-developable surfaces fundamentally exceeds the physical limits of manual operation, manual deployment cannot serve as a performance benchmark, highlighting DFP’s unique technical barriers in the digital manufacturing of complex, conformal sensing networks.

**Fig. 5:**
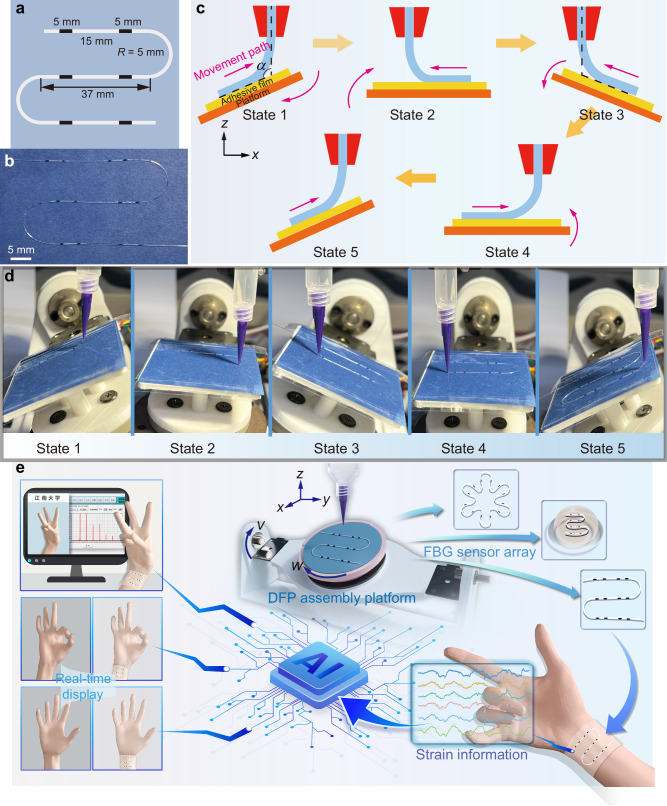
Design, automated direct-FBG-patterning (DFP) assembly, and smart wearable application of the fiber Bragg grating (FBG) sensor array. **a** Schematic layout of the serpentine FBG sensor array, where black line segments represent the pre-inscribed grating sections. **b** Sample image of the assembled FBG array on an adhesive substrate, confirming accurate realization of the designed geometry. The scale bar represents 5 mm. **c** Schematic illustration of the DFP assembly sequence (States 1–5) enabled by coordinated needle moving and substrate rotation. Inclined and horizontal pink arrows represent the movement direction of the needle in each state, while curved pink arrows indicate the rotation direction of the printing platform between consecutive movements. *α* denotes the inclination angle of the platform. Dynamic adjustment of the substrate orientation reduces bending-induced stress in the *x*-*z* plane during the routing of pre-inscribed FBG segments. **d** Sequential experimental photographs demonstrating the synchronous multi-axis coordination of the DFP platform during the assembly process. **e** System-level workflow of the FBG-based smart hand-monitoring system. The DFP-assembled FBG arrays, fabricated using a custom-built five-axis 3D printing system (*x*, *y*, *z*, *V*, *W* axes), are integrated into a wearable device to capture multi-channel strain signals. These signals are subsequently processed by an artificial intelligence (AI)-driven algorithm to achieve real-time gesture recognition and 3D visualization.

A primary challenge in FBG assembly is the inherent brittleness of the grating regions, which are prone to fracture in the *x*-*z* plane during high-curvature patterning. This failure typically stems from stress concentrations at the fiber-substrate contact point and friction within the guiding needle. To mitigate these risks, we implemented a dual strategy of needle lubrication and dynamic angle (*α*) adjustment. As shown in Fig. 5a–d, the assembly of high-fidelity 3 × 2 FBG arrays follows a synchronized five-stage motion protocol: Stage (1) Grating segment laydown. As the needle moves in the positive x direction, the platform rotates 30° clockwise around the U-axis to reach a leading orientation of *α* = 120°. This leading orientation enables the adhesive interface to effectively draw out the pre-inscribed grating segment from the needle tip, ensuring it is laid onto the substrate with minimal localized bending stress; Stage (2) Horizontal transition. Upon completing the grating segment, the platform rotates back to the horizontal position (*α* = 90°) to facilitate the assembly of curved fiber segments; Stage (3) Reverse routing. While the needle moves in the negative x direction, the platform rotates 30° clockwise around the U-axis. This adjustment maintains the optimal extraction angle for the subsequent row of FBGs, ensuring consistent fiber tension and interfacial contact; Stage 4 and 5: Pattern completion. The procedures of Stage (2) and (1) are repeated, respectively, to finalize the high-density array while preserving the mechanical integrity of each sensing node. Throughout the assembly process, optical signals were monitored in real-time using an FBG interrogator with a 1 kHz sampling rate and 1 pm wavelength resolution. To ensure that the DFP process does not compromise the optical functionality of multiplexed FBG arrays, spectral responses were characterized before and after assembly, confirming wavelength stability and negligible channel crosstalk (Supplementary Note 5, Supplementary Fig. 13). This automated strategy successfully suppresses fiber fracture and ensures consistent strain transfer, providing a reliable hardware foundation for subsequent structural health monitoring and intelligent activity recognition.

### Optical interrogation and calibration of FBG arrays

The optical responses of the assembled FBG arrays were recorded using a commercial FBG interrogator (ZX-SL-C16-1K) operating at a fixed sampling rate of 1 kHz or 500 Hz. Bragg wavelength shifts were converted to mechanical strain using the strain sensitivity coefficient of 0.0012 nm/µε, consistent with the specifications of the employed FBGs. All measurements were performed in a temperature-stabilized laboratory environment maintained at 26 ± 0.5 °C. Under these controlled conditions, temperature-induced wavelength variations were negligible compared to strain-induced shifts and were therefore not explicitly compensated.

### Structural health monitoring and MSM algorithm

For the wing-like component SHM (Fig. 3a), a high-fidelity FE model was established to extract its fundamental modal characteristics. The global displacement field, **d**, was reconstructed within the MSM framework, governed by the linear relationship **d** = **Φη**, where **Φ** denotes the modal matrix and **η** represents the modal coordinate vector^15^. To relate the measured strain field, **ε**, with the modal coordinates, a strain modal matrix, **Ψ**, was derived from the FE model such that **ε** = **Ψη**. To achieve high reconstruction accuracy with minimal computational resources, the 3rd, 7th, and 9th torsional modes were strategically selected as the modal basis (Fig. 3b). Real-time 3D deformation was reconstructed by mapping multi-channel strain signals-acquired from the DFP-assembled FBG rosettes onto the modal coordinates through a weighted least-squares estimation. This approach effectively decoupled complex structural responses into a sparse set of modal contributions. The detailed implementation of the displacement reconstruction method, including the construction of the displacement-strain transformation matrix and the full-field response reconstruction process, is elaborated in Supplementary Note 6.

### Intelligent activity recognition and human-machine interaction

An intelligent sensing and decoding framework was developed to process multi-channel strain signals acquired from DFP-assembled FBG arrays on the larynx and wrist. The workflow consists of signal preprocessing, feature extraction, data augmentation, and deep-learning-based classification. An overview of the complete intelligent sensing and decoding pipeline is illustrated in Fig. 5e, highlighting the assembly of FBG arrays, multi-channel strain acquisition, signal interpretation, and real-time human-machine interaction. For phonation recognition, raw strain signals were first denoised using a discrete wavelet transform. Temporal-spectral representations were then generated by extracting MFCCs from the processed signals. To mitigate the limited size of task-specific FBG datasets, a conditional Wasserstein generative adversarial network with gradient penalty (WGAN-GP) was employed to augment the training data while preserving the statistical characteristics of the original signals. The resulting classification performance, including the confusion matrix and feature separability for various vocalization tasks, is illustrated in Supplementary Figs. 14–19. For gesture recognition, strain signals collected from the wrist were preprocessed using moving-average filtering and amplitude normalization to isolate gesture-dependent deformation patterns. Feature sequences were subsequently constructed for temporal classification. Phonation decoding was performed using a three-dimensional convolutional recurrent neural network (3D-CRNN) incorporating a dual-attention mechanism, while gesture recognition employed a convolutional neural network-gated recurrent unit (CNN-GRU) architecture. These models were designed to capture both spatial correlations across FBG channels and temporal dependencies within the strain sequences. The dual-attention mechanism adaptively weighted the contributions of different sensing channels and temporal segments. For real-time human-machine interaction, the predicted activity labels were streamed to a Unity-based client, where they were mapped to corresponding motion states for real-time visualization^37,38^. The framework of the deep learning model, featuring spatiotemporal fusion via CNN and bidirectional long short-Term memory (BiLSTM), along with the GAN-based data augmentation strategy, is detailed in Supplementary Note 7, Supplementary Figs. 20, 21.

## Supplementary information

Supplementary Information

Description of Additional Supplementary File

Supplementary Movie 1

Transparent Peer Review file

## Source data

Source data

## Data Availability

All data supporting the findings of this study are available within the article and its supplementary files. The training dataset for real-time speech recognition and gesture monitoring generated in this study has been deposited in the Figshare database under the identifier 10.6084/m9.figshare.31817992^39^. Any additional requests for information can be directed to and will be fulfilled by the corresponding authors. Source data are provided with this paper.

## Code availability

The deep learning code used for real-time speech recognition and gesture monitoring of this work is available at the Figshare database with identifier 10.6084/m9.figshare.31817992^39^.
