# Supplementary material for: Programmable direct-patterning assembly enables high-density and surface-conformal integration of fiber Bragg grating sensor arrays
Source: Nat Commun. 2026 May 4;17:5998. doi: 10.1038/s41467-026-72613-3 (PMC13346946; doi:10.1038/s41467-026-72613-3)
Supplement: Supplementary file 1 — Supplementary Information [file 41467_2026_72613_MOESM1_ESM.pdf]

Supplementary Information

Programmable direct-patterning assembly enables high-density and surface-conformal integration of fiber Bragg grating sensor arrays

Yin Tao[1,2], Wen Xu[1,2], Peishi Yu[1,2,*], Aoqi Shen[1,2], Yuxiang Zhao[1,2], Xin Zhang[1,2], Maoyang Li[1,2], Junhua Zhao[1,2,*]

[1] *Jiangsu Key Laboratory of Advanced Food Manufacturing Equipment and Technology, School of Mechanical Engineering, Jiangnan University, Wuxi 214122, PR China*

[2] *Jiangsu Province Engineering Research Center of Micro-Nano Additive and Subtractive Manufacturing, Institute of Advanced Technology, Jiangnan University, Wuxi 214122, PR China*

[*]Corresponding authors: ypsnuaa@163.com (P. Yu); junhua.zhao@163.com (J. Zhao).

This Supplementary Information file includes:

Supplementary Notes 1-7

Supplementary Figures 1-21

Supplementary Table 1

Supplementary Movie 1

Supplementary References

Supplementary Note 1. Mechanics-optics coupled framework for critical bending constraints

To ensure the structural integrity and functional reliability of monofil optical fibers (OFs) during the direct FBG pattering (DFP) process, we established a mechanics-optics coupled framework to quantify three distinct failure modes: interfacial debonding, mechanical fracture, and optical functional degradation.

Analytical derivation of the interfacial debonding limit (R_{1min})

The stability of a curved OF on an adhesive substrate is governed by the equilibrium between the fiber's intrinsic elastic restorative force and the interfacial adhesion provided by the adhesive film substrate. As illustrated in Fig. 1b, consider a bent micro-segment of an elastic OF. The small change in arc length (ds) of the curved segment is expressed by Eq. (S1):

$$\mathrm{d}s = R\,\mathrm{d}\theta \tag{S1}$$

where R represents the curvature radius and dθ corresponds to the angular change. The maintenance of this curved geometry requires an interfacial adhesive force per unit area (τ), assumed to be radially directed and distributed circumferentially as Eq. (S2):

$$\tau = \tau_{\max} \cdot \cos\theta \tag{S2}$$

where $\tau_{\max}$ represents the maximum adhesive strength. The bending moment (M_1) induced by this interfacial stress about the symmetry y-axis is derived as Eq. (S3):

$$M_1 = \int_0^{\alpha} \tau_{\max} \cdot \cos\theta \cdot R \cdot \cos\theta \cdot \mathrm{d}A_{\text{contact}} \tag{S3}$$

where A_{contact} is the contact area between the curved segment and the adhesive film which can be expressed as Eq. (S4):

$$\mathrm{d}A_{\text{contact}} = b \cdot \mathrm{d}s = b \cdot R\mathrm{d}\theta \tag{S4}$$

where b is the effective contact width, the integral yields:

$$M_1 = \int_0^{\alpha} \tau \cdot R^2 \cdot b \cdot \cos^2\theta\mathrm{d}\theta = \tau_{\max} \cdot R \cdot b \cdot (\frac{1}{4}\sin 2\alpha + \frac{1}{2}\alpha) \tag{S5}$$

The bending moment per unit angle is defined as $M_2 = M_1/\alpha$. As α approaches zero, the maximum restorative bending moment per unit angle (M_3) is expressed as Eq. (S6):

$$M_3 = \tau_{\max} \cdot R^2 \cdot b \tag{S6}$$

According to Euler-Bernoulli beam theory, the intrinsic bending moment per unit arc length (M_4) required to maintain the fiber's curvature is defined in Eq. (S7):

$$M_4 = \frac{EI}{R} \tag{S7}$$

where E and I denote the Young's modulus and cross-sectional moment of inertia, respectively. For a stable bent pattern, the adhesive interface must satisfy the moment equilibrium $M_4 \leq M_3$. Substituting $I = \pi d_{\mathrm{OF}}^4 / 64$ and solving for R, the minimum curvature radius ($R_{1\mathrm{min}}$) required to prevent interfacial debonding is derived as Eq. (S8):

$$R_{1\min} = \left(\frac{E\pi d_{\mathrm{OF}}^4}{64\tau_{\max} \cdot b}\right)^{\frac{1}{3}} \tag{S8}$$

where d_{OF} is the diameter of OF.

Characterization of the process-induced fracture limit ($R_{2\mathrm{min}}$)

During the DFP process, geometric constraints imposed by the guiding needle tip induce out-of-plane bending in the *x-z* plane. As shown in Supplementary Fig. 1a, the bend radius (R_2) is fundamentally dictated by the relative height (h) between the needle and the adhesion substrate. According to the basic theory of material mechanics, the maximum bending stress ($\sigma_{\max}$) occurring at the fiber surface is expressed as Eq. (S9):

$$\sigma_{\max} = \frac{E \cdot r}{R_2} \tag{S9}$$

where r represents the radius of the OF. To prevent brittle failure, $\sigma_{\max}$ must remain below the intrinsic bending strength of the OF (σ_{OF}). Consequently, the minimum radius of curvature ($R_{2\mathrm{min}}$) required to ensure process stability is defined as Eq. (S10):

$$R_{2\min} = \frac{Er}{\sigma_{\mathrm{OF}}} \tag{S10}$$

Modeling of optical functional integrity ($R_{3\mathrm{min}}$)

As shown in Supplementary Fig. 1b, beyond mechanical considerations, macrobending of the OF induces functional degradation through optical power attenuation. The relationship between the bending loss coefficient (β) and the bend radius (R_3) for step-index single-mode fibers follows an exponential trend[1-3], as shown in Eq. (S11):

$$\beta = \frac{A}{\sqrt{R_3}} \cdot \mathrm{e}^{B \cdot R_3} \tag{S11}$$

where A and B are empirical constants determined by the OF type and the operational wavelength of the light source. For a curved OF segment with a total bending angle proportion N (where $N = \theta / 360°$), the cumulative optical loss L_{total} is expressed as Eq. (S12):

$$L_{\text{total}} = \beta \cdot (2\pi R_3 N) = C \cdot N\sqrt{R_3} \cdot e^{-B \cdot R_3} \tag{S12}$$

where C represents the normalized loss coefficient. To ensure reliable sensing performance and signal-to-noise ratio in the grating region, the output-to-input power ratio (P_1/P_0) must satisfy a minimum functional threshold K, as shown in Eq. (S13):

$$\frac{P_1}{P_0} = 10^{-\frac{L_{\text{total}}}{10}} \geq K \tag{S13}$$

By substituting Eq. (S12) into Eq. (S13) and solving for R_3 using the Lambert W function (W), the minimum functional radius ($R_{3\min}$) is analytically derived as Eq. (S14):

$$R_{3\min} = -\frac{1}{2B} \mathrm{W}\left(-2B(\frac{-10\log_{10} K}{C \cdot N})^2 \right) \tag{S14}$$

Supplementary Note 2. Determination of key parameters for the coupled framework

Characterization of mechanical and interfacial properties

The geometric and mechanical parameters required for model evaluation were experimentally characterized. As shown in Supplementary Fig. 2a, optical microscopy (OM) was used to determine the cross-sectional structure of the OF, revealing an acrylate coating and a silica cladding with diameters of $d_c = 248.19 \pm 2.5$ μm and $d_f = 126.33 \pm 1.2$ μm, respectively.

Quasi-static tensile tests were initially performed in triplicate following ASTM C1557-14 to estimate the elastic modulus. The linear elastic regime yielded a Young's modulus (E) of approximately 16880 ± 733 MPa. However, post-peak stress reduction accompanied by coating-cladding separation indicated that tensile failure was governed by interfacial debonding rather than intrinsic fiber fracture. Therefore, tensile tests were unsuitable for evaluating the intrinsic fracture strength of the OF (σ_{OF}). To accurately determine the σ_{OF}, two-point bending tests were conducted. The OF was constrained within FDM-printed grooved fixtures ($d_g = 0.3$ mm) and subjected to displacement-controlled compression until fracture occurred (Supplementary Fig. 2b). The fracture strength was calculated from the measured plate separation (d) at failure using the classical two-point bending relationship[4], as shown in following Eq. (S15):

$$\sigma_{OF} = \frac{1.198E \cdot d_f}{d - d_c + 2d_g}\left(1 + \frac{2.546 d_f}{d - d_c + 2d_g}\right) \quad \text{(S15)}$$

Based on five independent measurements, the values of d and the corresponding σ_{OF}, derived from a series of two-point bending experiments, are summarized in Supplementary Fig. 2c. The fracture strength was calculated as $\sigma_{OF} = 1297 \pm 13$ MPa, which was subsequently used for evaluating the R_{2min} model.

The τ_{max} between the OF and various adhesive substrates was characterized using a standardized single-lap shear test[5]. As illustrated in Supplementary Fig. 2d, a double-sided optically clear adhesive (OCA, 3M8146-5) was bonded to a glass substrate. One end of the OF was bonded to the OCA film with a controlled contact length of $L = 15$ mm, while the opposing end was secured to a separate glass substrate using a

cyanoacrylate adhesive (YNT-30). To enhance the interfacial force signal and ensure measurement reliability, a dual-OF design was employed. The effective contact width for a single OF on the OCA film (b_{OCA}) was measured via OM as 81.13 ± 4.58 μm. Prepared samples were loaded in a universal testing machine at a constant displacement rate of 0.5 mm/min. The τ_{max} was calculated from the peak tensile force (F_{max}) using the Eq. (S16):

$$\tau_{max} = \frac{F_{max}}{2Lb} \tag{S16}$$

Based on three valid samples, the $\tau_{max\text{-}OCA}$ for the OF/OCA interface was determined to be 0.45 ± 0.02 MPa (Supplementary Fig. 2e). The same methodology was applied to evaluate the adhesion strength at the OF/nanotape and OF/double-sided tape interfaces (Supplementary Fig. 3a). OM revealed mean contact widths of b_{nano} = 201.26 ± 0.56 μm and b_{double} = 112.67 ± 7.28 μm, respectively. Representative force-displacement curves for these interfaces are demonstrated in Supplementary Figs. 3b, c. Consequently, the maximum interfacial shear strengths were determined to be $\tau_{max\text{-}nano}$ = 1.41 ± 0.08 MPa and $\tau_{max\text{-}double}$ = 0.86 ± 0.03 MPa, respectively.

To determine the optical attenuation parameters involved in the macrobending loss model, independent bending experiments were first conducted on single-mode optical fibers under controlled conditions. As shown in Supplementary Fig. 4a, the OF was wound once around cylindrical mandrels with different radii, and the transmitted optical power was measured using a constant-wavelength light source and an optical power meter. For each bending radius, measurements were repeated five times to ensure statistical reliability. The experimental data, representing bending loss per unit length versus the bend diameter, were plotted with error bars as shown in Supplementary Fig. 4b. By substituting these experimental results into the exponential relationship defined in Eq. (S11), the empirical parameters for the OF were determined as A = 13.0277 and B = -0.5391. Consequently, the calibrated formula for the loss per unit length is established as Eq. (S17):

$$\beta = \frac{13.0277}{\sqrt{R_3}} \cdot \mathrm{e}^{-0.5391 \cdot R_3} \tag{S17}$$

Overall, the specific mechanical and geometric parameters related to the OF (SMF-28e+^{TM}, manufactured by Corning Incorporated) involved in the above equations are shown in Supplementary Table 1.

Verification of multiple theoretical models

To experimentally identify the minimum in-plane bending radius required for stable fiber adhesion, OFs were patterned into arc-shaped trajectories with progressively decreasing radii on three representative adhesive substrates: OCA, nanotape, and double-sided tape. The OFs were continuously guided and bonded onto the adhesive films using the DFP process at a constant moving speed, while the platform executed preprogrammed planar trajectories. As shown in Supplementary Fig. 5, OFs patterned with relatively large bending radii exhibited stable adhesion and well-preserved geometries on all substrates. However, when the bending radius was reduced below a substrate-dependent threshold, localized interfacial slippage and partial debonding were consistently observed after fabrication. Based on multiple experiments, the experimentally obtained minimum radii ($R_{1\text{min-e}}$) ensuring reliable adhesion were experimentally determined to be approximately 5 mm for OCA, 2.2 mm for nanotape, and 3.5 mm for double-sided tape. These experimentally identified thresholds define the practical in-plane geometric limits for DFP assembly on different adhesive substrates. Based on the experimentally identified adhesion limits, the theoretical predictions of the $R_{1\text{min}}$ model were evaluated for different adhesive substrates. The interfacial shear strength parameters extracted from single-lap shear tests were substituted into Eq. (S8) to calculate the theoretical minimum bending radii ($R_{1\text{min-t}}$). As summarized in Supplementary Fig. 6a, the $R_{1\text{min-t}}$ values for OCA, nanotape, and double-sided tape show close agreement with the experimentally measured thresholds, with relative errors (RE) below 11%. This strong consistency validates the high predictive accuracy of the proposed mechanics model in determining the critical $R_{1\text{min}}$ required to ensure structural stability during the DFP assembly process.

The $R_{2\text{min}}$ model, which describes OF fracture induced by out-of-plane bending during deposition, was validated by systematically reducing the vertical distance between the needle and the adhesive substrate. As illustrated in Supplementary Fig. 6b,

decreasing the height h led to progressively smaller bending radii in the x-z plane until brittle fracture occurred. Experimentally, fracture was observed when the bending radius reached approximately $R_{2min\text{-}e}$ = 1.59 mm, corresponding to a critical height of 730 μm. The theoretical prediction yielded $R_{2min\text{-}t}$ = 1.64 mm, resulting in a *RE* of approximately 3%. This agreement verifies the validity of the fracture-based bending model for predicting process stability during DFP assembly.

To validate the R_{3min} imposed by macrobending loss, optical power attenuation experiments were performed on the actual DFP-patterned structures. It is important to distinguish this validation from the initial calibration procedure. The initial calibration process involved winding the OF a full 360° around cylinders to extract parameters A and B, while this validation test measures the real-time loss of fabricated semicircular patterns ($N = 0.5$) with varying radii R_3. The experimentally measured loss values were compared with the theoretical predictions derived from the empirical attenuation model. As shown in Supplementary Fig. 6c, the measured optical loss closely follows the theoretical curve over the tested bending radius range, with an average *RE* of approximately 10%. The experimental results clearly demonstrate that as the bending radius R_3 decreases, the optical loss increases significantly, following the predicted exponential trend. The strong agreement between the measured data and the theoretical curve confirms that our model is reasonable and capable of accurately predicting optical attenuation across different radii. This confirms the model's reliability in determining the functional routing limits required to maintain signal integrity for multiplexed FBG arrays.

In summary, the critical minimum radii derived from the proposed mechanics-optics coupled framework have been validated across various adhesive substrates. A comprehensive comparison between the theoretical predictions and experimental measurements is presented in Table 1 of the main manuscript. These validated thresholds provide essential design criteria and operational guidelines to ensure the structural and functional integrity of the FBG sensors during the automated DFP process.

Supplementary Note 3. Versatility and adaptability of the DFP paradigm

The DFP paradigm enables programmable assembly of customized sensor layouts with diverse planar geometries. As illustrated in Supplementary Fig. 7, the automated system reliably executes a range of predefined paths, including alphanumeric paths (e.g., "JNU") and representative geometric patterns such as multi-radius spirals and star-shaped patterns. These examples demonstrate the capability of DFP to achieve OF curvature layouts. In addition, a spatial cross-fiber assembly strategy is employed to construct high-density sensing networks, exemplified by grid-like arrays composed of multiplexed FBGs. This approach allows deterministic control over fiber routing and intersection spacing, enabling sensing layouts to be adapted to specific structural geometries and multi-point monitoring requirements.

Simultaneously, DFP enables conformal assembly of FBGs on curved surfaces, extending beyond the constraints of conventional planar fabrication. As shown in Supplementary Fig. 8, the five-axis assembly platform leverages coordinated rotational and translational kinematics to guide FBG fibers along prescribed three-dimensional trajectories. This capability allows controlled deposition of FBGs into spatially curved and helical patterns while maintaining stable adhesion and mechanical integrity on non-planar substrates.

As shown in Supplementary Figs. 9a and b, on planar composite substrates, DFP supports the assembly of complex layouts, including orthogonal grid and spiral patterns. The spiral pattern demonstrates that path-defined routing can accommodate structural features such as perforated laminates, allowing the sensing layout to be adaptively designed according to the actual geometry of the substrate. In addition, conformal assembly on curved substrates is achieved through coordinated multi-axis motion, enabling precise OF placement on stainless-steel and foam hemispheres (Supplementary Figs. 9c and d). These demonstrations indicate that DFP is compatible with a wide range of substrate materials and geometries, and is not limited to planar fabrication, providing a general assembly strategy for integrating fiber-based sensors on complex engineering surfaces.

Supplementary Note 4. Five-axis kinematic control and conformal trajectory planning for DFP assembly

Direct conformal trajectory planning on curved surfaces

Conformal fiber assembly on curved substrates requires motion trajectories that intrinsically follow surface geometry. Conventional approaches typically rely on a two-step strategy, where a planar trajectory is first designed and then projected onto a three-dimensional surface[6]. Such indirect projection inevitably introduces geometric distortion and trajectory twisting, which degrade placement accuracy and conformality. To address this limitation, a direct conformal trajectory planning strategy was adopted. As shown in Supplementary Fig. 10, the procedure consists of four sequential steps. Step 1: Starting from the boundary of the curved surface, a set of concentric *V*-direction curves is generated by proportionally scaling the edge line toward the surface centroid. This operation preserves the global geometry of the surface while defining a structured set of reference curves. Step 2: Each *V*-direction curve is uniformly divided into the same number of discrete points. These points are organized into a matrix, denoted as *Point*, where each row corresponds to a *V*-direction curve and each column represents points at the same parametric position along different curves. Step 3: By transposing the *Point* matrix to obtain $Point^T$, points sharing the same column index are interconnected, forming a family of *W*-direction curves. This matrix transposition operation establishes orthogonal curve sets without introducing geometric projection error. Step 4: The intersection of the *W*- and *V*-direction curves yields a structured surface mesh that conforms to the original geometry. Based on the desired sensing layout, selected mesh nodes are connected to define a polyline directly on the surface. The polyline is subsequently smoothed to obtain a continuous conformal trajectory. Finally, the conformal trajectory is offset along the local surface normal by a prescribed distance to define the motion path of the guiding nozzle during fiber assembly. This mesh-based planning approach enables precise, distortion-free trajectory generation on complex curved surfaces.

Five-axis kinematic platform and motion principle

To realize conformal deposition along spatial trajectories, a five-axis motion system

was employed. The system consists of three translational degrees of freedom (x, y, z) and two rotational degrees of freedom (V, W), enabling synchronized control of nozzle position and orientation[7,8]. As shown in Supplementary Fig. 11a, a global Cartesian coordinate system is defined with respect to the motion platform. For an arbitrary target point on the substrate surface, its spatial position is expressed as $\mathbf{P} = (x_1, y_1, z_1)$, and the corresponding surface normal vector is given by $\mathbf{L} = (i, j, k)$, which is obtained directly from the surface mesh during trajectory planning. Maintaining alignment between the nozzle axis and the local surface normal is essential to ensure stable contact conditions and minimize bending-induced stress during OF assembly. Through coordinated rotations about the V- and W-axes, the substrate is reoriented such that the local surface normal aligns with the global z-axis, as schematically shown in Supplementary Fig. 11b. After rotation, the transformed normal vector becomes $\mathbf{L}' = [0, 0, (i^2 + j^2 + k^2)^{1/2}]$. The required rotation angles θ_V and θ_W are determined from the components of the surface normal vector by appling this alignment condition. The corresponding rotation matrices are denoted as $\mathbf{R}(\theta_V)$and $\mathbf{R}(\theta_W)$, which describe rotations about the V- and W-axes, respectively. The overall normal transformation can thus be expressed as Eq. (S18) ~ Eq. (S21):

$$\mathbf{L}' = \mathbf{R}'(\theta_W) \bullet \mathbf{R}(\theta_V) \bullet \mathbf{L} \tag{S18}$$

$$\mathbf{R}'(\theta_W) = \mathbf{R}(\theta_W) \bullet \mathbf{R}(\theta_V) \tag{S19}$$

$$\theta_V = \arccos\left(\frac{k}{\sqrt{i^2 + j^2 + k^2}}\right) \tag{S20}$$

$$\theta_W = \arctan(i/j) \tag{S21}$$

Accurate coordinate transformation is essential for maintaining spatial synchronization between the nozzle and the curved substrate during the DFP process. When the substrate undergoes a rotation of θ_V around V-axis, the underlying base coordinate system shifts, causing the W rotation axis to deviate from its initial alignment with the global z-axis. Consequently, the rotation around the W-axis must be described by the modified transformation matrix $\mathbf{R}'(\theta_W)$, as defined in Eq. (S19). By applying these sequential transformations, the target point P within the local frame is mapped to

its absolute coordinates $\mathbf{P'} = (x, y, z)$ in the global reference frame, as shown in Eq. (S22):

$$\mathbf{P'} = \mathbf{R'}(\theta_{\mathrm{W}}) \bullet \mathbf{R}(\theta_{\mathrm{V}}) \bullet \mathbf{P} \tag{S22}$$

Assembly efficiency analysis of the DFP paradigm

To establish a quantitative benchmark for process scalability, the total assembly time was systematically analyzed for diverse geometric trajectories. As illustrated in Supplementary Figs. 12a and b, two representative layouts were investigated: a planar serpentine trajectory (total length $L \approx 211$ mm) and a complex curved annular trajectory (total length $L \approx 350$ mm), with feed speeds (v) set at 2 mm/s and 5 mm/s, respectively. For the planar structure, the measured assembly times were 107 s and 41 s at feed speeds of 2 mm/s and 5 mm/s, respectively. For the curved structure, the corresponding assembly times were 187 s and 76 s. A comparison between the experimental results and theoretical predictions (L/v, Supplementary Fig. 12c) shows good agreement under planar conditions. The slight deviations observed during curved surface integration are mainly attributed to the structural compliance of the custom-built five-axis platform and kinematic synchronization constraints during multi-axis coordinated interpolation. While minor discrepancies exist within acceptable limits, the assembly time remains highly predictable and scales linearly with path length and feed speed. Such consistency demonstrates the robust scalability of the DFP paradigm for large-area integrated sensing networks. Furthermore, a direct comparison with manual assembly was not performed, as the high-precision patterned integration achieved here, particularly on non-developable surfaces, fundamentally exceeds the physical limitations of conventional manual positioning and bonding. This highlights the unique advantage of the DFP process in enabling high-fidelity conformal sensing networks that are unattainable through traditional manual approaches.

Supplementary Note 5. FBG optical interrogation and strain measurement

The optical responses of the DFP-assembled FBG arrays were assembled using a commercial FBG interrogator (ZX-SL-C16-1K). All Bragg wavelength signals were recorded at a constant sampling frequency of 1 kHz, enabling the capture of both quasi-static and dynamic strain responses. To convert the measured Bragg wavelength shifts into axial strain, a linear strain-wavelength relationship was adopted as Eq. (S23)[9-11]:

$$\varepsilon = \frac{[\Delta\lambda_{\varepsilon} \text{-} \Delta\lambda_{\mathrm{T}} \cdot a]}{K_{\varepsilon}} \tag{S23}$$

where K_{ε} represents the strain sensitivity coefficient of the FBG, approximately 1.2 pm/με; $\Delta\lambda_{\varepsilon}$ and $\Delta\lambda_{\mathrm{T}}$ denote the Bragg wavelength shifts induced by strain and temperature variations, respectively; and a is the temperature-dependent grating coefficient. In this study, all experiments were conducted at a constant ambient temperature of approximately 26 °C. Under these stable thermal conditions, the $\Delta\lambda_{\mathrm{T}}$ remains within a very small range and can be reasonably neglected, allowing the sensing response to be attributed primarily to mechanical strain. Despite this laboratory-scale simplification, temperature sensitivity remains an inherent factor for the practical deployment of FBG sensors. To provide a quantitative assessment for field applications, based on the typical temperature sensitivity of silica FBGs (10 pm/°C), a mere 1°C temperature fluctuation would introduce an apparent strain crosstalk of approximately 8.3 με[12,13]. While this value is relatively minor compared to the high-amplitude strains recorded in our wing structural monitoring, it poses impact on capturing subtle physiological micro-vibrations. Consequently, in our current experimental validation for phonation and gesture recognition, maintaining a constant laboratory ambient temperature to eliminate thermal interference is essential for ensuring signal integrity and verifying process reliability. Furthermore, for complex application scenarios involving drastic thermal fluctuations, the DFP assembly paradigm exhibits excellent compatibility. Its high-precision programmable layout allows for the precise integration of reference gratings or the construction of differential measurement arrays within the same conformal layer, providing a robust hardware foundation for reliable signal decoupling.

To characterize the signal integrity and spectral evolution of the assembled sensors, the reflected spectra of multiplexed FBG arrays were systematically monitored across diverse routing geometries (Supplementary Fig. 13). Two distinct FBG patterns were evaluated using an optical sensing interrogator: 2 × 2 arrays featuring a single bend (R_3 = 4 mm or 5 mm) and 3 × 2 arrays incorporating dual bends of equal radius (R_3 = 5 mm or 6 mm). Experimental results revealed that for single-bend patterns with R_3 = 5 mm, the optical power attenuation across downstream gratings remained within acceptable limits for reliable data acquisition. In contrast, reducing the radius to 4 mm induced severe macrobending losses, with the relative power ratio dropping below 0.2, which significantly degrades the signal fidelity. A similar trend was observed in double-bend patterns. While R_3 = 5 mm resulted in substantial power degradation at terminal gratings, the 6 mm radius maintained sufficient signal strength. Based on these empirical findings, a bend radius of 5 mm was selected for single-bend layouts, while 6 mm was adopted for double-bend designs. This selection achieves an optimal balance between device compactness and sensing reliability. Furthermore, these results provide experimental validation for the R_{3min} theoretical model, confirming its utility in guiding the design of functional, high-density FBG sensing networks.

Supplementary Note 6. Displacement reconstruction method

The modal superposition method (MSM) is commonly employed due to its convenience for modal analysis and its suitability for three-dimensional structures[14]. In a linearly elastic, damped system with multiple degrees of freedom, the overall structural response can be represented as the superposition of the individual modal contributions. Consequently, the displacement and strain of the structure, obtained through modal superposition, are expressed in Eqs. (S24) and (S25):

$$d(x) = \sum_{i=1}^{n} \Phi_i(x)\eta_i \tag{S24}$$

$$\varepsilon(x) = \sum_{i=1}^{n} \Psi_i(x)\eta_i \tag{S25}$$

where $d(x)$ and $\varepsilon(x)$ denote displacement and strain responses at position x, respectively; Φ_i and Ψ_i represent the modal displacement and strain of the i-th order at the position x; q_i is the corresponding the i-th order modal coordinate, and n indicates the total number of selected modes. For a structure equipped with M strain measurement points, the strain response can be expressed in matrix form as Eq. (S26):

$$\{\boldsymbol{\varepsilon}\}_{M\times1} = [\boldsymbol{\Psi}]_{M\times n} \cdot \{\boldsymbol{\eta}\}_{n\times1} \tag{S26}$$

where $\{\boldsymbol{\varepsilon}\}_{M\times1}$ means the strain response vector, $[\boldsymbol{\Psi}]_{M\times n}$ is the strain mode matrix composed of n-th order modes, and $\{\boldsymbol{\eta}\}_{n\times1}$ is the modal coordinate vector. Thus, the modal coordinates can be obtained as Eq. (S27):

$$\{\boldsymbol{\eta}\}_{n\times1} = \left([\boldsymbol{\Psi}]_{M\times n}^{T} \cdot [\boldsymbol{\Psi}]_{M\times n}\right)^{-1} \cdot [\boldsymbol{\Psi}]_{M\times n}^{T} \{\boldsymbol{\varepsilon}\}_{M\times1} \tag{S27}$$

Consequently, the displacement field of the structure can be described by Eqs. (S28) and (S29):

$$[\mathbf{T}]_{N\times M} = [\boldsymbol{\Phi}]_{N\times n} \cdot \left([\boldsymbol{\Psi}]_{M\times n}^{\mathrm{T}} \cdot [\boldsymbol{\Psi}]_{M\times n}\right)^{-1} \cdot [\boldsymbol{\Psi}]_{M\times n}^{\mathrm{T}} \tag{S28}$$

$$\{\mathbf{d}\}_{N\times1} = [\mathbf{T}]_{N\times M} \cdot \{\boldsymbol{\varepsilon}\}_{M\times1} \tag{S29}$$

where $[\mathbf{T}]_{N\times M}$ is the displacement-strain transform (DST) matrix, N is the number of displacements.

According to the derived formulation, the procedure for displacement reconstruction

via the MSM is conducted as follows: First, mechanical characterization of the selected wing-like structure is performed to determine its fundamental material properties, and a corresponding finite element model is established. Subsequently, modal analysis is carried out to extract both displacement and strain mode shapes of the structure. A displacement-strain transformation matrix is then constructed to compute displacements at various points based on measured strain data. Finally, the acquired strain measurements are applied to the DST matrix to reconstruct the full-field displacement response of the structure. This modal transformation approach is not limited to one-dimensional beam analyses and offers distinct advantages for complex mechanical structures and direct surface reconstruction. It allows for accurate reconstruction of the full displacement field using a limited number of sensors.

Supplementary Note 7. Deep learning-enhanced intelligent recognition for wearable sensing

Phonation signal extraction and deep learning framework

Supplementary Figs. 14-19 systematically present the characteristic strain responses and corresponding multi-dimensional feature representations for six target words (‘And’, ‘No’, ‘Not’, ‘Ok’, ‘So’, and ‘Yes’) acquired by the 3 × 2 FBG array during laryngeal phonation. To ensure statistical robustness and provide a comprehensive dataset for the deep learning model, each word was pronounced more than 200 times, with representative repetitions illustrated in the figures to demonstrate signal consistency. For each word, the figures visualize the raw temporal strain profiles, the preprocessed time-frequency representations extracted using Mel-frequency cepstral coefficients (MFCC), and the distribution of four key statistical descriptors (mean, standard deviation (STD), skewness, and kurtosis) across the six sensing channels.

Despite the inherent complexity of physiological vibrations, the FBG array consistently captured highly repeatable spatiotemporal strain patterns that are distinct across different words. The observed feature separability across all repeated trials and sensing elements confirms the ability of the sensors to distinguish subtle differences in vocal fold dynamics. These results validate the robustness of the signal acquisition and feature extraction pipeline and provide a reliable foundation for subsequent deep-learning-based phonation decoding.

While these distinctive features provide a reliable foundation, the subtle differences in phonemic micro-vibrations and the high degree of feature coupling necessitate a sophisticated decoding framework to achieve high-accuracy, non-acoustic speech recognition in noisy or privacy-sensitive environments. Consequently, a non-acoustic speech recognition framework was developed based on epidermal strain signals acquired by a multi-channel FBG array. Supplementary Fig. 20 illustrates in detail the signal processing flow, data augmentation strategies, and deep learning framework used for speech recognition.

A 3 × 2 FBG array conformally attached to the laryngeal region was used to capture multi-channel strain signals during phonation at a sampling rate of 1 kHz. Each sensing

channel recorded dynamic epidermal strain induced by laryngeal muscle activity during speech production. Raw strain signals were first subjected to baseline correction and denoising using discrete wavelet decomposition (Daubechies-4 wavelet, five-level decomposition) to suppress low-frequency drift and high-frequency noise while preserving speech-related vibration components. The processed signals were then amplitude-normalized to eliminate inter-trial variability and ensure consistency across samples. To fully characterize the temporal-spectral properties of the strain signals, both frequency-domain and time-domain features were extracted. Frequency-domain features were obtained using MFCC. The processing steps included pre-emphasis (coefficient 0.97), framing with a frame length of 40 ms and a frame shift of 20 ms. The first 13 cepstral coefficients were retained, followed by cepstral liftering (lifter coefficient = 22) to enhance high-frequency discrimination. In parallel, four statistical descriptors (mean, STD, skewness, and kurtosis) were computed for each channel in the time domain. The MFCC features and statistical descriptors were concatenated to form a composite feature representation. For each speech sample, the resulting feature tensor had a dimension of (6 channels, 17 features, 399 time frames), preserving both spatial and temporal information.

Given the limited size of the physiological speech dataset, a conditional Wasserstein generative adversarial network with gradient penalty (WGAN-GP) was employed for data augmentation. The generator received a 100-dimensional Gaussian noise vector conditioned on a 50-dimensional embedded class label and produced synthetic feature tensors with the same dimensionality as the real data ($6 \times 17 \times 399$). The network structure consisted of three successive upsampling blocks. The discriminator comprised four convolutional downsampling blocks with channel sizes of 32, 64, 128, and 256, followed by adaptive pooling and a fully connected layer to output the Wasserstein distance. Gradient penalty with a coefficient $\lambda = 10$ was applied to enforce the Lipschitz constraint, and the discriminator and generator were trained with a 5:1 update ratio. Using this strategy, 500 synthetic samples were generated for each speech category, expanding the training dataset by approximately one order of magnitude and effectively reducing overfitting. Speech classification was performed using a convolutional

recurrent neural network (CRNN) integrated with temporal and channel attention mechanisms. The input to the network was the standardized feature tensor with dimensions [C, 1, 6, 17, 399], where C denotes the batch size, and the second dimension represents a single input channel.

A 3D convolutional module was first employed to extract joint spatial-temporal features across sensor channels and time. The module consisted of two convolutional layers with 32 and 64 kernels of size 3 × 3 × 3, respectively. Each layer was followed by batch normalization, ReLU activation, dropout (0.3 and 0.2), and max pooling with a kernel size of 1 × 2 × 2. The output feature map was reduced to a dimension of [C, 64, 6, 4, 99]. The feature maps were then reshaped into a sequence of size [C × 6, 99, 256] and fed into a two-layer bidirectional long short-term memory (LSTM) with 256 hidden units and a dropout rate of 0.3 to model long-term temporal dependencies. To enhance discriminative capability, dual attention mechanisms were introduced. Temporal attention was implemented to assign adaptive weights to different time steps, allowing the model to focus on speech-critical segments. Channel attention was applied to dynamically reweight the contributions of the six FBG sensors, reflecting their relative importance during phonation. The attention-weighted features were aggregated into a 512-dimensional context vector. Finally, the aggregated features were passed through a fully connected classifier (512→ 256 → 128 → 6) with a Softmax output layer to predict the probability distribution over six target words (“And”, “No”, “Not”, “Ok”, “So”, and “Yes”).

Intelligent hand gesture classification and kinematic decoding

As illustrated in Supplementary Fig. 21a, characteristic strain responses of the 3 × 2 FBG array were recorded during repetitive execution of three representative gestures (Gesture-4, Gesture-5, and Gesture-6). Each sensing element captured distinct time-varying epidermal strain patterns associated with specific muscle activations, yielding repeatable and gesture-dependent signal signatures. To ensure statistical robustness, approximately 40 repetitions were recorded for each gesture type. The high strain sensitivity of the DFP-assembled FBG sensors enables reliable detection of subtle musculoskeletal dynamics, providing suitable input data for gesture classification.

To decode the multi-channel temporal strain signals generated during complex hand movements, a hybrid deep learning framework combining convolutional neural networks (CNNs) and long short-term memory (LSTM) networks was developed[15,16], as shown in Supplementary Fig. 21b. The architecture is designed to jointly capture spatial distribution characteristics across sensing channels and temporal evolution patterns within each gesture. The input to the network consists of six-channel raw temporal strain signals with a dimensionality of [C, 6, T], where B denotes the batch size and T the number of time steps. The signals are first processed by a CNN-based feature extraction branch. A one-dimensional convolutional layer with a kernel size of 11 and stride of 2 expands the feature space to 64 channels, enabling the capture of coarse temporal patterns over broad time windows.

Subsequently, the features are passed through three cascaded temporal feature extraction blocks based on depthwise separable convolutions, which decompose standard convolutions into depthwise and pointwise operations to reduce parameter complexity while preserving expressive power. These blocks progressively increase the channel dimensions to 128, 256, and 512, forming a multi-scale representation capable of modeling both short-term fluctuations and long-term gesture dynamics. Each block incorporates residual connections to stabilize training and integrated spatio-temporal attention mechanisms to adaptively emphasize informative time steps and feature channels. To accommodate variable-length gesture inputs, an adaptive max-pooling layer compresses the temporal dimension to a fixed length of 128, yielding a feature map of size [C, 512, 128]. This design ensures consistent computational complexity and facilitates real-time deployment. The feature map is then transposed to [C, 128, 512] and fed into a four-layer bidirectional LSTM (BiLSTM) network with a hidden size of 256. The bidirectional structure enables the model to capture contextual dependencies from both past and future temporal directions, which is critical for accurately modeling the complete progression of gesture execution. The BiLSTM output is further refined using dual attention mechanisms. Temporal attention assigns importance weights to different time steps to emphasize discriminative motion phases, while channel attention recalibrates the contributions of different feature dimensions. The resulting attention-

weighted representation is subsequently passed through a three-layer fully connected classifier incorporating batch normalization, GELU/SiLU activation functions, and dropout regularization to map the extracted features to specific gesture categories. To ensure stable and efficient training, Kaiming normal initialization was applied to convolutional layers, and orthogonal initialization was used for LSTM weights, with the forget gate bias set to 1.0 to enhance long-term memory retention. By combining CNN-based spatial feature extraction with BiLSTM-based temporal modeling, the proposed framework provides an effective and computationally efficient solution for decoding multi-channel physiological micro-strain signals into distinct hand gesture labels.

Supplementary Table

Table 1 Geometrical and mechanical parameters related to OF

Parameters	Symbol	Unit	Mean Value
Young's modulus	E	MPa	16880
Diameter of the OF	d_{OF}	μm	252.19
	$\tau_{\mathrm{max\text{-}OCA}}$		0.45
Maximum interface strength	$\tau_{\mathrm{max\text{-}nano}}$	MPa	1.41
	$\tau_{\mathrm{max\text{-}double}}$		0.86
	b_{OCA}		81.13
Contact width	b_{nano}	μm	201.26
	b_{double}		112.07
Bending strength of OF	σ_{OF}	MPa	1297

Supplementary Figures

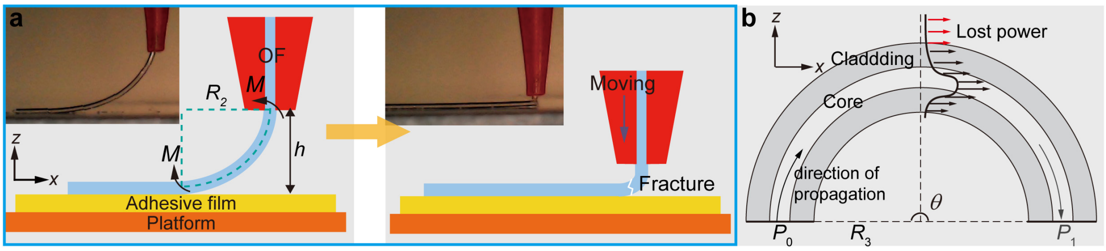

Supplementary Fig. 1 Mechanical and optical constraints in the direct-FBG-patterning (DFP) process. a Process-induced OF fracture in the *x*-*z* plane. A reduction in the relative height *h* between the deposition needle and the substrate decreases the bend radius R_2, leading to an increased bending moment *M* and subsequent brittle fracture of the OF. **b** Schematic illustration of macrobending loss within the fiber core and cladding. P_0 and P_1 denote the input and output optical power, respectively, where the functional power loss depends on the macroscopic bending radius R_3 and bending angle θ.

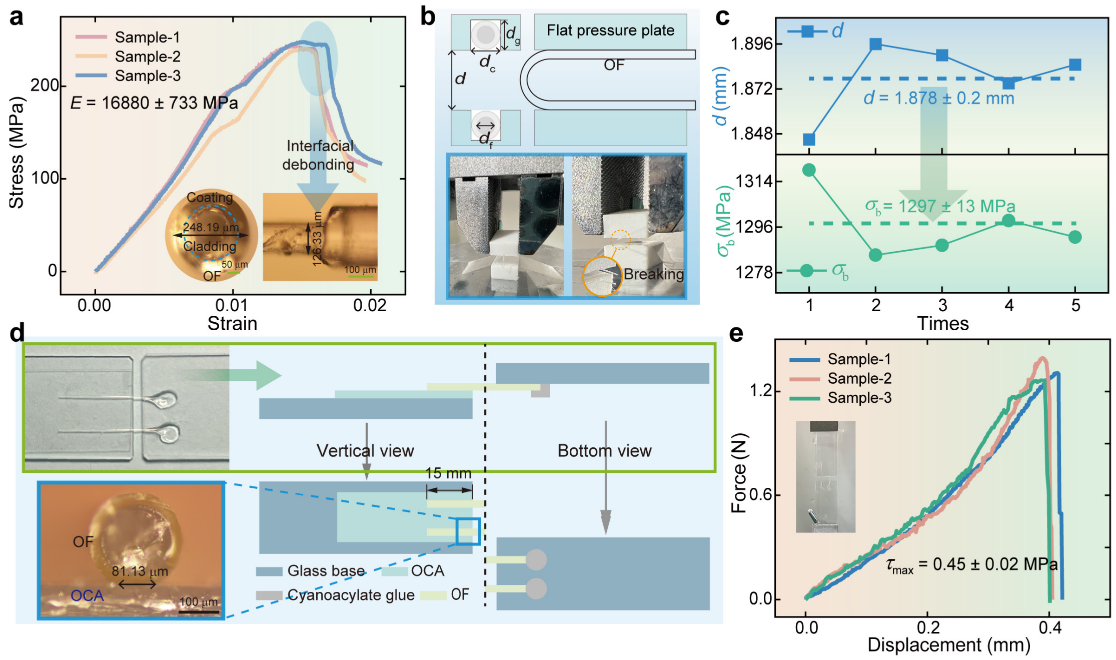

Supplementary Fig. 2 Experimental characterization of the mechanical properties and interfacial adhesion of the optical fiber (OF). a Tensile stress-strain curves of the OF for three representative samples. The Young's modulus is determined as E = 16880 ± 733 MPa. The inset provides an optical micrograph of the OF cross-section, showing the acrylate coating (d_c = 248.19 μm) and silica cladding (d_f = 126.33 μm). **b** Schematic and experimental photographs of the bending test setup used to determine the intrinsic bending strength and critical failure radius of the OF. **c** Statistical measurements of the critical bending diameter d (1.878 ± 0.2 mm) and the corresponding bending strength σ_b (1297 ± 13 MPa) over five independent trials. **d** Detailed characterization of the interfacial bonding between the OF and optically clear adhesive (OCA). The schematics illustrate the vertical and bottom views of the test specimen preparation using a glass base and cyanoacrylate glue. The inset shows a magnified cross-sectional view of the OF partially embedded in the OCA layer. **e** Force-displacement curves obtained from the interfacial shear tests for three samples. The maximum interfacial shear strength is identified as τ_{max} = 0.45 ± 0.02 MPa, which is a key parameter in the theoretical model of interfacial debonding limit (R_{1min}).

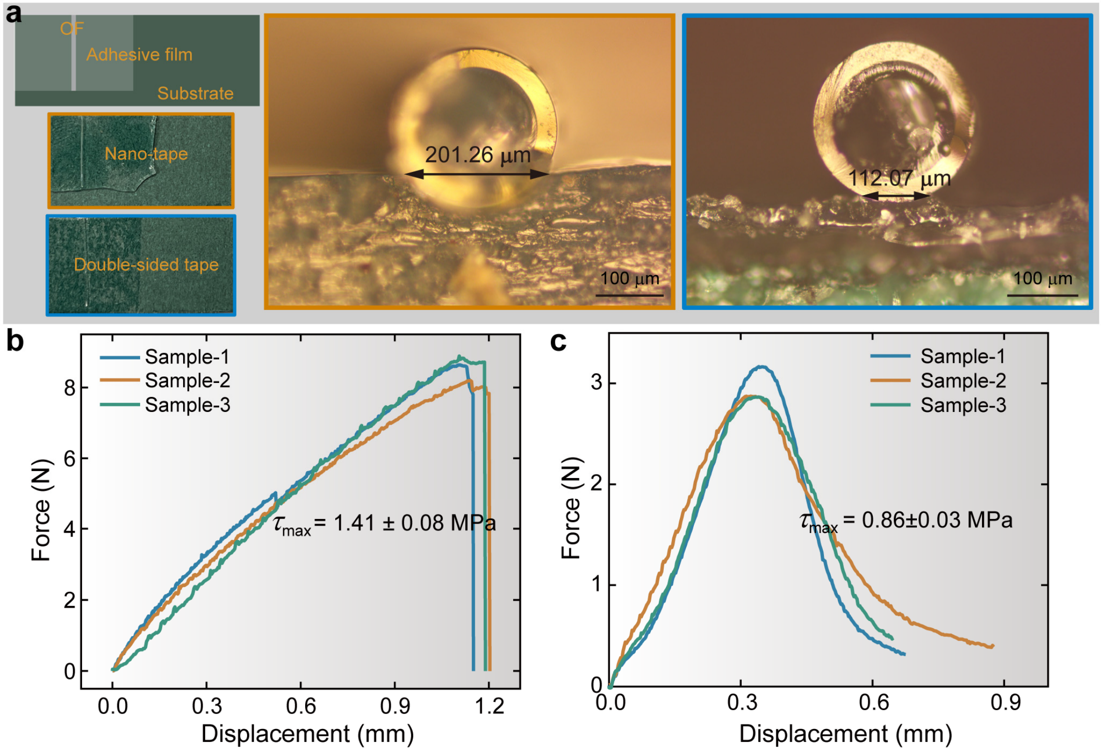

Supplementary Fig. 3 Interfacial adhesion characterization of the optical fiber (OF) on alternative adhesive substrates. a Optical micrographs and cross-sectional views of the OF partially embedded in nano-tape (orange frame) and double-sided tape (blue frame). The measured contact widths (b) for these substrates are b_{nano} = 201.26 μm and b_{double} =112.07 μm, respectively. **b** Force-displacement curves obtained from interfacial shear tests for the OF on nano-tape substrate. The average maximum interfacial shear strength is determined as τ_{max} = 1.41 ± 0.08 MPa. **c** Force-displacement curves for the OF on double-sided tape, yielding an average maximum interfacial shear strength of τ_{max} =0.86 ± 0.03 MPa. These experimental parameters serve as key inputs for validating the R_{1min} theoretical framework across diverse adhesive materials.

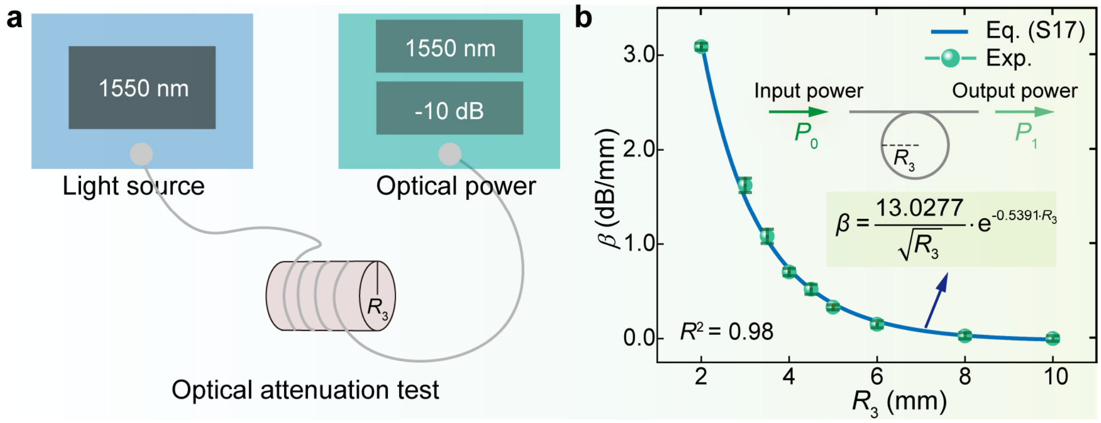

Supplementary Fig. 4 Experimental calibration of macrobending-induced optical attenuation. a Schematic illustration of the optical attenuation test setup. A light source operating at 1550 nm is coupled into an optical fiber (OF) wound around a cylinder with a predefined radius R_3, and the transmitted power is monitored using an optical power meter to quantify macrobending loss. **b** Attenuation coefficient β as a function of the bending radius. The experimental data (Exp., green circles) show a strong correlation ($R^2 = 0.98$) with the fitted theoretical model (Eq. (S17), blue solid line). The inset illustrates the power transmission model where P_0 and P_1 denote the input and output optical power, respectively.

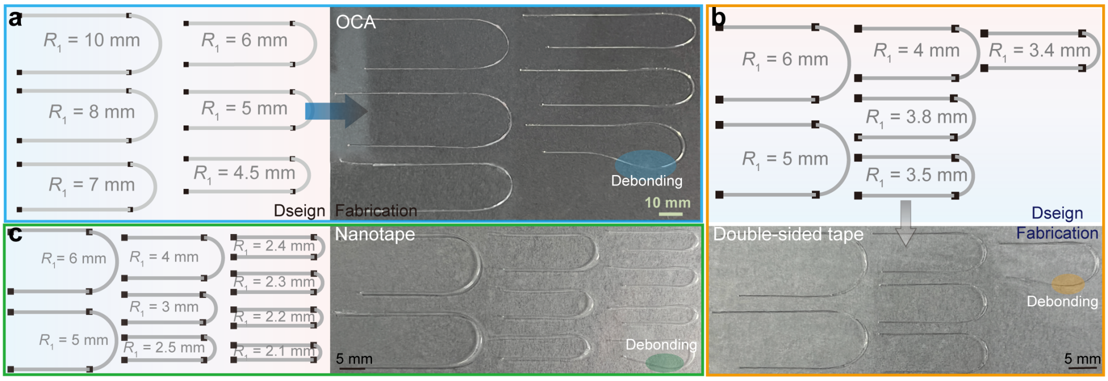

Supplementary Fig. 5 Experimental validation of the minimum in-plane bending radius (R_1) for interfacial stability on various adhesive substrates. a Comparison between digitally designed and fabricated optical fiber (OF) patterns on optically clear adhesive (OCA). The black squares in the design schematics represent the path points of the printing trajectory. Stable assembly is maintained for radii $R_1 \geq 5$ mm, while localized interfacial debonding (highlighted by the blue ellipse) occurs as R_1 decreases to 4.5 mm. **b** Validation of the bending limit on double-sided tape, where pattern fidelity is compromised by debonding at $R_1 = 3.4$ mm. **c** Patterning capability on nanotape across a wide range of radii. The superior interfacial shear strength of nanotape allows for stable routing down to $R_1 = 2.2$ mm, with mechanical detachment observed only at $R_1 = 2.1$ mm.

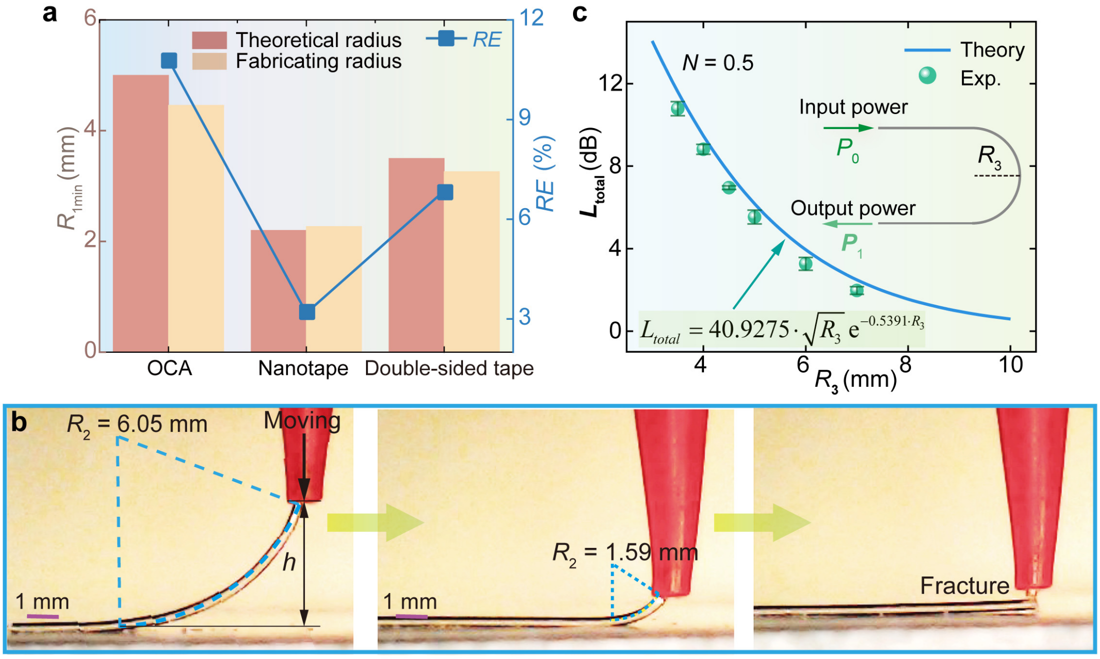

Supplementary Fig. 6 Theoretical validation and experimental verification of multi-scale geometric constraints for DFP. **a** Comparison between theoretical predictions and experimental measurements (fabricating radius) of the minimum in-plane bending radius (R_{1min}) across various substrates (OCA, nanotape, and double-sided tape). The relative error (*RE*) between the theoretical model and experimental data remains below 11%. **b** Sequential optical micrographs demonstrating the out-of-plane bending process and the critical brittle fracture of the optical fiber (OF). As the height *h* decreases, the bending radius R_2 reduces from 6.05 mm to the fracture limit (approximately 1.59 mm in this experiment). **c** Total macrobending loss (L_{total}) as a function of the bending radius R_3 for $N = 0.5$. The experimental data (green circles) exhibit excellent agreement with the theoretical curve (blue solid line). The inset illustrates the power transmission model where P_0 and P_1 denote the input and output optical power, respectively.

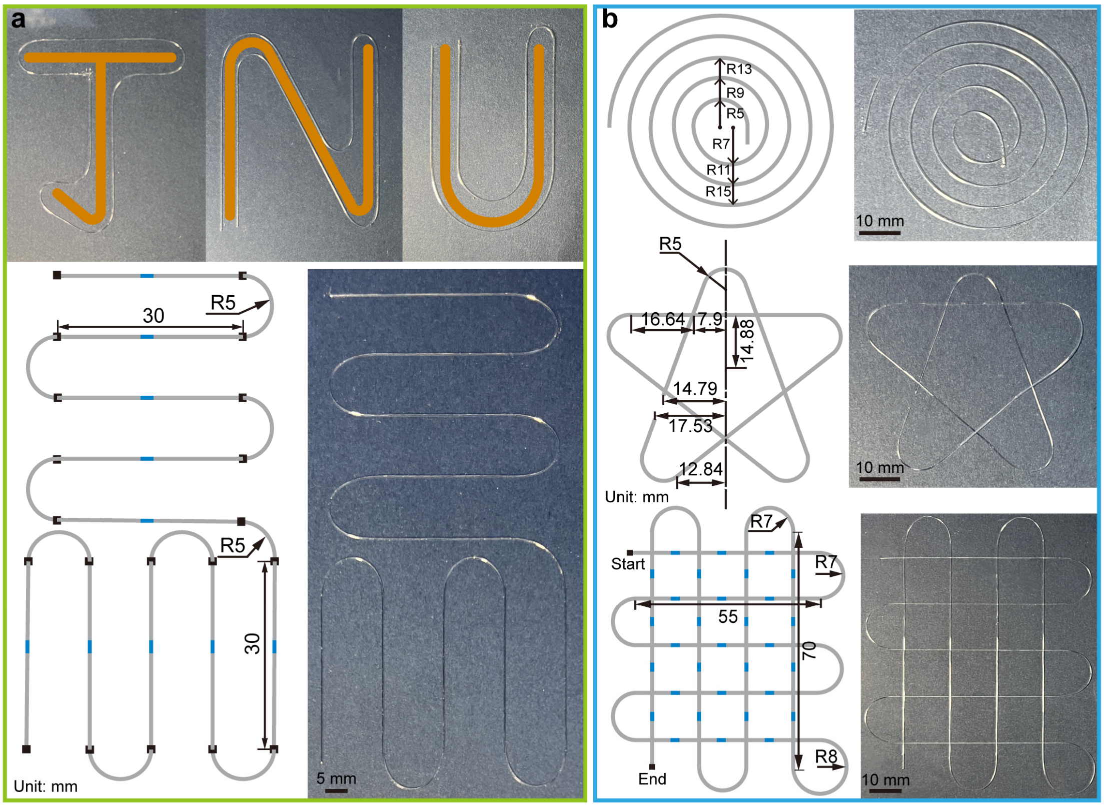

Supplementary Fig. 7 Demonstrations of complex geometric patterning and diverse path planning. a Patterning of alphabetic characters ("JNU", representing Jiangnan University) and large-area serpentine structures. The design schematics illustrate the discrete path points (black squares) and the strategically positioned Fiber Bragg Grating (FBG) sensor locations (blue segments) within the optical fiber (OF). **b** Diversified geometric assemblies including spiral, star-shaped, and high-density grid-like serpentine patterns. Specific bending radii (ranging from $R = 5$ to 15 mm) and path dimensions are labeled to demonstrate the high geometric fidelity and flexibility of the DFP process.

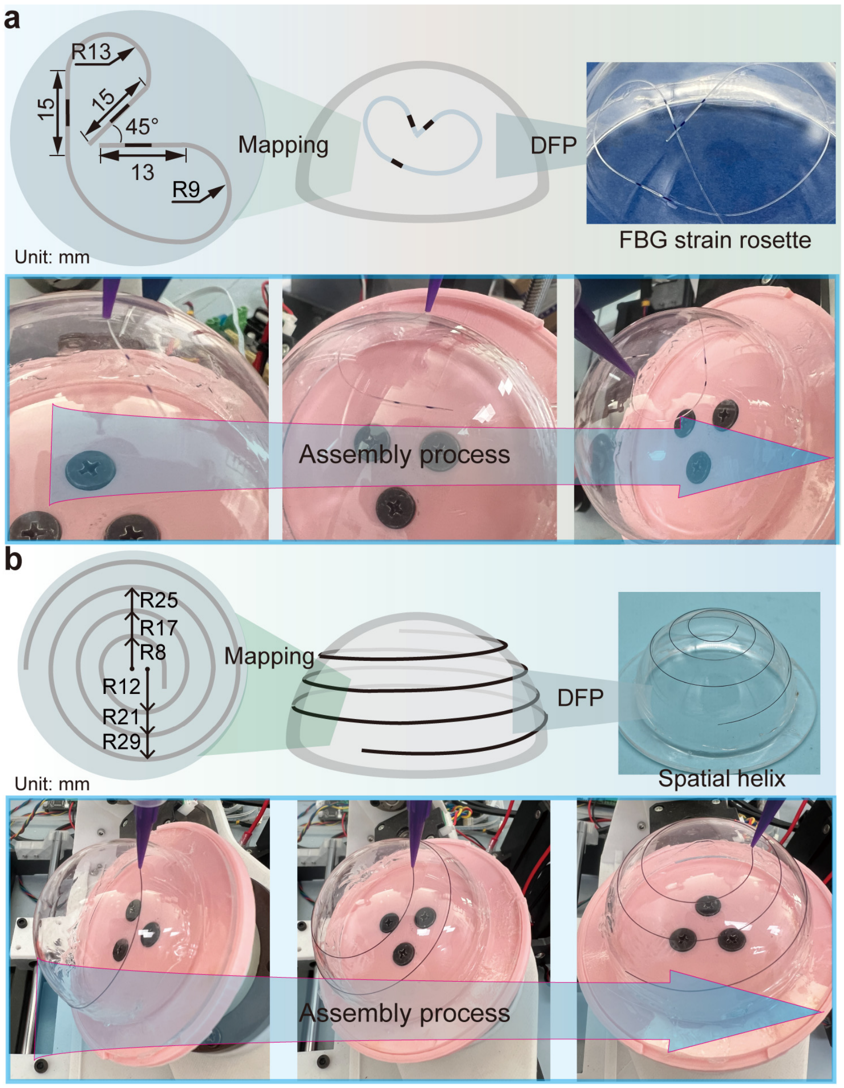

Supplementary Fig. 8 Conformal DFP assembly on hemispherical surfaces. a Implementation of a three-element Fiber Bragg Grating (FBG) strain rosette on a hemispherical substrate. The process involves mapping a 2D planar design onto the 3D spherical surface using a conformal mapping algorithm, followed by direct assembly on the physical substrate. Sequential photographs capture the dynamic assembly process, demonstrating high positional accuracy. **b** Fabrication of a spatial helix on a hemispherical geometry. A 2D spiral design with specified radii (*R* ranging from 8 to 29 mm) is mapped and assembled into a 3D spatial path. The experimental pictures illustrate the coordinated motion of the five-axis platform during the conformal patterning process.

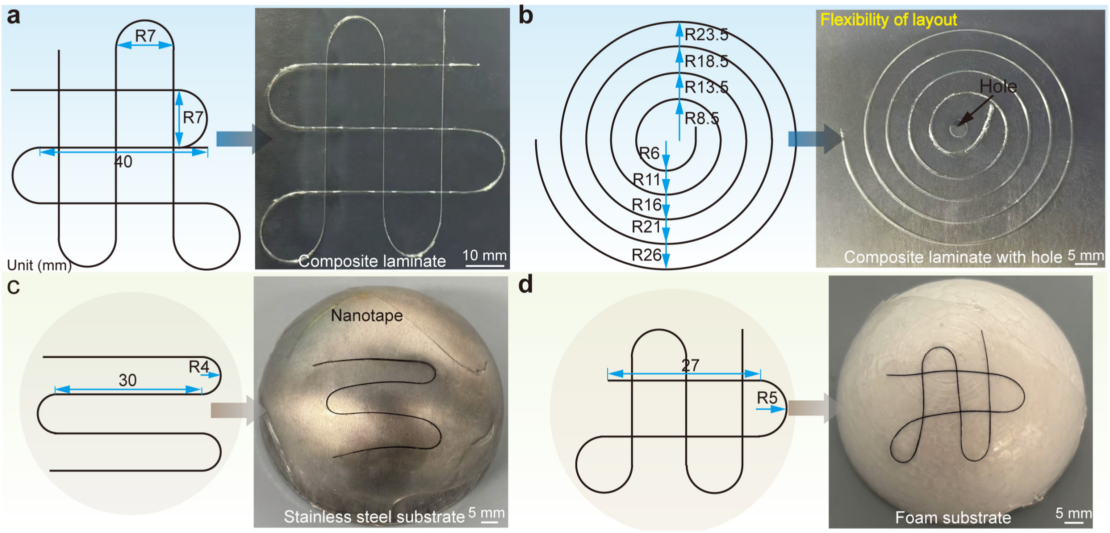

Supplementary Fig. 9 Versatility of direct-FBG-patterning (DFP) assembly on diverse engineering substrates. a Patterning of a serpentine structure on a flat composite laminate. The design schematic and fabricated sample show a bending radius $R = 7$ mm and a width of 40 mm. **b** Demonstration of layout flexibility on a composite laminate featuring a central hole. The spiral path (R ranging from 6 to 26 mm) is designed to bypass the obstacle, highlighting the adaptability of the DFP process for complex surface features. **c** Conformal assembly of a serpentine pattern on a hemispherical stainless-steel substrate. Nanotape is employed as the adhesive interface to ensure stable bonding on the metallic surface ($R = 4$ mm). **d** Complex routing on a foam substrate, demonstrating the capability of the five-axis platform to perform high-fidelity patterning on low-modulus and porous materials ($R = 5$ mm).

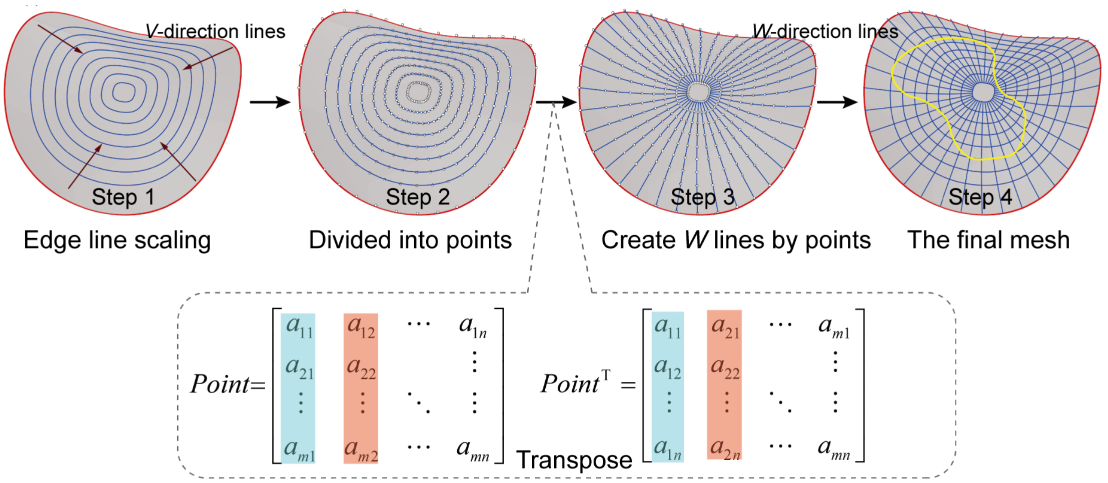

Supplementary Fig. 10 Workflow of the conformal mesh generation algorithm for complex path planning. Step 1, Generation of *V*-direction lines through edge line scaling based on the surface boundaries. Step 2, Discretization of the *V*-lines into a structured array of coordinate points. Step 3, Construction of *W*-direction lines by connecting corresponding points across the *V*-lines. This is facilitated by matrix transposition (illustrated in the dashed box), where the point coordinate matrix *point* reorganized into $point^{T}$ to enable path generation in the transverse direction. Step 4, Assembly of the *V* and *W* lines to form the final conformal mesh, which provides the geometric framework for direct-FBG-patterning (DFP) on complex curved surfaces.

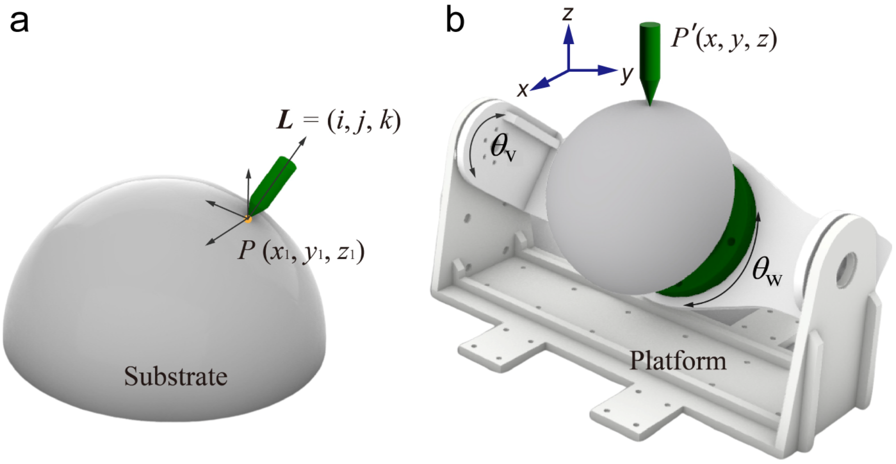

Supplementary Fig. 11 Kinematic model and coordinate transformation for five-axis coordinated direct-FBG-patterning (DFP). a The needle posture is represented in the workpiece coordinate system. **P** (x_1, y_1, z_1) defines the target spatial position on the curved substrate, and **L** = (i, j, k) represents the axis vector of the deposition needle, which is typically maintained perpendicular to the surface tangent plane. **b** Configuration of the machine coordinate system and the five-axis motion platform. The kinematic algorithm maps the needle position into machine-specific coordinates **P'**(x, y, z) and rotational angles (θ_V and θ_W), enabling synchronized motion for DFP on complex three-dimensional geometries.

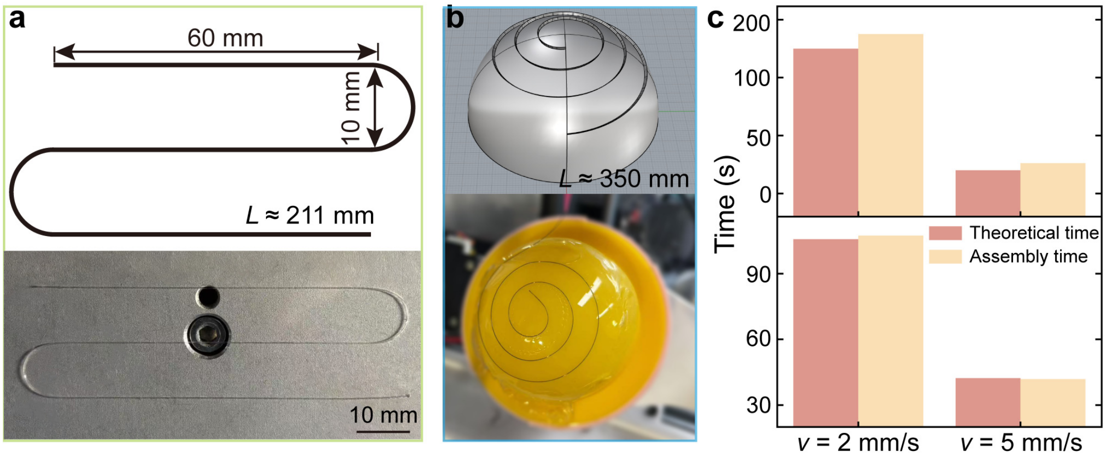

Supplementary Fig. 12 Validation of path length and assembly efficiency for direct-FBG-patterning (DFP). a Design schematic and fabricated sample of a serpentine pattern on a planar substrate, with a total path length of $L \approx 211$ mm. **b** 3D CAD model and conformal assembly of a spiral pattern on a hemispherical substrate ($L \approx 350$ mm). **c** Quantitative comparison between theoretical assembly time and actual assembly time at different printing velocities ($v = 2$ mm/s and $v = 5$ mm/s).

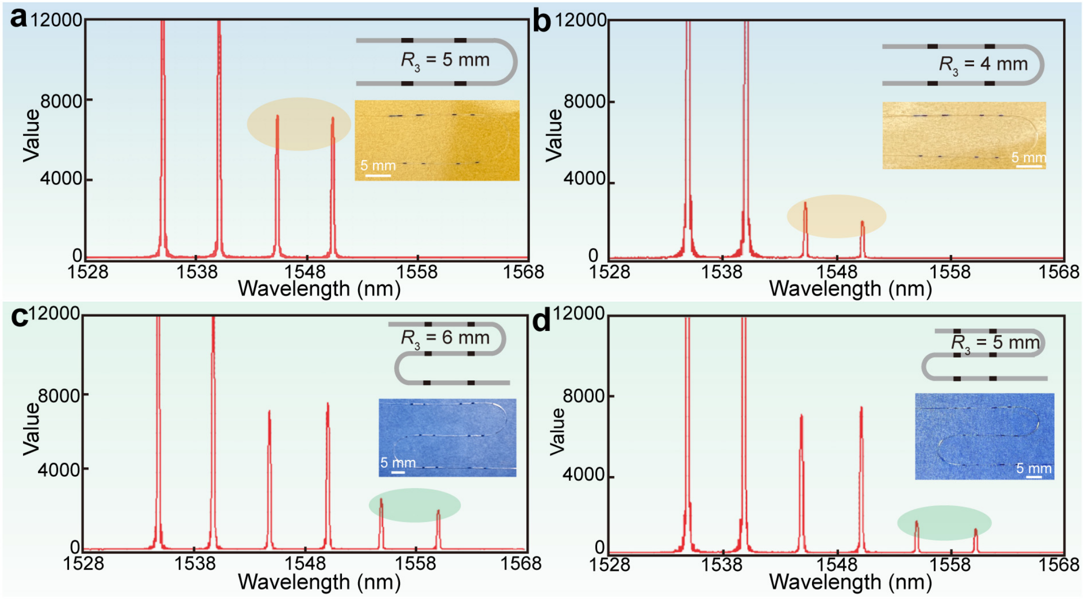

Supplementary Fig. 13 Influence of macrobending radius (R_3) on the optical signal integrity of direct-FBG-patterning (DFP) sensor arrays. a, **b** Optical reflection spectra and fabricated samples of FBG arrays with bending radii of R_3 = 5 mm and R_3 = 4 mm. The highlighted peaks in the yellow ellipses show significant power attenuation as R_3 decreases, due to the increased macrobending loss. **c**, **d** Spectra and samples for serpentine layouts with radii of R_3 = 6 mm and R_3 = 5 mm. The green ellipses mark the attenuated peaks. These results demonstrate that while DFP enables flexible routing, the bending radius must be maintained above the R_{3min} threshold to ensure a sufficient signal-to-noise ratio for multi-point sensing.

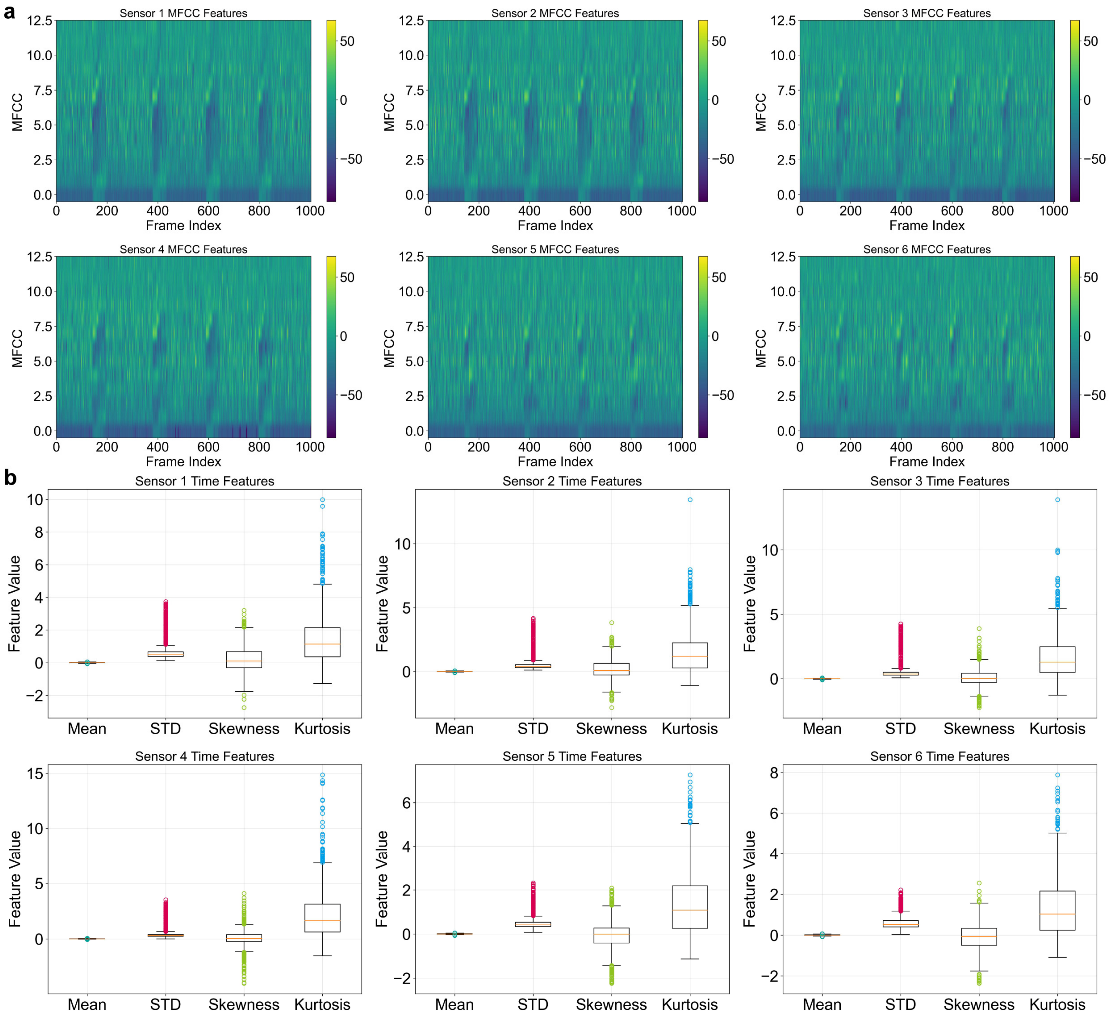

Supplementary Fig. 14 Feature extraction and statistical distribution of the FBG sensor array signals. a Mel-frequency cepstral coefficients (MFCC) feature maps extracted from the responsive signals of six sensors (FBG Sensors 1-6). The heatmaps visualize the spectral evolution across frame indices. **b** Time-domain statistical features for each sensor, including Mean, Standard Deviation (STD), Skewness, and Kurtosis. The box plots characterize the distribution and variability of the raw signal data, where the central lines indicate the medians, and the individual points represent data samples across multiple experiments.

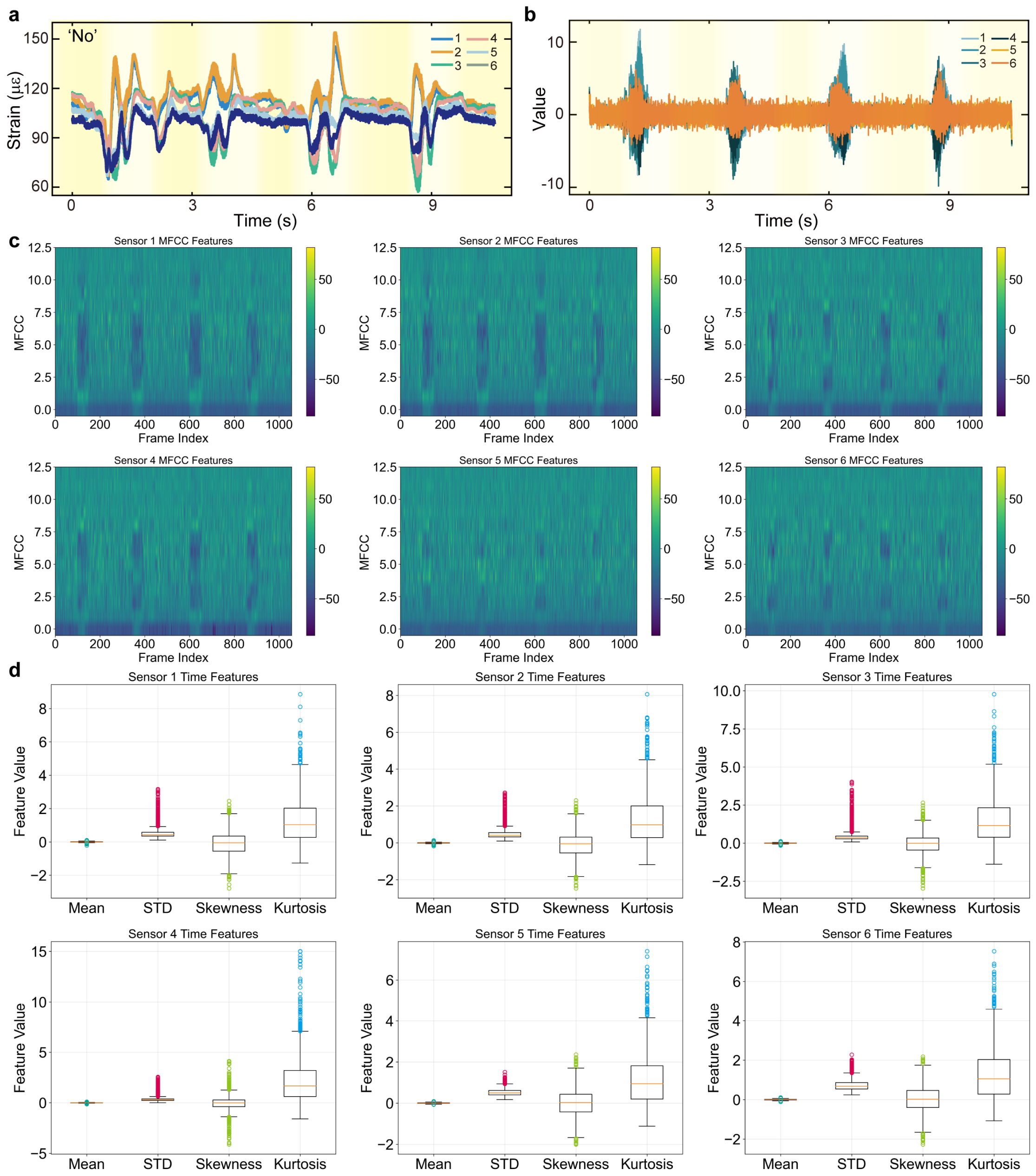

Supplementary Fig. 15 Signal characterization and multi-domain feature extraction for the pronunciation of the word 'No'. a Representative raw strain responsive signals acquired from the six-sensor Fiber Bragg Grating (FBG) array during the vocalization of the English word 'No'. The sensors, assembled via direct-FBG-patterning (DFP), detect subtle mechanical strains induced by laryngeal vibrations and associated muscle movements. **b** Pre-processed signals after baseline drift removal and normalization, highlighting the distinct temporal signatures and power distribution unique to the 'No' phonetic input. **c** Two-dimensional feature maps generated via Mel-frequency cepstral coefficients (MFCC) extraction for each sensor channel, providing

a high-dimensional spectral-temporal representation of the speech-induced vibrations. **d** Statistical analysis of time-domain features, including Mean, Standard Deviation (STD), Skewness, and Kurtosis. The box plots visualize the distribution and stability of features across multiple experiments.

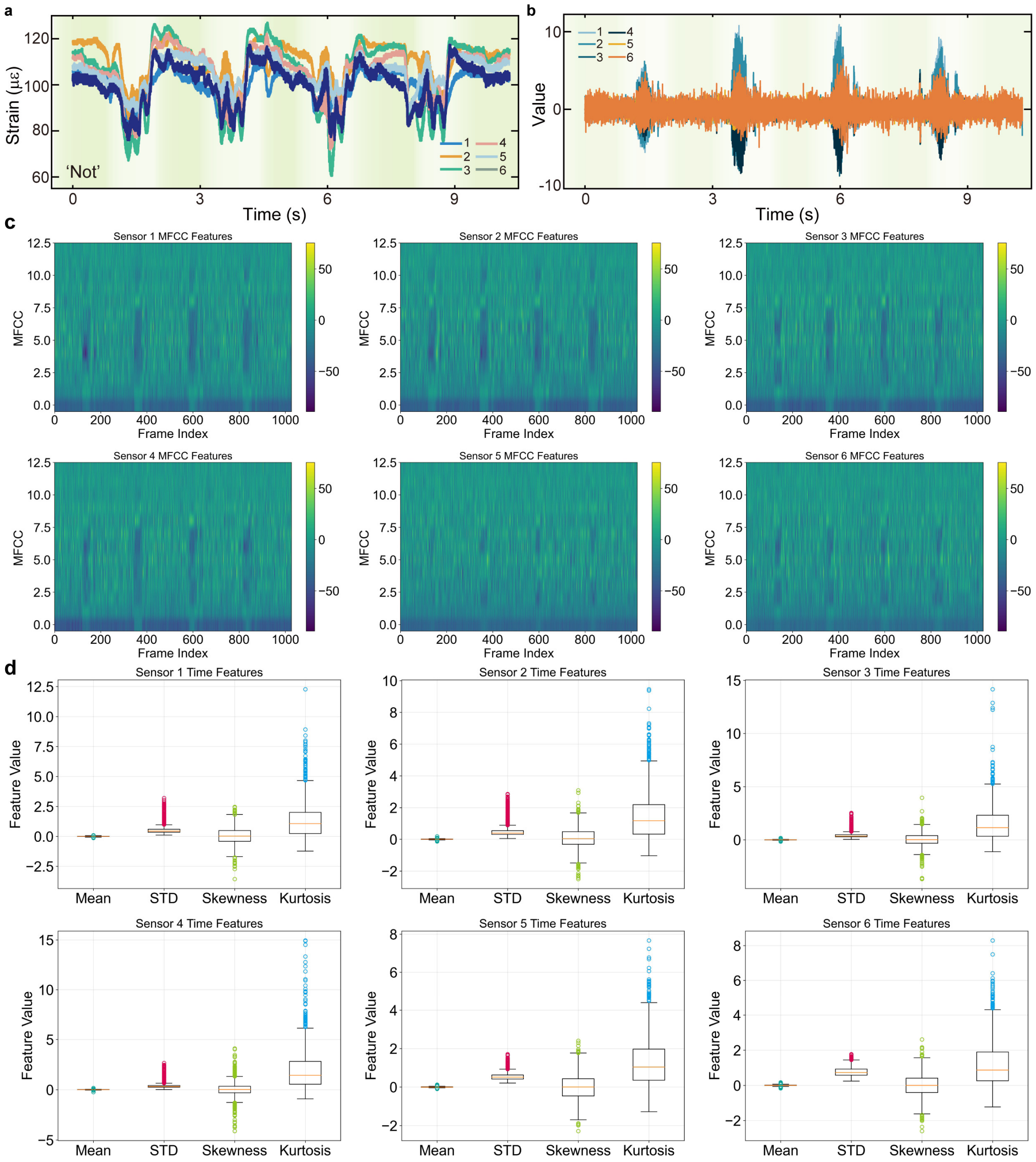

Supplementary Fig. 16 Signal characterization and multi-domain feature extraction for the pronunciation of the word 'Not'. a Representative raw strain responsive signals acquired from the six-sensor Fiber Bragg Grating (FBG) array during the vocalization of the English word 'Not'. The sensors, assembled via direct-FBG-patterning (DFP), detect subtle mechanical strains induced by laryngeal vibrations and associated muscle movements. **b** Pre-processed signals after baseline drift removal and normalization, highlighting the distinct temporal signatures and power distribution unique to the 'Not' phonetic input. **c** Two-dimensional feature maps generated via Mel-frequency cepstral coefficients (MFCC) extraction for each sensor channel, providing

a high-dimensional spectral-temporal representation of the speech-induced vibrations. **d** Statistical analysis of time-domain features, including Mean, Standard Deviation (STD), Skewness, and Kurtosis. The box plots visualize the distribution and stability of features across multiple experiments.

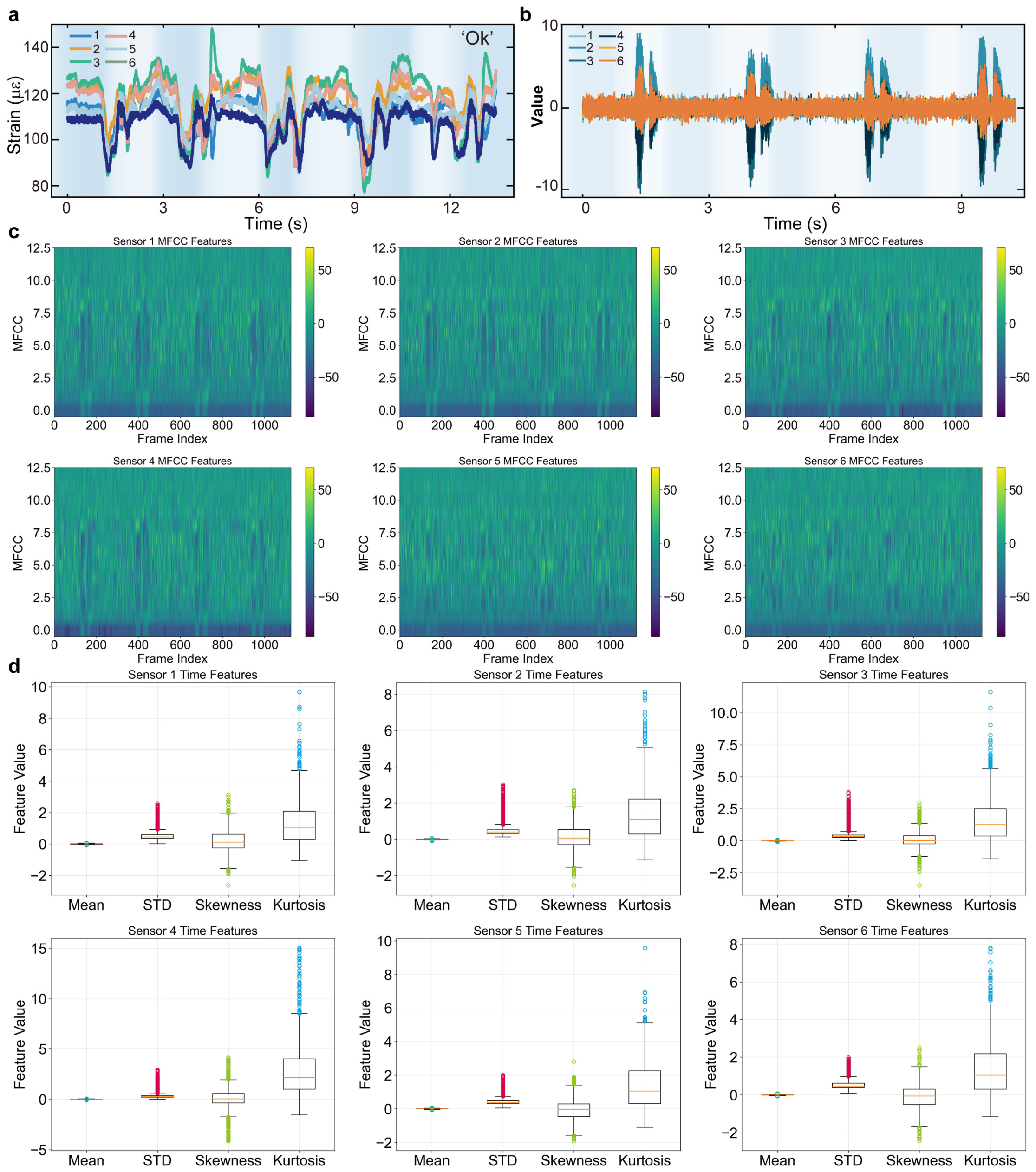

Supplementary Fig. 17 Signal characterization and multi-domain feature extraction for the pronunciation of the word 'Ok'. a Representative raw strain responsive signals acquired from the six-sensor Fiber Bragg Grating (FBG) array during the vocalization of the English word 'Ok'. The sensors, assembled via direct-FBG-patterning (DFP), detect subtle mechanical strains induced by laryngeal vibrations and associated muscle movements. **b** Pre-processed signals after baseline drift removal and normalization, highlighting the distinct temporal signatures and power distribution unique to the 'Ok' phonetic input. **c** Two-dimensional feature maps generated via Mel-frequency cepstral coefficients (MFCC) extraction for each sensor channel, providing

a high-dimensional spectral-temporal representation of the speech-induced vibrations. **d** Statistical analysis of time-domain features, including Mean, Standard Deviation (STD), Skewness, and Kurtosis. The box plots visualize the distribution and stability of features across multiple experiments.

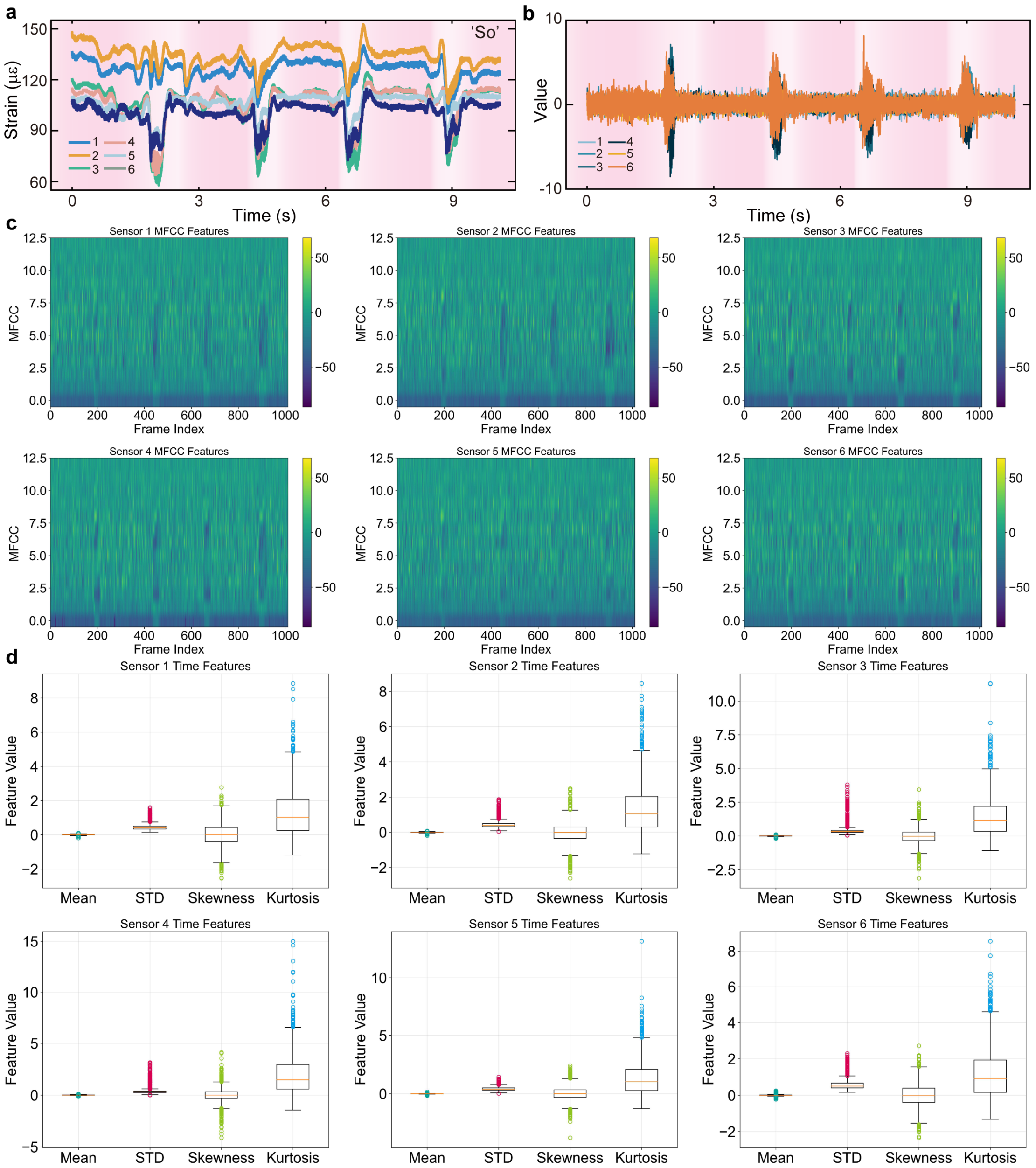

Supplementary Fig. 18 Signal characterization and multi-domain feature extraction for the pronunciation of the word 'So'. a Representative raw strain responsive signals acquired from the six-sensor Fiber Bragg Grating (FBG) array during the vocalization of the English word 'So'. The sensors, assembled via direct-FBG-patterning (DFP), detect subtle mechanical strains induced by laryngeal vibrations and associated muscle movements. **b** Pre-processed signals after baseline drift removal and normalization, highlighting the distinct temporal signatures and power distribution unique to the 'So' phonetic input. **c** Two-dimensional feature maps generated via Mel-frequency cepstral coefficients (MFCC) extraction for each sensor channel, providing

a high-dimensional spectral-temporal representation of the speech-induced vibrations. **d** Statistical analysis of time-domain features, including Mean, Standard Deviation (STD), Skewness, and Kurtosis. The box plots visualize the distribution and stability of features across multiple experiments.

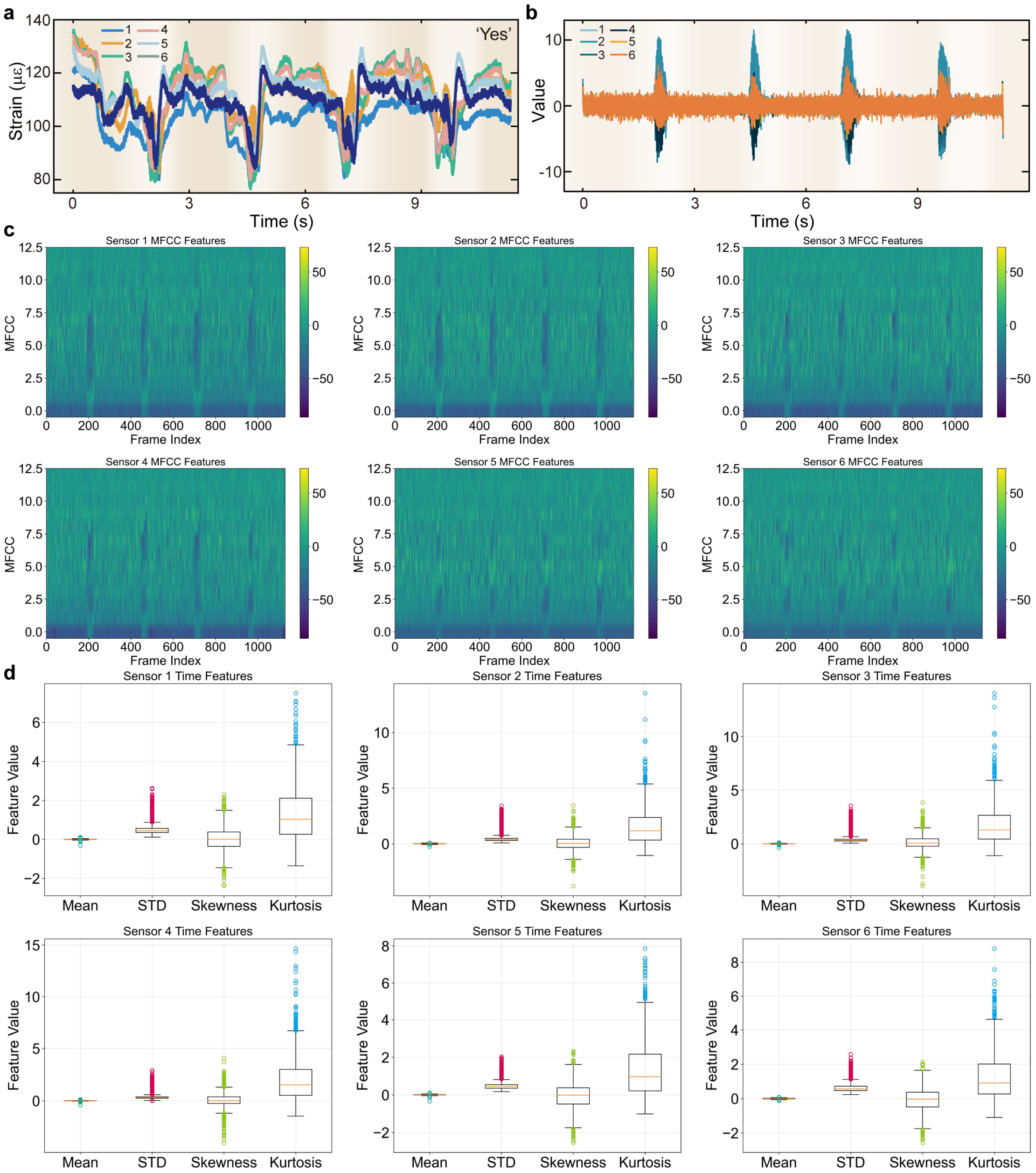

Supplementary Fig. 19 Signal characterization and multi-domain feature extraction for the pronunciation of the word 'Yes'. a Representative raw strain responsive signals acquired from the six-sensor Fiber Bragg Grating (FBG) array during the vocalization of the English word 'Yes'. The sensors, assembled via direct-FBG-patterning (DFP), detect subtle mechanical strains induced by laryngeal vibrations and associated muscle movements. **b** Pre-processed signals after baseline drift removal and normalization, highlighting the distinct temporal signatures and power distribution unique to the 'Yes' phonetic input. **c** Two-dimensional feature maps generated via Mel-frequency cepstral coefficients (MFCC) extraction for each sensor channel, providing

a high-dimensional spectral-temporal representation of the speech-induced vibrations. **d** Statistical analysis of time-domain features, including Mean, Standard Deviation (STD), Skewness, and Kurtosis. The box plots visualize the distribution and stability of features across multiple experiments.

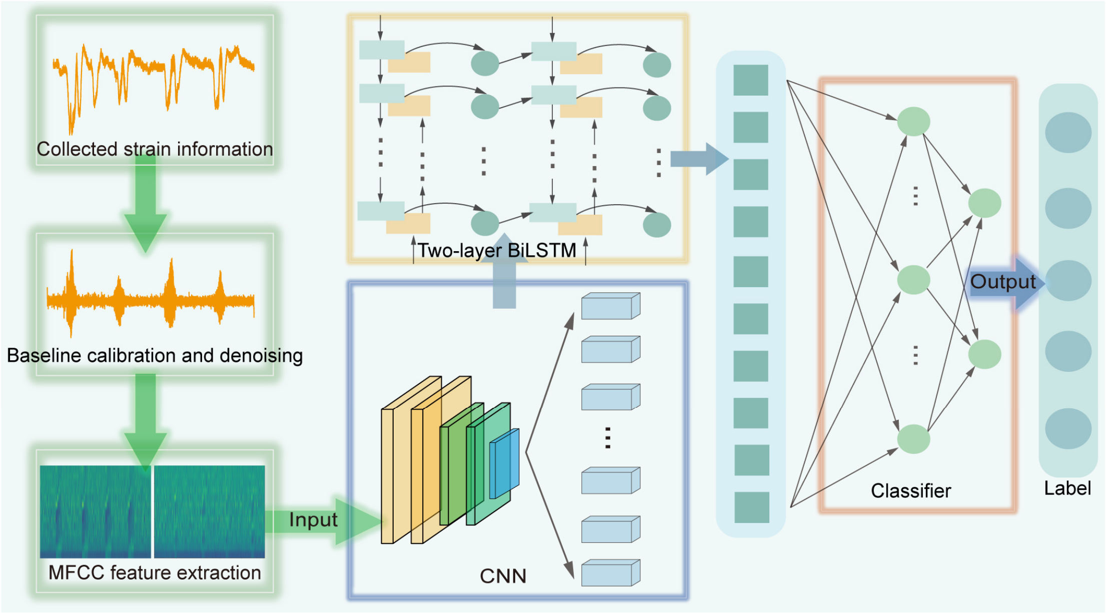

Supplementary Fig. 20 Deep learning architecture for vibration-based speech recognition. The hierarchical framework illustrates the end-to-end process of translating mechanical strain signals into phonetic labels. Raw strain information, acquired via the Fiber Bragg Grating (FBG) sensor array, first undergoes baseline calibration and denoising to remove environmental interference. Mel-frequency cepstral coefficients (MFCC) are then extracted to transform the one-dimensional temporal signals into two-dimensional spectral-temporal feature maps. These maps are processed by a Convolutional Neural Network (CNN) for spatial feature learning, followed by a two-layer Bidirectional Long Short-Term Memory (BiLSTM) network designed to capture the complex, long-term temporal dependencies inherent in human vocalization. A final classifier maps the extracted features to specific output labels, enabling high-accuracy speech recognition through the direct-FBG-patterning (DFP)-integrated sensing system.

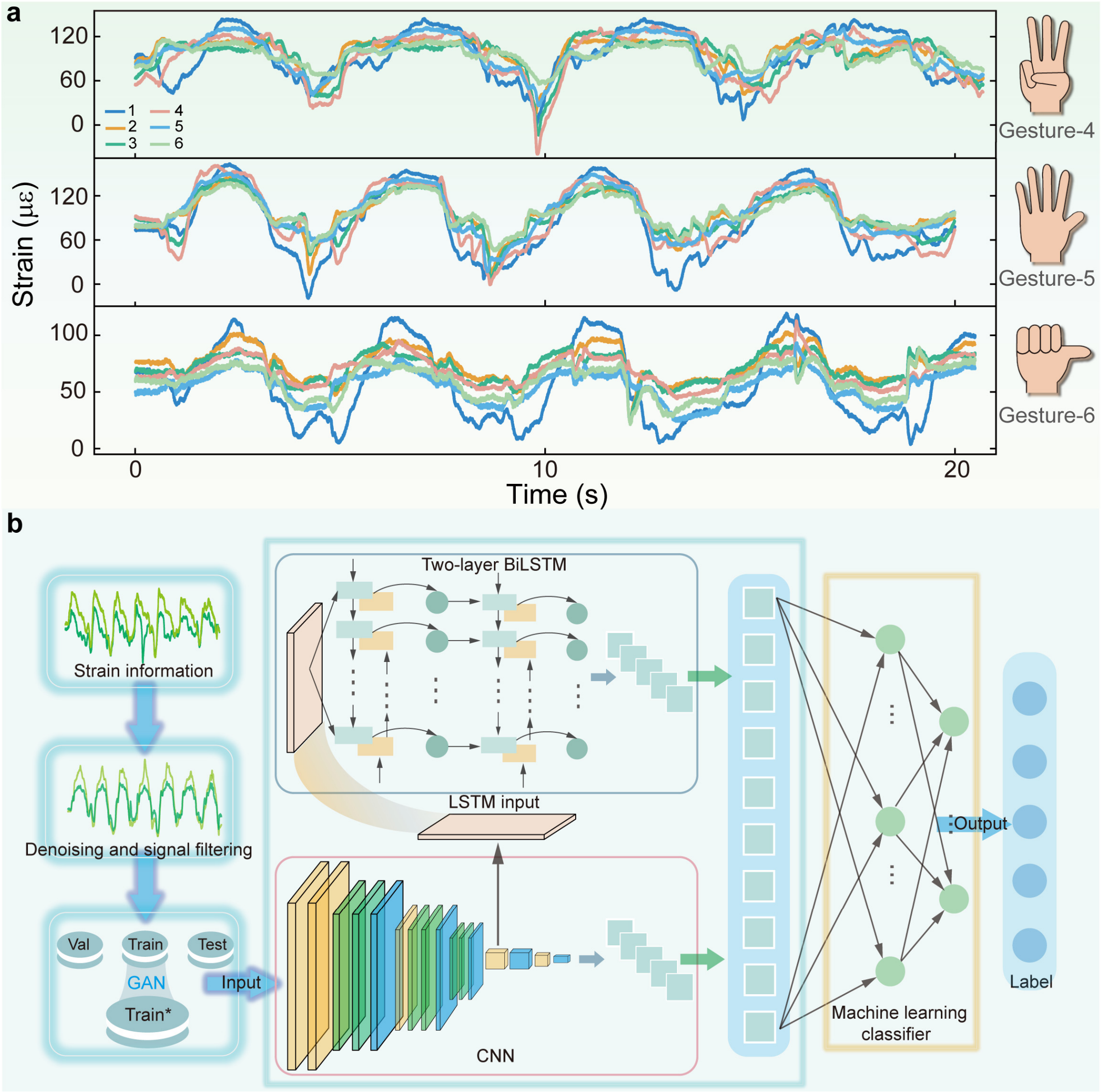

Supplementary Fig. 21 Intelligent gesture recognition framework and signal characterization. a Dynamic strain response acquired from the six-sensor Fiber Bragg Grating (FBG) array for representative hand gestures (Gestures 4-6). The temporal signatures reflect distinct muscle contraction patterns and joint movements captured by the sensors assembled via direct-FBG-patterning (DFP). **b** Schematic of the gesture recognition pipeline. Following baseline denoising and filtering, a Generative Adversarial Network (GAN) is employed for data augmentation to expand the training dataset (Train*). The hybrid architecture utilizes a Convolutional Neural Network (CNN) for spatial feature extraction and a two-layer Bidirectional Long Short-Term Memory (BiLSTM) network for temporal dependency modeling. A machine learning classifier finally maps the fused features to specific gesture labels.

Supplementary Movie 1 Real-time gesture recognition using the DFP-assembled FBG sensor array and deep learning. This video demonstrates the end-to-end performance of the integrated sensing system for real-time human-machine interaction. A flexible Fiber Bragg Grating (FBG) sensor array, integrated via the direct-FBG-patterning (DFP) technique, is conformally attached to the wrist to capture high-fidelity strain signals during different hand gestures. The video showcases the synchronous acquisition of these multi-channel signals and their instantaneous classification into specific gesture labels (Gestures 1-6) by a trained model. The high recognition accuracy in the video highlights the system's potential for robust wearable sensing.

Supplementary References:

1. Faustini, L. & Martini, G. Bend loss in single-mode fibers. *J. Lightwave Technol.* **15**, 671-679 (1997).
2. Wang, Q., Farrell, G. & Freir, T. Theoretical and experimental investigations of macro-bend losses for standard single mode fibers. *Opt. Express* **13**, 4476-4484 (2005).
3. Li, S. S. *et al.* AC electrokinetics-enhanced capacitive immunosensor for point-of-care serodiagnosis of infectious diseases. *Biosens. Bioelectron.* **51**, 437-443 (2014).
4. Severin, I., El Abdi, R. & Poulain, M. Strength measurements of silica optical fibers under severe environment. *Opt. Laser Technol.* **39**, 435-441 (2007).
5. Adams, R. D. Prediction of the strength of adhesive lap joints. An investigative review. *Int. J. Adhes. Adhes.* **129**, 103576 (2024).
6. Singamneni, S., Roychoudhury, A., Diegel, O. & Huang, B. Modeling and evaluation of curved layer fused deposition. *J. Mater. Process. Technol.* **212**, 27-35 (2012).
7. Isa, M. A. & Lazoglu, I. Five-axis additive manufacturing of freeform models through buildup of transition layers. *J. Manuf. Syst.* **50**, 69-80 (2019).
8. Hong, F. *et al.* 5-axis multi-material 3D printing of curved electrical traces. *Addit. Manuf.* **70**, 103546 (2023).
9. You, R., Ren, L. & Song, G. A novel fiber Bragg grating (FBG) soil strain sensor. *Measurement* **139**, 85-91 (2019).
10. Wang, H.-P. *et al.* Computer-aided feature recognition of CFRP plates based on real-time strain fields reflected from FBG measured signals. *Compos. Part B Eng.* **263**, 110866 (2023).
11. Li, Y., Yang, K. & Li, X. Temperature sensing characteristics of metal coated FBG during dynamic cooling process. *Opt. Fiber Technol.* **45**, 368-375 (2018).
12. Bhaskar, C. V. N., Pal, S. & Pattnaik, P. K. Recent advancements in fiber Bragg gratings based temperature and strain measurement. *Results Opt.* **5**, 100130 (2021).
13. Yassin, M. H., Farhat, M. H., Soleimanpour, R. & Nahas, M. Fiber Bragg grating (FBG)-based sensors: a review of technology and recent applications in structural health monitoring (SHM) of civil engineering structures. *Discov. Civil Eng.* **1**, 14 (2024).
14. Tao, Y. *et al.* Displacement-reconstruction-realized components by structure-sensing integration via a hybrid 3D printing strategy. *Compos. Part A Appl. Sci. Manuf.* **177**, 107896 (2024).
15. Guo, M. H. *et al.* Attention mechanisms in computer vision: a survey. *Comput. Vis. Media* **8**, 331-368 (2022).
16. Kim, K. K. *et al.* A deep-learned skin sensor decoding the epicentral human motions. *Nat. Commun.* **11**, 2149 (2020).
