# Supplementary material for: Programmable direct-patterning assembly enables high-density and surface-conformal integration of fiber Bragg grating sensor arrays
Source: Nat Commun. 2026 May 4;17:5998. doi: 10.1038/s41467-026-72613-3 (PMC13346946; doi:10.1038/s41467-026-72613-3)
Supplement: Supplementary file 2 — Description of Additional Supplementary File [file 41467_2026_72613_MOESM2_ESM.pdf]

Description of Additional summary files

Supplementary Movie 1:

Real-time gesture recognition using the DFP-assembled FBG sensor array and deep learning. This video demonstrates the end-to-end performance of the integrated sensing system for real-time human-machine interaction. A flexible Fiber Bragg Grating (FBG) sensor array, integrated via the direct-FBG-patterning (DFP) technique, is conformally attached to the wrist to capture high-fidelity strain signals during different hand gestures. The video showcases the synchronous acquisition of these multi-channel signals and their instantaneous classification into specific gesture labels (Gestures 1-6) by a trained model. The high recognition accuracy in the video highlights the system's potential for robust wearable sensing.
