# Supplementary material for: Programmable direct-patterning assembly enables high-density and surface-conformal integration of fiber Bragg grating sensor arrays
Source: Nat Commun. 2026 May 4;17:5998. doi: 10.1038/s41467-026-72613-3 (PMC13346946; doi:10.1038/s41467-026-72613-3)
Supplement: Supplementary file 4 — Transparent Peer Review file [file 41467_2026_72613_MOESM4_ESM.pdf]

nature portfolio

Peer Review File

Programmable direct-patterning assembly enables high-density and surface-conformal integration of fiber Bragg grating sensor arrays

Corresponding Author: Professor Junhua Zhao

This file contains all reviewer reports in order by version, followed by all author rebuttals in order by version.

Version 0:

Reviewer comments:

Reviewer #1

(Remarks to the Author)
Summary
The manuscript claims the development of a "direct-FBG-writing (DFW)" method enabling programmable, high-precision, one-step fabrication of monofil FBG sensors on planar and curved surfaces, with applications in SHM and human-activity monitoring. While the work presents interesting demonstrations of patterning optical fibers on adhesive films and surfaces, the manuscript fundamentally misrepresents its technical contribution. In its current form, it lacks scientific clarity, contains major conceptual inconsistencies about FBG fabrication, and does not provide the essential optical or material characterization required for any paper claiming advances in FBG sensor technology.
Major Concerns
1. The manuscript is framed entirely around FBGs, but no actual FBG fabrication method is described
The title, abstract, and introduction repeatedly claim "direct writing of FBGs" using a novel "DFW" technique. However:
• Nowhere does the manuscript explain how the FBGs are inscribed in the fiber.
• There is no description of:
o The inscription method (UV writing? fs-laser? phase mask? point-by-point?).
o The refractive-index modulation mechanism.
o Photosensitivity treatment (hydrogen loading, Ge-doped fiber, etc.).
o Equipment, parameters, or setup.
• Actual FBGs only appear suddenly in Section 3 and 4 without any fabrication explanation.
Instead, the paper describes a method for mechanically placing and shaping pre-existing fibers/wires on adhesive films, not writing FBGs. For most of the paper, the authors use plain optical fibers as substitutes for FBGs (explicitly stated on page 7: "the OF was initially used as a substitute for FBG"). This contradicts the entire premise of "direct FBG writing".
2. What is the "optical fiber" the authors are printing? Is it a waveguide fabricated on the substrate? A real fiber? A polymer structure?
The manuscript repeatedly refers to the patterned material as "OF (optical fiber)", but this raises multiple fundamental questions:
• Are the authors printing a material that functions as an optical waveguide?
• If yes, what material is this? What are its refractive-index properties? Mode field? Loss?
• How can FBGs be inscribed if the waveguide is created by deposition and not drawn as a fiber?
• If the authors are not printing the waveguide but simply placing a commercial fiber (e.g., SMF-28e+), then:
o Why is this called "direct writing of FBGs"?
o Why is it called "monofil optical fiber"?
o Why is there no characterization of the mechanical and optical impact of adhesives used?
3. No characterization of the FBGs: optical spectra, Bragg wavelength, sensitivity, losses
A paper claiming to produce FBG sensors must provide:
• Reflection spectra
• Peak wavelength and bandwidth
• Sidelobes
• Reflectivity
• Strain and temperature coefficients

• Linearity, cross-sensitivity, hysteresis
None of these metrics appear anywhere in the manuscript.
Section 3–4 uses FBG arrays, but:
• No spectral data are properly shown.
• No proof of grating functionality beyond assuming a strain–wavelength coefficient (0.0012 nm/με).
• No evidence that the gratings even exist in the fabricated fibers.
This is unacceptable for a scientific paper about FBG fabrication.
4. The manuscript describes only mechanical placement of fibers using adhesives, not FBG fabrication
Most of the paper focuses on:
• Bending-radius limits
• Adhesion mechanics
• Using nanotape or OCA
• Spatial positioning of pre-made fibers
• Routing fibers on 2D/3D surfaces
This is essentially a fiber-placement / assembly technology, not an FBG-writing technology.
The term “direct FBG writing” is therefore misleading. The novelty appears to be a robotic fiber-placement system, not an optical fabrication method.
5. No scientific novelty, primarily engineering assembly / applied demonstrations
The paper presents interesting demonstrations:
• Patterned fiber routing
• Printing on curved surfaces
• Gesture and voice recognition using pre-existing FBG sensors
However:
• These do not constitute scientific advances in fiber optics.
• The “methods” are mechanical or procedural, not optical or material science.
• The use cases (SHM, human activity recognition) are straightforward applications of standard FBGs.
The lack of scientific depth makes the manuscript unsuitable for Nature Communications.
6. Several contradictions and unclear statements
Examples:
• DFW is described as a method to “directly write FBGs”, but actual FBGs are written earlier via UV exposure and then manually assembled using the DFW method.
• The authors call OF patterns “functional FBGs” even when grating regions are not written.
These internal contradictions severely damage clarity.
Recommendation
I cannot recommend this manuscript for publication in Nature Communications in its current form.
The core issue is that the manuscript presents itself as a breakthrough in FBG fabrication, but does not actually fabricate FBGs using the claimed method, nor provide any optical or material characterization expected for such work.
The contribution appears to be an applied robotic fiber-placement technique, not a scientific advance in photonics.
Given these major conceptual and scientific deficiencies, the manuscript requires major revision at minimum, and may not be suitable for this journal even after a significant revision.

Reviewer #2

(Remarks to the Author)
see attached file

Reviewer #3

(Remarks to the Author)
Overall Evaluation

This manuscript presents a comprehensive and technically solid study on the direct-FBG-writing (DFW) method for fabricating programmable, high-precision monofil fiber Bragg grating (FBG) sensors on curved surfaces. The work demonstrates high novelty by achieving one-step conformal manufacturing of FBG arrays with extreme unit density and complex geometries, overcoming limitations of traditional manual FBG assembly methods. The experimental validation, theoretical modeling, and deep-learning-driven applications (speech and gesture recognition) are all of high interest to the optical sensing, additive manufacturing, and intelligent systems communities.

1. Noteworthy Results

The paper introduces a one-step DFW method capable of fabricating high-density, conformal FBG sensor arrays on both planar and curved substrates.

The authors developed and experimentally validated three minimum bending radius models (R_1min, R_2min, R_3min) addressing fiber debonding, fracture, and optical degradation, respectively. This provides a robust theoretical foundation for DFW parameter optimization.

Demonstrations include:

Torsional displacement reconstruction on wing-like structures for structural health monitoring (SHM).

Phonation monitoring and gesture recognition using FBG arrays integrated with deep learning (MFCC–CRNN–LSTM architectures), achieving >95% accuracy.

The integration of mechanical modeling, fabrication, and AI-driven sensing demonstrates strong interdisciplinary innovation.

2. Significance and Comparison to Literature

The work is of high significance for the advancement of FBG sensor integration and intelligent sensing technologies:

Compared with traditional adhesive bonding methods [13–18], the DFW approach enables programmable, conformal, and large-area integration with enhanced precision and reproducibility.

Previous reports (e.g., Guo et al., Composites Science and Technology, 2020; Lu et al., Soft Robotics, 2024) focused on flexible electronic or hybrid printed sensors, but none demonstrated direct optical fiber patterning with this level of mechanical and optical integrity.

The proposed DFW platform bridges additive manufacturing and photonics, offering a route toward scalable smart-surface fabrication.

The paper is well-positioned relative to state-of-the-art references in Nature Communications, Advanced Science, and IEEE Sensors Journal, showing clear original contribution.

3. Validity of Data and Conclusions

The data analysis and interpretation are coherent and well supported by both theoretical and experimental results:

The theoretical models of minimum bending radii are validated by empirical measurements with strong quantitative agreement (Table 2).

Strain and displacement reconstruction experiments (Fig. 4) show consistent results with finite element analysis.

Machine-learning-based phonation and gesture recognition results are statistically well presented, with clear confusion matrices and accuracy curves (Figs. 5–6).

However, two aspects could be improved:

Statistical robustness – The number of repetitions for sensor calibration and strain tests should be specified to assess variability.

Uncertainty quantification – Reporting standard deviations or error bars in optical attenuation and strain measurement data would enhance confidence in reproducibility.

4. Methodology and Reproducibility

The methodology is technically rigorous and well described. The use of continuum mechanics modeling, multi-axis DFW control, and deep learning for signal interpretation is appropriate and innovative.
Still, for full reproducibility:

The parameters for FBG laser inscription, adhesive curing conditions, and exact path-planning algorithm used in the 5-axis printing platform should be described in greater detail (perhaps in the Supplementary Material).

The training dataset size and augmentation details for the speech and gesture recognition models should also be provided for replication.

5. Potential Flaws and Suggestions

Model limitations: The models assume isotropic adhesive and uniform fiber stress distribution; non-uniform interfacial properties in nanotape could affect local debonding. Discussion on these simplifications would strengthen the analysis.

Temperature compensation: Although tests are at 26 °C, real-world applications may face temperature variations. Incorporating a compensation model or noting the limitation would be valuable.

Broader validation: Demonstrating DFW fabrication on non-polymeric substrates (e.g., metals or composites) could show wider applicability.

6. Recommendation

The manuscript presents a substantial and original contribution to optical sensor manufacturing and intelligent system integration. The methodology is sound, data interpretation is convincing, and the results are of broad interdisciplinary impact.

I recommend minor revisions before publication to address the following:

Provide statistical uncertainty or error estimates for key measurements.

Clarify FBG fabrication and ML model parameters for reproducibility.

Expand discussion on model assumptions and potential temperature effects.

Version 1:

Reviewer comments:

Reviewer #2

(Remarks to the Author)
I would first like to thank and congratulate the authors for revising their manuscript exceptionally well. The manuscript, its aims and achievements are now very clear.

I would like to suggest a few very minor changes before publication.

1. Table S2 presenting the theoretical and experimental minimum radii of curvature is an important finding, and therefore should be presented in the main manuscript, and not the supplementary material. I suggest to place the Table in the main manuscript and refer the reader to the supplementary material for derivation and explanation.

2. Please state that the experimental demonstrations of mechanical vibration modes, speech recognition and hand gestures are presented to verify that the DFP process does not damage or degrade the FBGs, and that the adhesion to the substrate is good, enabling correct strain and motion determination.
All these FBG applications have been reported previously, and appropriate references should be given. The manuscript contains sufficient references to FBG mechanical vibration mode determination, but references to earlier reports of detecting speech recognition and hand gestures using FBGs should be added.

Reviewer #3

(Remarks to the Author)
After reviewing the revised manuscript, I confirm that the authors have adequately addressed the concerns raised in my initial report. The revisions have clarified the scope of the work and improved the presentation of the proposed methodology.

In my opinion, the issues identified in the first review have been satisfactorily resolved.

Updated recommendation: Accept.

Reviewer #4

(Remarks to the Author)
This manuscript presents a programmable Direct-FBG-Patterning (DFP) assembly paradigm for the one-step, high-precision, conformal integration of pre-inscribed, multiplexed Fiber Bragg Grating (FBG) sensor arrays onto arbitrary planar and non-developable curved surfaces. The approach is mechanically inspired by Direct Ink Writing (DIW) in additive manufacturing: a custom-built five axis motion platform—comprising three translational axes (x, y, z) on the upper print needle and two rotational axes (V , W) on the lower substrate—continuously guides a single optical fiber containing multiple pre-inscribed FBGs along programmed trajectories, bonding it to an adhesive substrate layer.
The key contribution is the development of a procedure that automates FBG placement. FO sensors have long been assembled manually, and the automated procedure here minimizes positioning error associated with the measurements. The framework is experimentally validated on three adhesive substrates (optically clear adhesive (OCA), nanotape, and double-sided tape), with assembly speeds of 2–5 mm/s and a guiding needle of internal diameter 260 μm.
The paper then demonstrates spatial cross-fiber and multilayer assembly strategies enabling highdensity, single-fiber multiplexed FBG sensor networks. Three system-level application demonstrations are presented:
1. Torsional displacement reconstruction in an aerospace-inspired wing-like structure using three FBG strain rosettes and the Modal Superposition Method (MSM);
2. Non-acoustic phonation monitoring on the laryngeal region with a 3 × 2 FBG array, a 3D convolutional recurrent neural network (3D-CRNN) with a dual-attention mechanism;
3. Epidermal hand-gesture recognition at the volar wrist using a CNN-GRU architecture.

The authors have successfully addressed all previous comments comprehensively.
The revised manuscript is notably cleaner in scope and framing. The responses to Reviewers 1 and 2 are thorough and scientifically honest, directly acknowledging the cross-disciplinary terminological ambiguity that caused the original confusion. The novelty lies in assembly science and integration
engineering, not in grating physics or deep-learning architectures. This is now clearly articulated.

The following comments are minor points:
1. Single-subject biological data: The phonation and gesture recognition experiments are performed on a single subject (implied by the protocol). Inter-subject variability is a well-known
limitation in epidermal sensing; acknowledging this explicitly and flagging it as future work would improve scientific rigor.
2. Long-term adhesive stability: The DFP assembly relies entirely on pressure-sensitive adhesives, yet no data on adhesion durability over time (e.g., peel strength after days or weeks, or under repeated bending cycles) are presented. For the aerospace SHM demonstration in particular, durability is a critical practical concern that deserves at least a brief comment.
3. Temperature compensation in practice: While the authors correctly note that all experiments were conducted at a stable 26 ± 0.5 °C and that reference-grating-based compensation is compatible with the DFP layout, no experimental demonstration of temperature decoupling is provided. The Discussion acknowledgement is appropriate, but a back-of-envelope estimate of temperature-induced strain crosstalk in realistic operating conditions would be informative for readers targeting field deployment.
4. Throughput and scalability: The assembly speed (2–5 mm/s needle feed) is mentioned in the Methods but the total time required to pattern a complete sensing array is not reported.
For readers considering practical adoption, a comparison of DFP assembly time against manual bonding would provide useful context.
The revised manuscript represents a scientifically sound and significant contribution to the field of FBG sensor integration. The authors have responded thoroughly to the first round of reviews, resolving
the major conceptual confusion regarding terminology, adding substantive optical and mechanical characterization data, and substantially improving reproducibility documentation.
The three-radius design framework is original and practically useful. The system-level demonstrations are impressive in scope, spanning rigid aerospace structures and compliant human tissue within a
single unified assembly paradigm. The paper is well-written, clearly structured, and the figures are of high quality.
2

Reviewer #5

(Remarks to the Author)
I co-reviewed this manuscript with one of the reviewers who provided the listed reports. This is part of the Nature Communications initiative to facilitate training in peer review and to provide appropriate recognition for Early Career Researchers who co-review manuscripts.

Version 2:

Reviewer comments:

Reviewer #2

(Remarks to the Author)
The authors have made all revisions/changes suggested by the reviewers.
The manuscript is suitable for publication.

I believe that there is a small typographical error on Page 7 of the supplementary material.
The text appears as
The experimental data, representing bending loss per unit length versus the
bend diameter, were plotted with error bars as shown in Fig. S2(c).

I think it should be Fig S4(b)

(Remarks on code availability)
DOI Not Found
10.6084/m9.figshare.31770808

This DOI cannot be found in the DOI System. Possible reasons are:

The DOI is incorrect in your source. Search for the item by name, title, or other metadata using a search engine.
The DOI was copied incorrectly. Check to see that the string includes all the characters before and after the slash and no sentence punctuation marks.
The DOI has not been activated yet. Please try again later, and report the problem if the error continues.

Reviewer #4

(Remarks to the Author)
The authors have addressed all the comments from the reviewer and this manuscript now can be accepted for publication in its current form.

(Remarks on code availability)
the DOI did not work

Reviewer #5

(Remarks to the Author)
I co-reviewed this manuscript with one of the reviewers who provided the listed reports. This is part of the Nature Communications initiative to facilitate training in peer review and to provide appropriate recognition for Early Career Researchers who co-review manuscripts.

(Remarks on code availability)

Open Access *This Peer Review File is licensed under a Creative Commons Attribution 4.0 International License, which permits use, sharing, adaptation, distribution and reproduction in any medium or format, as long as you give appropriate credit to the original author(s) and the source, provide a link to the Creative Commons license, and indicate if changes were made.*
In cases where reviewers are anonymous, credit should be given to 'Anonymous Referee' and the source.
The images or other third party material in this Peer Review File are included in the article's Creative Commons license, unless indicated otherwise in a credit line to the material. If material is not included in the article's Creative Commons license and your intended use is not permitted by statutory regulation or exceeds the permitted use, you will need to obtain permission directly from the copyright holder.
To view a copy of this license, visit https://creativecommons.org/licenses/by/4.0/

Response to reviewers' comments

We sincerely thank the reviewers for their constructive and insightful comments, which have greatly helped to improve the quality and clarity of this manuscript. In response to the reviewers' suggestions, we have carefully revised the manuscript and addressed all comments point by point. Reviewer comments are reproduced in *italic* font, followed by our corresponding responses. All revisions have been incorporated into the revised manuscript and can be readily identified in the updated version.

Reviewer #1 (Remarks to the Author):

Summary

The manuscript claims the development of a "direct-FBG-writing (DFW)" method enabling programmable, high-precision, one-step fabrication of monofil FBG sensors on planar and curved surfaces, with applications in SHM and human-activity monitoring. While the work presents interesting demonstrations of patterning optical fibers on adhesive films and surfaces, the manuscript fundamentally misrepresents its technical contribution. In its current form, it lacks scientific clarity, contains major conceptual inconsistencies about FBG fabrication, and does not provide the essential optical or material characterization required for any paper claiming advances in FBG sensor technology.

Response: We sincerely thank the reviewer for the careful reading of the manuscript and for providing detailed comments. We acknowledge that the original manuscript did not sufficiently clarify the scope and nature of the proposed technique, which may have led to misunderstanding regarding its technical contribution and its relationship to conventional FBG fabrication. In response, we have substantially revised the manuscript to address these concerns, and the following points require further explanation:

First, we must clarify our terminology "Direct-FBG-Writing (DFW)". In previous manuscript, DFW does not refer to the optical inscription of Bragg gratings (e.g., UV laser writing or phase-mask-based grating formation). Furthermore, all FBGs utilized in this study are pre-inscribed commercial sensors (SMF-28e+^{TM}, Corning

Incorporated), and the proposed paradigm does not involve any grating inscription process. Instead, the term DFW is derived from the direct-ink-writing (DIW) technology in the additive manufacturing community, where material is continuously deposited along programmed paths to construct predefined structures, as shown in Fig. R1. Similarly, in our approach, the “ink” is replaced by single-fiber multiplexed FBG arrays, which is directly patterned, positioned, and conformally assembled onto different adhesive substrates through a programmable process. Therefore, the innovation of this work lies not in the fabrication or inscription of FBG material itself, but in its programmable direct-FBG-patterning (DFP) assembly paradigm inspired by the DIW manufacturing process.

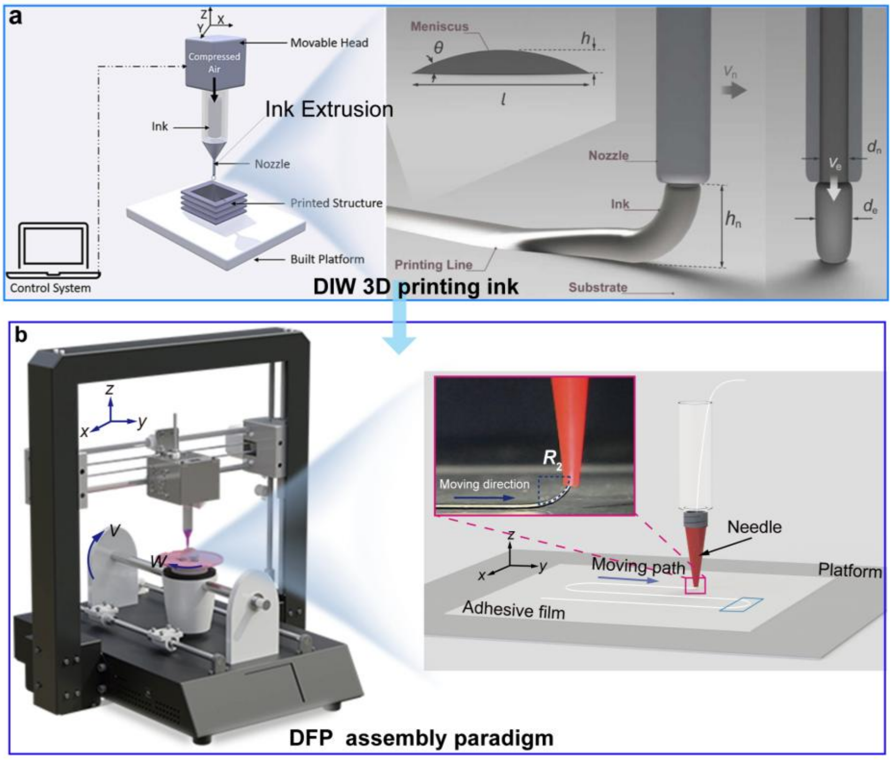

Fig. R1. (a) Schematic of conventional DIW involving path-defined material extrusion along programmed trajectories. (b) Implementation of the DFP integration technology. Inspired by the DIW concept, the proposed paradigm substitutes viscous ink with brittle multiplexed FBGs, enabling the automated assembly of quasi-distributed sensor arrays onto adhesive substrates.

Second, the reviewer’s concern regarding the lack of essential optical characterization is understandable if the manuscript is interpreted as proposing a new

FBG fabrication or inscription technique, which is not the core content of this work. The technical contribution of this study lies in integration and assembly science rather than grating physics. As shown Fig. R2, we establish a mechanics-optics coupled framework for the programmable assembly of single-fiber multiplexed FBG arrays, within which three critical minimum bending radii are derived and experimentally validated. These radii correspond to limits imposed by interfacial debonding, mechanical fracture, and bending induced functional optical attenuation, and together define the achievable geometric precision, integration density, and functional reliability during the direct writing assembly process. Consequently, the optical analyses presented in this manuscript serve as process critical validation for reliable assembly, rather than intrinsic characterization of FBG inscription properties.

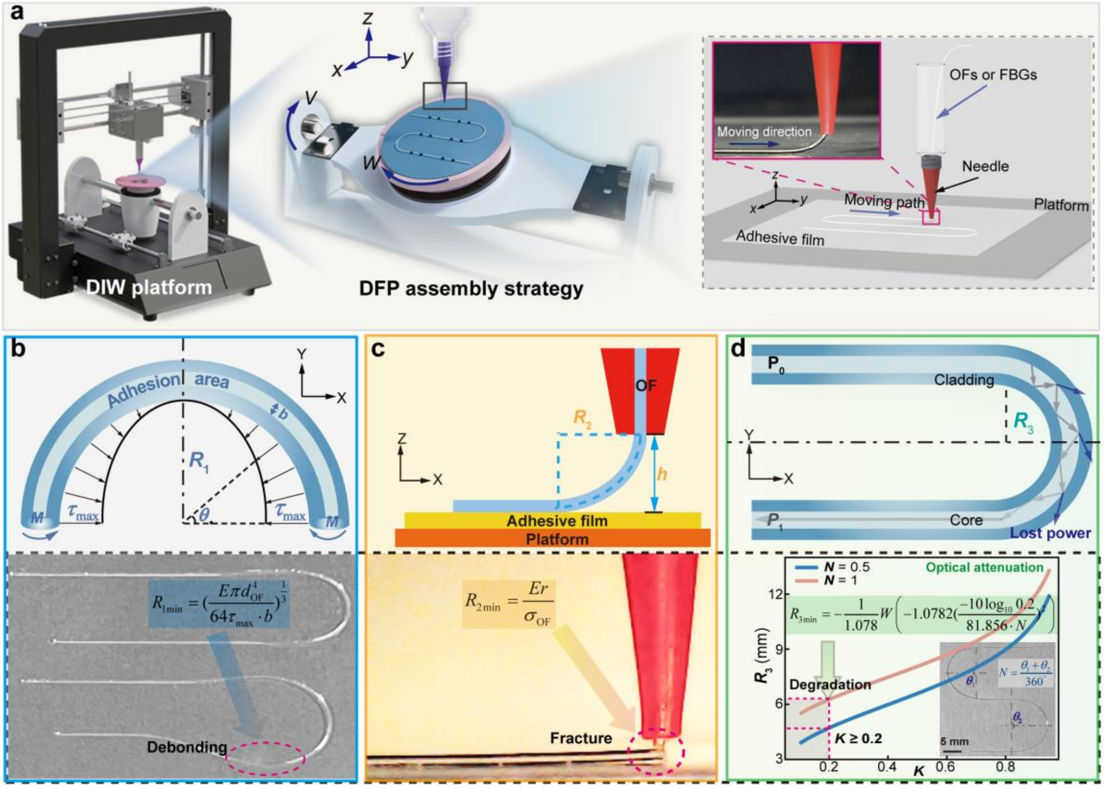

Fig. R2 Programmable DFP strategy and multi-radius constraint framework. (a) Schematic illustration of the automated DFP assembly system. A custom-built five-axis platform (*x*, *y*, *z* three translational degrees of freedom on the upper print needle and *xoy* (*W*), *xoz* (*V*) two rotational degrees of freedom on the lower substrate), inspired by DIW, enables programmable and high-precision path-defined assembly of multiplexed FBG arrays or bare OFs onto planar and curved substrates. **(b)** Interfacial stability

model (R_{1min}) to prevent OF debonding in the *x-y* plane. The continuum modeling accounts for the balance between the fiber's intrinsic bending moment and the interfacial shear stress (τ_{max}). The experimental image (bottom) shows interfacial debonding when the bending radius falls below the theoretical limit. **(c)** Mechanical integrity model (R_{2min}) to prevent brittle failure in the *x-z* plane during the assembly process. The bending radius R_2 is governed by the relative height (h) between the deposition needle and the substrate. Reducing h below the critical threshold leads to brittle fracture of the OF, as shown in the optical micrograph (bottom). **(d)** Functional reliability model (R_{3min}) based on macrobending-induced optical power attenuation. The schematic shows the power loss mechanism as light propagates through a curved waveguide. The corresponding graph identifies the functional patterning window where the output-to-input power ratio ($K = P_1/P_0$) remains ≥ 0.2, ensuring signal integrity for downstream multiplexed gratings.

Third, with respect to scientific novelty relative to existing FBG integration approaches, conventional deployment of FBG sensors typically relies on manual bonding, discrete sensor placement, or planar lamination, which inherently limits angular accuracy in multidirectional sensing, spatial resolution and unit density, and scalability to curved or non-developable surfaces. To the best of our knowledge, no prior work has demonstrated a programmable one step assembly approach capable of producing single-fiber multiplexed FBG arrays with extreme unit density and arbitrary three-dimensional conformality while simultaneously accounting for mechanical fracture, interfacial adhesion failure, and bending induced optical degradation. Moreover, the system-level demonstrations presented in this manuscript, including wing displacement reconstruction, human speech recognition, and gesture monitoring, are intended to validate the feasibility, robustness, and functional integrity of the proposed assembly strategy under real-world conditions. These examples are not meant to claim new sensing mechanisms, new FBG physics, or new interrogation principles, but rather to show that the DFW-enabled assembly process preserves the sensing performance of pre-inscribed FBGs while overcoming long-standing limitations associated with manual and planar integration.

Finally, to avoid further ambiguity, we have revised the manuscript to (i) replace "DFW" with "DFP" and explicitly clarify the definition and scope of DFP, (ii) distinguish it from optical grating inscription processes, and (iii) emphasize that the contribution lies in automated system integration and assembly paradigm, rather than FBG fabrication itself. Therefore, we hope these clarifications will eliminate previous conceptual inconsistencies and clearly position this paper as a contribution to advanced assembly methods and system-level deployment of FBG sensors, rather than grating writing technology itself.

Major Concerns

1. The manuscript is framed entirely around FBGs, but no actual FBG fabrication method is described

The title, abstract, and introduction repeatedly claim "direct writing of FBGs" using a novel "DFW" technique. However:

• Nowhere does the manuscript explain how the FBGs are inscribed in the fiber.

• There is no description of:

• The inscription method (UV writing? fs-laser? phase mask? point-by-point?).

• The refractive-index modulation mechanism.

• Photosensitivity treatment (hydrogen loading, Ge-doped fiber, etc.).

• Equipment, parameters, or setup.

• Actual FBGs only appear suddenly in Section 3 and 4 without any fabrication explanation.

Instead, the paper describes a method for mechanically placing and shaping pre-existing fibers/wires on adhesive films, not writing FBGs. For most of the paper, the authors use plain optical fibers as substitutes for FBGs (explicitly stated on page 7: "the OF was initially used as a substitute for FBG"). This contradicts the entire premise of "direct FBG writing".

Response: Thanks for this detailed and critical comment. We acknowledge that the current framing of the previous manuscript, particularly the repeated use of the term "direct writing of FBGs", may lead to confusion regarding the nature of the technical

contribution. We realize that the term “Direct Writing” carries fundamentally different connotations in two distinct scientific communities, which led to the conceptual ambiguity in our original submission. In the optical fiber community, “Direct writing” (e.g., UV or femtosecond laser writing) is a well-established term specifically referring to the internal modification of a fiber’s refractive index to create a grating. However, in the additive manufacturing community: “Direct writing”, exemplified by DIW, refers to a platform-controlled, path-defined process for depositing or patterning materials to form functional structures. Our work was initially framed from the perspective of additive manufacturing, where we treated the FBGs as a "functional filament" and used a robotic nozzle to "write" (pattern and integrate) it onto complex surfaces. We intended to describe a system-level assembly process rather than a component-level fabrication process. To eliminate this cross-disciplinary confusion and accurately represent our technical contribution, we have renamed our method from “Direct-FBG-Writing (DFW)” to “Direct-FBG-Patterning (DFP)” throughout the revised manuscript. This new terminology emphasizes the programmable spatial layout and integration of sensors, rather than the fabrication of the gratings themselves. Furthermore, we have explicitly clarified that the FBGs used in this study are pre-inscribed prior to the assembly process. The specific description is as follows:

In this work, we propose a programmable direct-FBG-patterning (DFP) assembly paradigm for the high-precision and one-step conformal integration of pre-inscribed FBGs onto arbitrary curved surfaces. Distinct from optical grating inscription, the proposed DFP approach focuses on the deterministic mechanical assembly and routing of OFs, enabling complex sensing layouts that are inaccessible to conventional manual bonding or planar lamination. Specifically, DFP employs a multi-degree-of-freedom motion platform, in which the fiber is continuously guided along predefined trajectories, analogous to path-defined material deposition in direct ink writing (DIW), but replacing extruded ink with solid-state fibers. **(Please see lines 58 to 66 of the revised manuscript).**

Therefore, regarding the preparation parameters and processes related to the FBG material itself, we would like to explicitly clarify that this work does not propose,

investigate, or claim any method for FBG inscription or fabrication. The manuscript does not involve UV writing, femtosecond laser inscription, phase-mask techniques, point-by-point writing, refractive-index modulation mechanisms, photosensitivity treatments, or any optical inscription equipment or parameters. All FBGs used in this study are commercially pre-inscribed using established industrial processes, and they are employed as-received without further optical processing. The relevant descriptions in the revised manuscript are as follows:

Materials and FBG specifications

Commercial single-mode OFs (SMF-28e+^{TM}, Corning Incorporated) were used throughout this study, either as bare fibers or with pre-inscribed fiber Bragg gratings (FBGs). The nominal fiber diameter, including the polymer coating, was approximately 242 ± 5 μm. Commercially pre-inscribed FBGs were employed for all functional sensing demonstrations. Each individual grating within the multiplexed arrays featured a length of 5 mm, with their respective central Bragg wavelengths distributed across the 1525-1575 nm range. This work focuses on the programmable assembly and integration of pre-inscribed FBG sensors rather than on optical grating fabrication. Therefore, no additional grating inscription or post-processing steps, such as UV inscription, femtosecond laser inscription, or phase mask-based etching, were performed. All FBGs were used directly without further optical processing. Simultaneously, for preliminary geometric patterning and assembly process development, bare OFs without gratings were employed as mechanically representative and cost-effective substitutes. This strategy facilitated a systematic exploration of routing geometries, bending limits, and multilayer assembly protocols while avoiding the risk of damaging brittle grating regions during the initial path optimization phase. These surrogate OFs ensured that the established minimum-radius assembly framework was rigorously validated before being applied to the functional FBG sensors. **(Please see lines 365 to 383 of the revised manuscript).**

Regarding the reviewer's observation that plain OFs were initially used as substitutes for FBGs, this is correct and intentional. The early-stage modeling and process validation focus on mechanical fracture, interfacial adhesion, and bending constraints,

which are governed by the fiber's geometric and mechanical properties rather than the presence of a grating. Using OF enables systematic and repeatable characterization of these assembly-limiting factors without risking premature damage to brittle grating regions. Once the process model and design criteria are determined, pre-inscribed FBG arrays were introduced for all functional sensing demonstrations, as shown in Section 2.3. This staged approach does not contradict the assembly paradigm. Rather, it reflects standard engineering practice in developing a robust manufacturing process for fragile functional elements. The description in the revised manuscript is as follows:

Simultaneously, for preliminary geometric patterning and assembly process development, bare OFs without gratings were employed as mechanically representative and cost-effective substitutes. This strategy facilitated a systematic exploration of routing geometries, bending limits, and multilayer assembly protocols while avoiding the risk of damaging brittle grating regions during the initial path optimization phase. These surrogate OFs ensured that the established minimum-radius assembly framework was rigorously validated before being applied to the functional FBG sensors. **(Please see lines 377 to 383 of the revised manuscript).**

Ultimately, we hope you understand that we want to emphasize that the core scientific contribution lies in the assembly paradigm rather than the fabrication of FBG material itself. Conventional FBG deployment relies heavily on manual bonding, discrete placement, or planar lamination, which limits angular accuracy, spatial density, and applicability to curved or non-developable surfaces. To our knowledge, this work is the first to demonstrate a programmable, one-step assembly framework capable of integrating pre-inscribed monofil FBG arrays with high unit density and arbitrary three-dimensional conformality, while explicitly accounting for mechanical fracture, interfacial debonding, and bending-induced optical degradation within a unified design framework.

2. What is the "optical fiber" the authors are printing? Is it a waveguide fabricated on the substrate? A real fiber? A polymer structure?

The manuscript repeatedly refers to the patterned material as "OF (optical fiber)", but

this raises multiple fundamental questions:

• Are the authors printing a material that functions as an optical waveguide?

• If yes, what material is this? What are its refractive-index properties? Mode field? Loss?

• How can FBGs be inscribed if the waveguide is created by deposition and not drawn as a fiber?

• If the authors are not printing the waveguide but simply placing a commercial fiber (e.g., SMF-28e+), then:

• Are the authors printing a material that functions as an optical waveguide?

• Why is this called "direct writing of FBGs"?

• Why is it called "monofil optical fiber"?

• Why is there no characterization of the mechanical and optical impact of adhesives used?

Response: We are sincerely grateful to the reviewer for the insightful and critical assessment of our work. We realize that the terminology used in the original manuscript was not sufficiently explained, leading to significant conceptual ambiguity regarding the nature of our technical contribution. We sincerely apologize for this. Regarding your questions, we address each point below.

First and most importantly, the material being patterned is a real, commercially drawn optical fiber, not a waveguide fabricated by material deposition on the substrate. In this work, the OF used is commercial Corning SMF-28e+, which possesses well-defined optical properties: a mode field diameter of 10.4±0.5 μm at 1550 nm, a typical effective refractive-index properties of 1.4682, and ultra-low attenuation (≤ 0.20 dB/km at 1550 nm). Our DFP process involves the platform-guided placement and patterning of these existing OFs onto adhesive substrates, rather than the fabrication of the waveguide itself. Therefore, no optical waveguide is created by deposition, printing, or curing of polymer materials, and no refractive-index engineering is performed on the substrate.

Accordingly, the patterned material is not a printed waveguide. It does not involve deposited polymer cores, claddings, or substrate-defined guiding layers. The

waveguiding properties (core/cladding structure, refractive-index profile, mode field diameter, and intrinsic propagation loss) are inherited directly from the original commercial OF. The relevant explanation is described in the revised manuscript as follows:

Materials and FBG specifications

Commercial single-mode OFs (SMF-28e+^{TM}, Corning Incorporated) were used throughout this study, either as bare fibers or with pre-inscribed fiber Bragg gratings (FBGs). The nominal fiber diameter, including the polymer coating, was approximately 242 ± 5 μm. Commercially pre-inscribed FBGs were employed for all functional sensing demonstrations. Each individual grating within the multiplexed arrays featured a length of 5 mm, with their respective central Bragg wavelengths distributed across the 1525-1575 nm range. This work focuses on the programmable assembly and integration of pre-inscribed FBG sensors rather than on optical grating fabrication. Therefore, no additional grating inscription or post-processing steps, such as UV inscription, femtosecond laser inscription, or phase mask-based etching, were performed. All FBGs were used directly without further optical processing. Simultaneously, for preliminary geometric patterning and assembly process development, bare OFs without gratings were employed as mechanically representative and cost-effective substitutes. This strategy facilitated a systematic exploration of routing geometries, bending limits, and multilayer assembly protocols while avoiding the risk of damaging brittle grating regions during the initial path optimization phase. These surrogate OFs ensured that the established minimum-radius assembly framework was rigorously validated before being applied to the functional FBG sensors. **(Please see lines 365 to 383 of the revised manuscript).**

Second, with respect to FBGs, the Bragg gratings are pre-inscribed within the fiber core prior to assembly, using standard industrial inscription techniques. As clarified in our response to question 1, this work does not fabricate, inscribe, or modify FBGs optically. Therefore, questions related to how gratings are inscribed in a deposited waveguide do not apply to the present study.

Regarding the explanation of the terminology:

As illustrated in Fig. R1, the term *"direct writing of FBGs"* was originally adopted by drawing inspiration from the DIW technology in the additive manufacturing community. This technology allows material to be continuously deposited along a predefined path to construct a pre-programmed structure. Analogously, in our approach, the "ink" is replaced by intact OFs or FBGs containing pre-inscribed multiple gratings, which are conformally assembled onto adhesive substrates using a custom-developed five-axis motion platform, thereby enabling their direct patterning along digitally defined trajectories. However, we later realized that the term "direct writing" has different meanings in different disciplines. In the optics and photonics community, it is often associated with direct grating inscription or laser writing, whereas in additive manufacturing it refers to path-defined material placement. Due to this cross-disciplinary ambiguity, combined with insufficient clarification in the original manuscript, the terminology may have been misinterpreted as implying direct FBG inscription, which was not our intention. To eliminate this potential confusion, we have replaced the term "DFW" with "DFP" throughout the revised manuscript, explicitly emphasizing that the proposed technique concerns programmable mechanical assembly and routing of pre-inscribed FBGs, rather than optical grating fabrication. This revision ensures terminological clarity and avoids misunderstanding regarding the nature of the technical contribution. The explanation of DFP in the revised manuscript is as follows:

In this work, we propose a programmable direct-FBG-patterning (DFP) assembly paradigm for the high-precision and one-step conformal integration of pre-inscribed FBGs onto arbitrary curved surfaces. Distinct from optical grating inscription, the proposed DFP approach focuses on the deterministic mechanical assembly and routing of OFs, enabling complex sensing layouts that are inaccessible to conventional manual bonding or planar lamination. Specifically, DFP employs a multi-degree-of-freedom motion platform, in which the fiber is continuously guided along predefined trajectories, analogous to path-defined material deposition in direct ink writing (DIW), but replacing extruded ink with solid-state fibers**. (Please see lines 58 to 66 of the revised manuscript)**.

The term "monofil optical fiber" was originally intended to emphasize that a single

continuous fiber filament is routed and assembled to realize high-density sensing networks, in contrast to conventional approaches relying on discrete, cut-and-bonded FBG segments. We acknowledge that the term "monofil" may introduce ambiguity from an optical fabrication perspective and does not precisely convey the intended system-level meaning. In the revised manuscript, we therefore replace this term with "single-fiber multiplexed FBG arrays" throughout, which more accurately reflects the fact that multiple gratings are integrated along a single continuous fiber to enable high-density, multifunctional sensing using one FBG strand. This clarification better aligns the terminology with established conventions in the FBG sensing community and eliminates potential misunderstandings.

Third, regarding the use of adhesive layers and their potential mechanical and optical impacts, this issue is explicitly addressed through the mechanics–optics coupled design framework developed in this work. The adhesive layer is not treated as a negligible or passive interface. On the contrary, its interfacial adhesion and mechanical compliance are central factors that are intentionally modeled, characterized, and exploited in the proposed assembly strategy.

Specifically, the interfacial shear strength (τ_{max}) of different adhesive films and the contact width (b) between the OF and the adhesive film are experimentally quantified and incorporated into the determination of the minimum allowable bending radius for reliable assembly. In parallel, the influence of macroscopic bending on optical transmission is explicitly considered through a functional attenuation model. Together, these analyses establish a well-defined design window that simultaneously avoids interfacial debonding, fiber fracture, and unacceptable optical loss. Therefore, the adhesive layer plays an active and indispensable role in the DFP process, rather than serving as an uncontrolled or secondary factor. The detailed process is as follows:

Quasi-static tensile tests were initially performed in triplicate following ASTM C1557-14 to estimate the elastic modulus. The linear elastic regime yielded a Young's modulus (E) of approximately 16880±733 MPa. However, post-peak stress reduction accompanied by coating-cladding separation indicated that tensile failure was governed by interfacial debonding rather than intrinsic fiber fracture (inset of Fig. R3(a)).

Therefore, tensile tests were unsuitable for evaluating the intrinsic fracture strength of the OF (σ_{OF}). To accurately determine the σ_{OF}, two-point bending tests were conducted. The OF was constrained within FDM-printed grooved fixtures (d_g = 0.3 mm) and subjected to displacement-controlled compression until fracture occurred (Fig. R3(b)). The fracture strength was calculated from the measured plate separation (d) at failure using the classical two-point bending relationship [4], as shown in following Eq. (S15):

$$\sigma_{OF} = \frac{1.198E \cdot d_f}{d - d_c + 2d_g}\left(1 + \frac{2.546 d_f}{d - d_c + 2d_g}\right) \quad \text{(S15)}$$

Based on five independent measurements, the values of d and the corresponding σ_{OF}, derived from a series of two-point bending experiments, are summarized in Fig. R3(c). The fracture strength was calculated as σ_{OF} = 1297±13 MPa, which was subsequently used for evaluating the R_{2min} model.

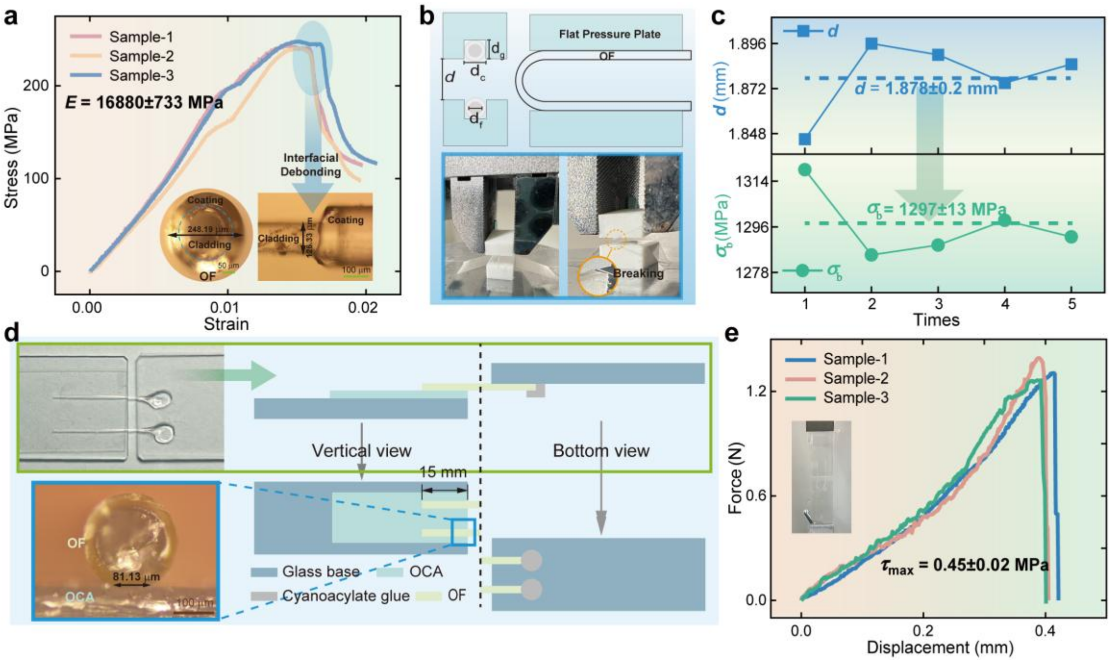

Fig. R3 (a) Quasi-static tensile stress–strain curves of the OF; (b) Schematic and photographic representation of the apparatus used to evaluate the intrinsic fracture strength of the OF; (c) Measured plate separation d and the corresponding calculated fracture strength σ_{OF}; (d) Schematic of the single-lap shear test setup and contact width b. (e) Force-displacement curves used to determine the maximum interfacial shear strength τ_{max}.

The τ_{max} between the OF and various adhesive substrates was characterized using a

standardized single-lap shear test [5]. As illustrated in Fig. R3(d), a double-sided optically clear adhesive (OCA, 3M8146-5) was bonded to a glass substrate. One end of the OF was bonded to the OCA film with a controlled contact length of L = 15 mm, while the opposing end was secured to a separate glass substrate using a cyanoacrylate adhesive (YNT-30). To enhance the interfacial force signal and ensure measurement reliability, a dual-OF design was employed. The effective contact width for a single OF on the OCA film (b_{OCA}) was measured via OM as 81.13±4.58 μm. Prepared samples were loaded in a universal testing machine at a constant displacement rate of 0.5 mm/min. The τ_{max} was calculated from the peak tensile force (F_{max}) using the Eq. (S16):

$$\tau_{max} = \frac{F_{max}}{2Lb} \tag{S16}$$

Based on three valid samples, the $\tau_{max\text{-}OCA}$ for the OF/OCA interface was determined to be 0.45±0.02 MPa (Fig. R3(e)). The same methodology was applied to evaluate the adhesion strength at the OF/nanotape and OF/double-sided tape interfaces (Fig. R4(a)). OM revealed mean contact widths of b_{nano} = 201.26±0.56 μm and b_{double} = 112.67±7.28 μm, respectively. Representative force-displacement curves for these interfaces are demonstrated in Figs. R4(b) and R4(c). Consequently, the maximum interfacial shear strengths were determined to be $\tau_{max\text{-}nano}$ = 1.41±0.08 MPa and $\tau_{max\text{-}double}$ = 0.86±0.03 MPa, respectively. **(Please see lines 89 to 134 of the revised Supplementary Material)**.

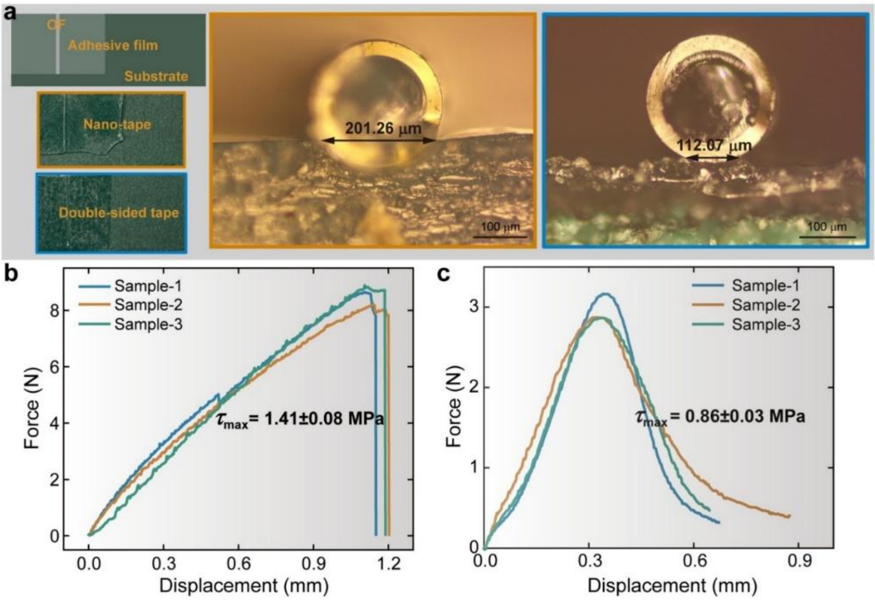

Fig. R4 (a) Contact widths at the interfaces between OF and nanotape or double-sided tape. The force-displacement curves of (b) OF/nanotape and (c) OF/double-sided tape tested by single-lap shear.

3. No characterization of the FBGs: optical spectra, Bragg wavelength, sensitivity, losses

A paper claiming to produce FBG sensors must provide:

- *Reflection spectra*
- *Peak wavelength and bandwidth*
- *Sidelobes*
- *Reflectivity*
- *Strain and temperature coefficients*
- *Linearity, cross-sensitivity, hysteresis*

None of these metrics appear anywhere in the manuscript.

Section 3–4 uses FBG arrays, but:

- *No spectral data are properly shown.*
- *No proof of grating functionality beyond assuming a strain–wavelength coefficient (0.0012 nm/με).*
- *No evidence that the gratings even exist in the fabricated fibers.*

This is unacceptable for a scientific paper about FBG fabrication.

Response: We appreciate the reviewer's careful assessment regarding the optical characterization of FBGs. We acknowledge that, in its original form, the manuscript did not sufficiently clarify the scope and intent of the optical characterization, which may have led to the impression that essential FBG fabrication and spectral validation were missing.

First, we emphasize that this work does not claim the fabrication manufacture the FBG itself or to inscribe the grating. The multiplexed FBG arrays used in this study are commercial-off-the-shelf components inscribed on standard Corning SMF-28e+™ fibers. According to the manufacturer's specifications, these industrial-grade FBGs typically exhibit a reflectivity > 90%, a full width at half maximum of ~0.2 nm, and a

sidelobe suppression ratio > 15 dB. Moreover, the focus of the manuscript is not on developing new grating structures or modifying their optical properties, but rather on establishing a programmable, automated assembly paradigm within the proposed theoretical framework of mechanics-optics coupling for integrating single multiplexed FBG arrays onto complex planar and non-developable surfaces. Accordingly, the novelty of this work lies in mechanical assembly, geometric routing, and high-density system-level integration, rather than in grating physics or photosensitive inscription processes.

Secondly, regarding the presentation of the FBG spectra, we realize that our previous descriptions may not have been sufficiently clear, which might have led to this information being overlooked. We wish to clarify that the reflection spectra shown in Fig. R5 provide direct experimental evidence for both the existence and the functional integrity of the FBGs used in this study. Specifically, Fig. R5(a)~ R5(d) displays multiple narrowband reflection peaks at well-defined Bragg wavelengths, which are quintessential optical signatures of FBGs. The emergence of these discrete peaks along a single continuous fiber clearly demonstrates that the fiber is inscribed with multiplexed FBG arrays, rather than being a plain optical fiber or a printed waveguide

Moreover, these spectra are measured after conformal assembly under different bending radii (R_3 = 4, 5, 6 mm). The preservation of sharp reflection peaks, stable peak positions, and comparable peak amplitudes demonstrates that: the gratings remain optically functional after DFP assembly; no grating erasure or catastrophic optical degradation occurs, and the proposed bending-radius constraint effectively preserves grating integrity.

The attenuation of some peaks in Fig. R5 is due to losses caused by fiber bending, rather than loss of grating function, which directly supports the mechano-optical coupling model discussed in this paper. This phenomenon is well explained in traditional FBG theory and does not require rewriting or modifying the grating. The revised manuscript describes this part as follows:

To characterize the signal integrity and spectral evolution of the assembled sensors, the reflected spectra of multiplexed FBG arrays were systematically monitored across

diverse routing geometries (Fig. R5). Two distinct FBG patterns were evaluated using an optical sensing interrogator: 2 × 2 arrays featuring a single bend (R_3 = 4 mm or 5 mm) and 3 × 2 arrays incorporating dual bends of equal radius (R_3 = 5 mm or 6 mm). Experimental results revealed that for single-bend patterns with R_3 = 5 mm, the optical power attenuation across downstream gratings remained within acceptable limits for reliable data acquisition. In contrast, reducing the radius to 4 mm induced severe macrobending losses, with the relative power ratio dropping below 0.2, which significantly degrades the signal fidelity. A similar trend was observed in double-bend patterns. While R_3 = 5 mm resulted in substantial power degradation at terminal gratings, the 6 mm radius maintained sufficient signal strength. Based on these empirical findings, a bend radius of 5 mm was selected for single-bend layouts, while 6 mm was adopted for double-bend designs. This selection achieves an optimal balance between device compactness and sensing reliability. Furthermore, these results provide experimental validation for the $R_{3\min}$ theoretical model, confirming its utility in guiding the design of functional, high-density FBG sensing networks. **(Please see lines 346 to 367 of the revised Supplementary Material)**.

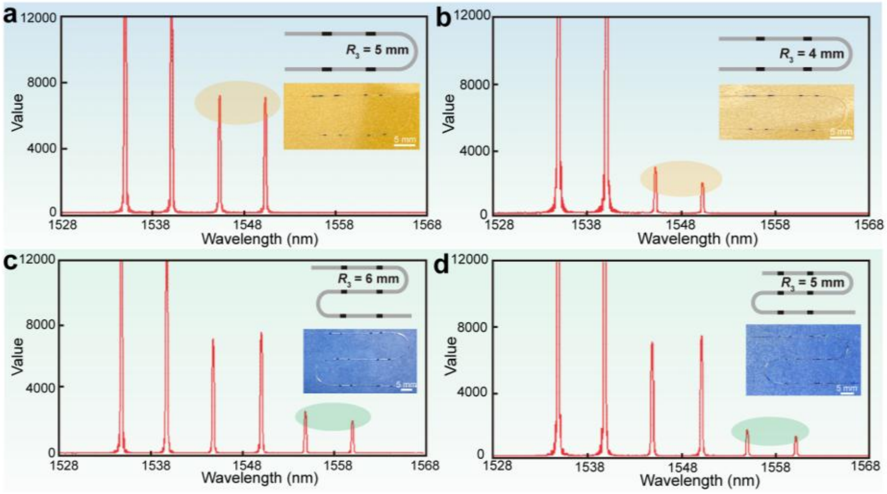

Fig. R5 Reflected spectra of multiplexed FBG arrays with different bending radii. Comparison for single-bend patterns (2 × 2 FBG arrays) with (a) R_3 = 6 mm and (b) R_3 = 6 mm. Comparison for double-bend patterns (3 × 2 FBG arrays) with (c) R_3 = 6 mm and (d) R_3 = 5 mm.

In summary, we emphasize that the purpose of this work is not to introduce a new FBG inscription method, but to demonstrate a programmable, system-level assembly

strategy that preserves the optical performance of commercially pre-inscribed FBGs while enabling unprecedented spatial density and three-dimensional conformality.

4. The manuscript describes only mechanical placement of fibers using adhesives, not FBG fabrication

Most of the paper focuses on:

• Bending-radius limits

• Adhesion mechanics

• Using nanotape or OCA

• Spatial positioning of pre-made fibers

• Routing fibers on 2D/3D surfaces

This is essentially a fiber-placement / assembly technology, not an FBG-writing technology.

The term "direct FBG writing" is therefore misleading. The novelty appears to be a robotic fiber-placement system, not an optical fabrication method.

Response: Thanks for your valuable comments. We wish to explicitly clarify that the claimed novelty of this manuscript does not reside in the domain of optical fabrication. We fully agree that the core technical contribution of this work does not lie in the optical inscription of FBGs themselves, but rather in the programmable, high-precision assembly and spatial routing of pre-inscribed FBG fibers into dense, conformal sensing networks on planar and non-planar substrates.

The core reason why the term "direct FBG writing" may be considered misleading lies in the fact that the meaning of direct writing differs substantially across disciplines. In the optics community, "direct writing" is typically associated with in situ grating inscription or laser-based writing processes. In contrast, within the additive manufacturing field, it generally refers to path-defined material deposition. Our proposal of the term DFW was fundamentally inspired by the latter method. However, owing to this interdisciplinary ambiguity, together with insufficient clarification in the original manuscript, the term could be misinterpreted as implying the direct inscription of FBGs, which was not our intention. Therefore, to eliminate this potential confusion,

we have revised the manuscript by replacing the term “DFW” with “DFP” throughout the text. We further explicitly clarify that the proposed technique focuses on the programmable mechanical assembly and routing of pre-inscribed FBG fibers, rather than on the fabrication or inscription of the gratings themselves. This revision ensures terminological clarity and prevents misunderstanding of the nature and scope of the technical contribution.

After clarifying the misunderstandings arising from the use of key terminology, we now clearly summarize the core technical innovations of this work, as follows:

Mechanics-optics coupled design framework

We propose a mechanics-optics coupled design framework that jointly considers bending-induced optical degradation, fiber fracture limits, and interfacial adhesion failure. While these constraints are known in practical FBG integration, existing approaches generally treat them independently and empirically, rather than through a unified design strategy.

Programmable five-axis conformal assembly

Based on this framework, a programmable five-axis assembly platform is developed, enabling arbitrary two-dimensional and three-dimensional conformal routing of pre-inscribed FBG fibers on planar and non-developable surfaces beyond the capability of planar lamination or manual bonding.

High-density patterning with single-fiber multiplexed FBG arrays

By combining the proposed design framework with spatial cross-fiber assembly, the method achieves ultra-high-density layouts using single-fiber multiplexed FBG arrays, a level of integration fundamentally inaccessible to manual, discrete cut-and-bond assembly.

System-level validation in previously impractical sensing patterns

System-level demonstrations showing that this assembly strategy enables sensing functions (e.g., strain rosettes, curved-surface displacement mapping, and epidermal phonation/gesture monitoring) that are difficult or impractical to achieve using conventional manual bonding or planar lamination approaches. These demonstrations highlight the scalability, robustness, and practical relevance of the proposed approach

at the system level, rather than claiming new FBG sensing physics.

From the perspective of these innovations, it becomes clear why substantial discussion is devoted to bending-radius limitations, adhesion mechanics, and the use of nanotape or OCA. These aspects are not peripheral process details, but constitute the theoretical modeling and experimental validation of the proposed mechanics-optics coupled design framework, as shown in Fig. R2. Systematic analysis of these factors is essential to establish the physical rationality, reliability, and practical applicability of the framework, and to ensure that the resulting FBG assemblies operate within mechanically safe and optically stable regimes. As shown in Fig. R6, the spatial positioning of pre-inscribed fibers and routing fibers on 2D/3D surfaces are therefore intended to realize integrated, programmable patterning within the proposed design framework. By leveraging the five-axis motion platform, this approach enables precise, continuous, and conformal fiber assembly along complex trajectories that are difficult or currently impossible to achieve through manual placement. This capability ensures that the theoretical constraints derived from the mechanics-optics coupled framework can be systematically enforced in practice, while simultaneously unlocking sensing layouts and unit densities beyond the reach of conventional manual fabrication methods.

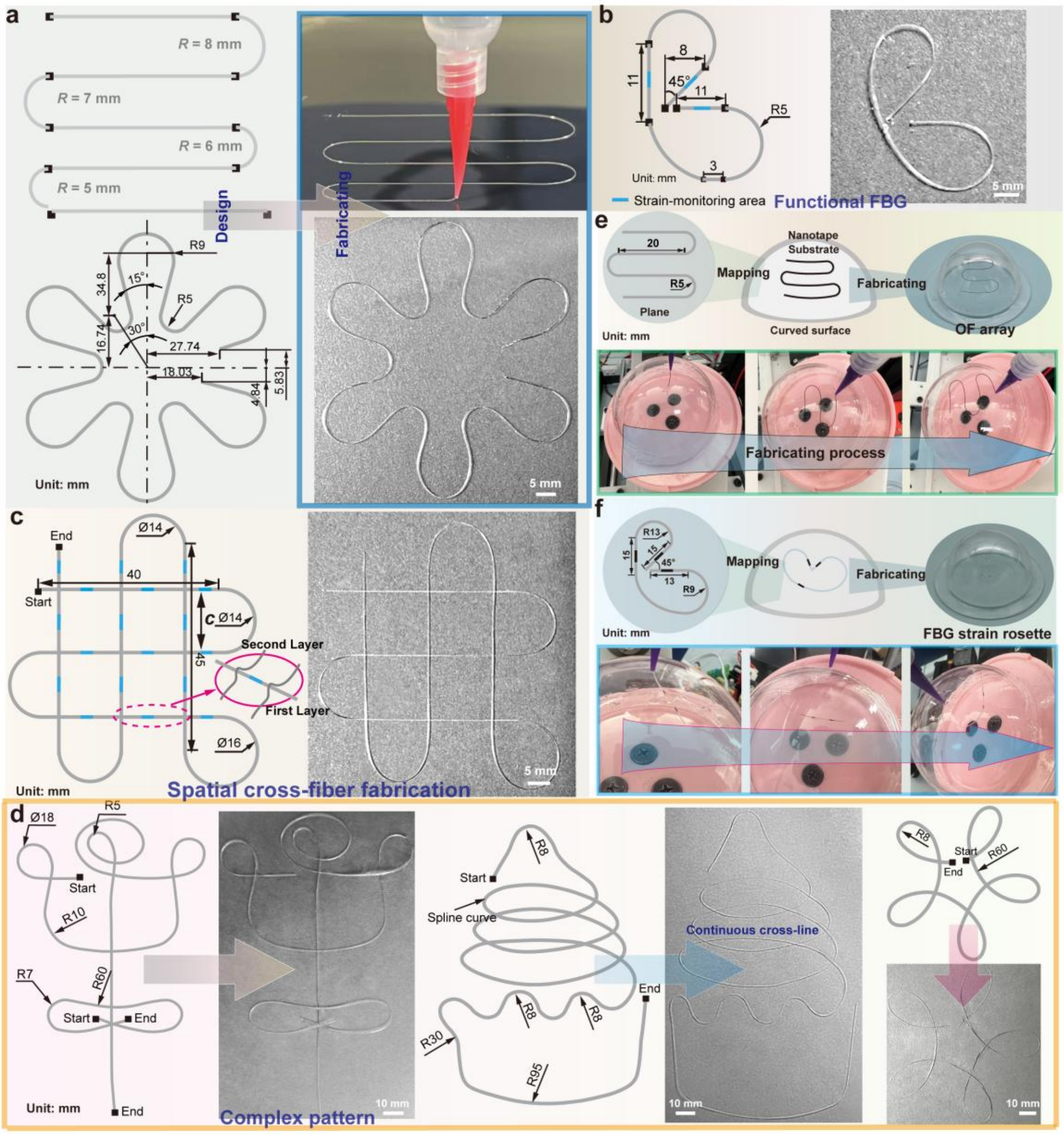

Fig. R6 Geometric patterning capability and functional FBG integration enabled by DFP. (a) Representative planar patterns with continuously varying curvature and sharp turns, demonstrating high-precision assembly layout over large areas. **(b)** Design and assembly of an orientation-defined triaxial strain-rosette, where the spatial layout of OF segments encodes directional sensing axes. **(c)** Schematic and optical micrograph of the multilayer fiber stacking process, achieved by precise vertical clearance control of the fiber-guiding needle. **(d)** Demonstration of complex, high-density architectures composed of intricate arcs, straight segments, and spline curves, highlighting the versatility of DFP for customized routing. **(e)** Continuous OF array assembled on a spherical shell substrate, validating the interfacial stability and conformity of the

patterning process on 3D geometries. **(f)** Functional FBG strain-rosette conformally integrated on a spherical surface. The five-axis DFP platform actively dynamically regulates the relative orientation between the nozzle and the substrate to mitigate the inherent brittleness of the grating-inscribed regions, ensuring reliable functional performance on complex structures.

5. No scientific novelty, primarily engineering assembly / applied demonstrations

The paper presents interesting demonstrations:

- *Patterned fiber routing*
- *Printing on curved surfaces*
- *Gesture and voice recognition using pre-existing FBG sensors*

However:

- *These do not constitute scientific advances in fiber optics.*
- *The "methods" are mechanical or procedural, not optical or material science.*
- *The use cases (SHM, human activity recognition) are straightforward applications of standard FBGs.*

The lack of scientific depth makes the manuscript unsuitable for Nature Communications.

Response: Thank you for your comment. First of all, thank you for finding our presentation interesting. However, the successful execution of these intricate routing patterns is precisely where our core innovation lies. We firmly believe that such results are fundamentally unattainable through conventional manual placement. The scientific value behind these "interesting" demonstrations stems from our ability to transition FBG integration from a manual "craft" to a deterministic assembly science. Thus, we respectfully disagree with the assertion that the manuscript lacks scientific novelty or that it represents only an engineering assembly exercise. This assessment arises primarily from interpreting the work as an attempt to advance fiber-optic physics, which is not the intent of this study. The scientific contribution of this work lies in the systematic integration science of FBG sensors, rather than in the discovery of new optical effects or grating inscription mechanisms. Specifically:

From "empirical manual assembly" to deterministic "assembly science": traditional FBG deployment is largely a manual "craft", characterized by discrete placement and trial-and-error bonding. Our work introduces a deterministic assembly paradigm. This is not merely a procedural change but a scientific leap in how brittle materials (silica fibers) are integrated into high-density, programmable networks. By establishing a mechanics-optics-coupled framework, we provide the first rigorous methodology to predict and prevent mechanical fracture and interfacial debonding while simultaneously optimizing optical signal integrity. These capabilities are fundamentally unattainable through manual methods.

Solving the challenge of conformal assembly on curved surfaces: A major scientific hurdle in flexible electronics is achieving conformality on non-developable surfaces (e.g., spheres, saddles). Traditional planar lamination or discrete placement inevitably leads to buckling or delamination. Our programmable DFP framework treats the fiber as a continuous, functional "ink" in a robotic deposition process, enabling arbitrary 3D conformality that respects the physical limits of the fiber. The use of industry-standard Corning SMF-28e+™ fibers ensures that our assembly-centric breakthroughs are built upon a reliable, high-performance physical foundation.

Deterministic multi-layer patterning for high-density array: within the proposed framework, a cross-line patterning strategy is introduced to overcome the layout limitations of conventional manual FBG integration. Traditional assembly typically relies on sparse, non-overlapping fiber placement to avoid mechanical damage and performance degradation. In contrast, the DFP approach exploits five-axis motion control to enable deterministic routing of a single OF or FBGs across intersecting paths and multiple layers, thereby achieving substantially higher spatial sensing density. The feasibility of this single-fiber multi-layer density is ensured by the mechanics-optics coupled design framework, which constrains bending, fracture, and adhesion conditions during assembly. As a result, the sensing density and spatial resolution achievable per unit area are significantly enhanced, allowing finer strain gradient mapping than is possible with conventional manual or planar integration methods.

In this sense, the manuscript contributes to integration science at the intersection of

mechanics, manufacturing, and sensing systems, which aligns with the scope of *Nature Communica*tions, where many prior studies emphasize enabling technologies that unlock new system-level capabilities through integration and design frameworks, rather than introducing new physical laws. To eliminate potential ambiguity and better reflect this contribution, the revised manuscript removes misleading terminology related to "direct-FBG-writing" redefines this work as a programmable assembly strategy and high-density integration design framework for single-fiber multiplexed FBGs, and clearly distinguishes optical grating fabrication from the proposed system-level sensor integration approach. The specific details can be found in our revised abstract and introduction, as follows:

Abstract: Fiber Bragg grating (FBG) sensors are widely employed in online monitoring of aerospace equipment and intelligent manufacturing due to their extremely high sensitivity, yet their deployment typically relies on skilled manual assembly, thereby significantly restricting achievable precision, integration density, and surface conformality under extreme conditions. Here, we propose a programmable direct-FBG-patterning (DFP) assembly paradigm for the one-step integration of brittle, multiplexed FBG sensor arrays onto arbitrary curved and non-developable surfaces. A mechanics-optics coupled framework is established to identify three critical minimum bending radii governed by interfacial debonding, fiber fracture, and bending-induced optical attenuation, which together define the achievable feature size and multidirectional sensing capability. Guided by this framework, spatial cross-fiber and curved-surface conformal assembly strategies are developed to enable high-density sensor networks realized by routing multiple FBGs along a single continuous optical fiber, comprehensively beyond the limits of conventional manual assembly. System-level demonstrations, including structural displacement reconstruction, human phonation, and gesture monitoring, validate the robustness and versatility of the proposed assembly paradigm. This study provides a fundamental strategy to unleash the full potential of quasi-distributed FBG sensors, thereby inspiring breakthrough applications that were previously constrained by the limitations of manual assembly. **(Please see lines 12 to 30 of the revised manuscript)**.

In this work, we propose a programmable direct-FBG-patterning (DFP) assembly paradigm for the high-precision and one-step conformal integration of pre-inscribed FBGs onto arbitrary curved surfaces. Distinct from optical grating inscription, the proposed DFP approach focuses on the deterministic mechanical assembly and routing of OFs, enabling complex sensing layouts that are inaccessible to conventional manual bonding or planar lamination. Specifically, DFP employs a multi-degree-of-freedom motion platform, in which the fiber is continuously guided along predefined trajectories, analogous to path-defined material deposition in direct ink writing (DIW), but replacing extruded ink with solid-state fibers. Central to this assembly paradigm is a mechanics-optics coupled design framework constituted by three critical minimum bending radii, which collectively define the feasible "assembly window" for brittle OFs. These radii are derived by jointly considering interfacial adhesion, fiber fracture, and bending-induced optical attenuation, thereby establishing quantitative constraints on routing curvature and minimum feature size. Building upon these constraints, DFP enables the conformal assembly of high-density, single-fiber multiplexed FBG arrays on complex curved surfaces with exceptional angular precision and spatial fidelity. This methodology effectively overcomes the inherent limitations in precision, scalability, and integration density that characterize traditional manual deployment. Leveraging this capability, we have successfully realized large-area and high-precision sensing arrays for dynamic response applications, including torsional displacement reconstruction in wing-like structures as well as human phonation and gesture monitoring. Consequently, this study not only enhances the monitoring accuracy of quasi-distributed sensor networks but also advances the assembly of FBG sensors from conventional structural health monitoring toward more complex and intelligent dynamic applications. **(Please see lines 58 to 82 of the revised manuscript)**.

6. Several contradictions and unclear statements

Examples:

• *DFW is described as a method to "directly write FBGs", but actual FBGs are written earlier via UV exposure and then manually assembled using the DFW method.*

• *The authors call OF patterns "functional FBGs" even when grating regions are not written.*

These internal contradictions severely damage clarity.

Response: Thanks for pointing out these issues. We acknowledge that certain descriptions in the original manuscript may have created apparent contradictions, primarily due to imprecise terminology rather than inconsistencies in the underlying methodology.

First, the term "direct FBG writing (DFW)" was originally introduced by analogy to direct-ink-writing (DIW) in additive manufacturing, referring to path-defined, continuous deposition of a pre-existing filamentary material. However, we recognize that in the optics community, "directly writing" is commonly associated with grating inscription. In the original version, this cross-disciplinary terminology was insufficiently clarified, which may have given the impression that FBGs were inscribed during the assembly process. To eliminate this ambiguity, we have replaced "DFW" with "direct FBG patterning (DFP)" throughout the revised manuscript and explicitly state that all FBGs are pre-inscribed using established optical inscription methods prior to assembly. The proposed technique therefore does not involve grating fabrication, but rather programmable routing and integration of FBGs. The specific description is as follows:

In this work, we propose a programmable direct-FBG-patterning (DFP) assembly paradigm for the high-precision and one-step conformal integration of pre-inscribed FBGs onto arbitrary curved surfaces. Distinct from optical grating inscription, the proposed DFP approach focuses on the deterministic mechanical assembly and routing of OFs, enabling complex sensing layouts that are inaccessible to conventional manual bonding or planar lamination. Specifically, DFP employs a multi-degree-of-freedom motion platform, in which the fiber is continuously guided along predefined trajectories, analogous to path-defined material deposition in direct ink writing (DIW), but replacing extruded ink with solid-state fibers. **(Please see lines 58 to 66 of the revised manuscript)**.

Second, with respect to the use of the term "functional FBGs" when OFs without

gratings were employed, we clarify that bare optical fibers were intentionally used in specific sections solely for mechanical characterization and process validation, such as bending-radius limits and fracture analysis. These experiments aim to verify the rationality of the mechanical model. In the revised manuscript, we have clearly distinguished between "OFs" used for mechanical studies and "FBGs" used for sensing demonstrations, and removed or revised any statements that could imply sensing functionality in the absence of grating regions.

2.2 Geometric patterning capability and functional FBGs integration enabled by DFP

Guided by the established bending constraints, the DFP process facilitates the deterministic patterning of OFs into geometries with spatial densities and routing complexities that surpass the limitations of conventional manual assembly. To systematically evaluate the geometric versatility of the process while decoupling it from grating-related fragility, standard single-mode fibers were first utilized as model filaments. Representative single-layer patterns featuring continuously varying curvature, sharp turns, and complex routing are shown in Fig. R5(a), demonstrating stable adhesion and accurate realization of digitally programmed routing paths over large substrate areas. These results underscore the fundamental geometric fidelity and operational robustness of DFP for assembling delicate fiber-based elements. Beyond purely geometric demonstrations, DFP further enables the direct construction of orientation-defined sensing structures through programmable fiber routing. As shown in Fig. R5(b), a triaxial strain-rosette layout is assembled by precisely assembling single OF at predefined angular orientations within a localized domain. While bare fibers were employed here, the spatial arrangement serves as a functional template that intrinsically encodes directional sensing capabilities. Consequently, the DFP process directly translates digital sensing motifs into physical architectures, requiring only the subsequent incorporation of FBGs to activate full multi-axial strain-sensing functionality. This approach eliminates the need for manual alignment or post-assembly adjustment, enabling the scalable and reproducible construction of complex, fiber-integrated sensor systems. To further increase integration density, the DFP process was

extended to enable spatial cross-fiber and multilayer assembly. By precisely controlling the relative height and orientation between the fiber-guiding needle and the substrate, fibers were routed across pre-bonded paths without inducing fracture or interfacial delamination (Fig. R5(c)). Precise control of vertical clearance is critical, as overlap at fiber crossings must be managed to maintain adhesion stability. Balancing these constraints allowed the successful assembly of complex multilayer patterns composed of arcs and spline curves, illustrating the versatility of DFP for customized, high-density fiber patterns (Fig. R5(d)).

Beyond planar substrates, DFP enables conformal patterning directly onto non-developable curved surfaces. Initial validation was performed using continuous OF assembled on a spherical shell (Fig. R5(e)), where the high compliant of the adhesive layer accommodated local curvature without inducing OF debonding. The five-axis motion platform accurately mapped the digitally defined routing paths onto the curved geometry, maintaining uniform in-plane spacing across the three-dimensional surface. These results demonstrate the capability of DFP to preserve geometric fidelity during surface-conformal assembly. The transition from bare OF to FBG assembly introduces additional manufacturing constraints due to the increased brittleness of grating-inscribed regions. To mitigate the elevated risk of fracture during complex routing, the five-axis DFP platform was actively exploited to dynamically regulate the relative orientation between the nozzle and the substrate. This adaptive orientation control minimizes out-of-plane curvature and local stress concentration within the fragile grating segments. Using this strategy, a functional FBG strain rosette was successfully conformally assembled on the spherical substrate (Fig. R5(f)). Beyond the representative substrates shown in the main text, the DFP strategy was further validated on a variety of planar and curved substrates with distinct stiffness and surface properties, demonstrating robust manufacturability and interfacial stability across materials (Section 3 of the Supplementary Material). The successful realization of these functional layouts confirms that DFP enables the direct translation of digitally designed sensing architectures onto complex three-dimensional structures without manual positioning or post-assembly correction. **(Please see lines 154 to 206 of the revised**

manuscript).

All relevant sections have been carefully revised to ensure consistent terminology, explicit definition at first occurrence, and a clear separation between optical grating fabrication, mechanical assembly, and system-level sensing demonstrations. These changes resolve the apparent contradictions and substantially improve the clarity and coherence of the manuscript.

Recommendation

I cannot recommend this manuscript for publication in Nature Communications in its current form. The core issue is that the manuscript presents itself as a breakthrough in FBG fabrication, but does not actually fabricate FBGs using the claimed method, nor provide any optical or material characterization expected for such work. The contribution appears to be an applied robotic fiber-placement technique, not a scientific advance in photonics. Given these major conceptual and scientific deficiencies, the manuscript requires major revision at minimum, and may not be suitable for this journal even after a significant revision.

Response: We thank the reviewer for the thorough evaluation and for clearly articulating the basis of their recommendation. We agree that, in its original form, the manuscript could be interpreted as positioning itself as a breakthrough in FBG fabrication, which would indeed require detailed optical inscription methodologies and comprehensive grating-level characterization. However, we respectfully emphasize that this interpretation arises from the discipline-dependent meaning of the term “direct writing”, rather than from any inconsistency in the technical intent or content of the work. In photonics, “direct writing” is commonly associated with in situ grating inscription or laser-induced refractive index modification, whereas in the additive manufacturing community it denotes path-defined, continuous material deposition. Our previous use of this term was strictly rooted in the latter manufacturing context.

As clarified throughout the revised manuscript, the proposed approach does not claim to fabricate or inscribe FBGs. All FBGs used in this study are pre-written using established inscription techniques. The core contribution lies instead in a programmable,

mechanics-aware assembly and patterning strategy for integrating single-fiber multiplexed FBG arrays into arbitrarily complex two-dimensional and three-dimensional surfaces with high spatial density and deterministic routing. To eliminate any remaining ambiguity, we have removed the term “direct FBG writing” and consistently reframed the method as DFP, explicitly distinguishing optical grating fabrication from system-level sensor integration.

From this corrected perspective, the manuscript does not aim to advance photonic inscription physics, but rather addresses a long-standing and underexplored bottleneck in FBG-based sensing systems: the lack of scalable, high-precision, and mechanically robust integration strategies for high-density, conformal FBG networks on non-developable surfaces. The introduced mechanics-optics coupled design framework explicitly accounts for bending-induced optical degradation, fiber fracture limits, and interfacial adhesion failure. These constraints critically govern real-world FBG deployment, but are rarely treated in a unified, quantitative manner. While the implementation leverages a robotic five-axis platform, the contribution is not limited to an applied placement technique. Instead, it establishes a generalizable integration science framework that links mechanical constraints, optical performance, and manufacturing path planning, thereby enabling sensing configurations (e.g., ultra-dense single-fiber multiplexed FBG arrays, spatial cross-line patterning, and conformal deployment on complex geometries) that are fundamentally inaccessible to conventional manual bonding or planar lamination approaches.

We respectfully believe that this type of enabling, system-level advance, in which new assembly and integration paradigms unlock sensing capabilities that were previously impractical or unattainable, is well aligned with the scope of *Nature Communications*. The journal has frequently published studies that bridge mechanics, manufacturing, and functional sensing systems without necessarily introducing new physical laws. The revised manuscript has been substantially restructured to reflect this positioning with improved scientific clarity and conceptual consistency. We therefore respectfully ask the reviewer to reconsider the manuscript in light of these clarifications and revisions.

Reviewer #2:

Review of manuscript submitted to Nature Communications: NCOMMS-25-79098 Programmable and high-precision direct-writing monofil fiber Bragg grating sensors: one-step conformal manufacturing with extreme unit density on curved surfaces by Yin Tao et al The authors describe a study inscribing Fiber Bragg Grating (FBG) arrays in predefined geometries attached to adhesive films which can be then affixed to an object of interest for sensing applications. Complex arrays with multiple FBGs can be fabricated. Applications demonstrated include strain measurement in aviation structures, and various movements (speech, breathing, hand gestures) in the human body. The manuscript is very long and is accompanied by an equally long supplementary chapter. The manuscript is written more in the context of a superficial description of capabilities, and not in the typical way a manuscript for a research journal should be presented (new and original research described fully). The superficiality is highlighted by the fact that the "direct-writing" is not described at all, neither is the "needle" apparatus (Fig 1) used to deposit the fiber in the desired geometry. In short, after reading the manuscript several times I was unable to ascertain what new and novel results the authors wish to convey. Direct writing FBGs on free standing fibers are well known and commercialized, bending radii limitations on fibers have been studied for decades, and FBGs have been used extensively for strain characterization and shape sensing. I suggest that the authors shorten the manuscript by removing all the non-original material. State clearly what is novel and original and describe fully. The demonstration and supplemental video of shape and motion sensing is not necessary, since many demonstration videos of FBG shape sensing are available via commercial manufacturers' websites and YouTube videos.

Response: We sincerely thank the reviewer for the careful reading of our manuscript and for the constructive comments. We would very much like to take this opportunity to clarify the scope, intent, and novelty of our work, which we acknowledge were not sufficiently clear in the original submission due to ambiguous terminology and overly broad presentation.

First, there's the interpretation of "Direct writing FBGs" and the perception of insufficient description. We agree with the reviewer that the original manuscript did not adequately clarify what is meant by “direct writing,” which led to understandable confusion. In photonics, “direct writing” is often associated with grating inscription, whereas in the additive manufacturing community it refers to path-defined material deposition (e.g., direct ink writing). Our use of this term was inspired by the latter, not by optical grating inscription. To eliminate this ambiguity, the revised manuscript has removed the term “direct writing (DFW)” entirely and replaced it with “direct FBG patterning (DFP)”, explicitly defined as a programmable mechanical assembly and routing strategy for pre-inscribed FBGs, rather than an optical grating fabrication process. We now clearly state that FBGs are pre-inscribed using established methods, and the contribution of this work lies in their deterministic, high-precision and one-step conformal integration onto planar and curved surfaces. The specific description in the revised manuscript is as follows:

In this work, we propose a programmable direct-FBG-patterning (DFP) assembly paradigm for the high-precision and one-step conformal integration of pre-inscribed FBGs onto arbitrary curved surfaces. Distinct from optical grating inscription, the proposed DFP approach focuses on the deterministic mechanical assembly and routing of OFs, enabling complex sensing layouts that are inaccessible to conventional manual bonding or planar lamination. Specifically, DFP employs a multi-degree-of-freedom motion platform, in which the fiber is continuously guided along predefined trajectories, analogous to path-defined material deposition in direct ink writing (DIW), but replacing extruded ink with solid-state fibers. Central to this assembly paradigm is a mechanics-optics coupled design framework constituted by three critical minimum bending radii, which collectively define the feasible “assembly window” for brittle OFs. These radii are derived by jointly considering interfacial adhesion, fiber fracture, and bending-induced optical attenuation, thereby establishing quantitative constraints on routing curvature and minimum feature size. Building upon these constraints, DFP enables the conformal assembly of high-density, single-fiber multiplexed FBG arrays on complex curved surfaces with exceptional angular precision and spatial fidelity. This

methodology effectively overcomes the inherent limitations in precision, scalability, and integration density that characterize traditional manual deployment. Leveraging this capability, we have successfully realized large-area and high-precision sensing arrays for dynamic response applications, including torsional displacement reconstruction in wing-like structures as well as human phonation and gesture monitoring. Consequently, this study not only enhances the monitoring accuracy of quasi-distributed sensor networks but also advances the assembly of FBG sensors from conventional structural health monitoring toward more complex and intelligent dynamic applications. **(Please see lines 58 to 82 of the revised manuscript)**.

In addition, the structure, working principle, and role of the guiding needle and five-axis motion platform are now fully described in the Methods and Supplementary Information, addressing the reviewer's concern regarding insufficient description of the apparatus. The specific description is as follows:

4.2 Five-axis kinematic platform and motion principle

To realize conformal deposition along spatial trajectories, a five-axis motion system was employed. The system consists of three translational degrees of freedom (x, y, z) and two rotational degrees of freedom (V, W), enabling synchronized control of nozzle position and orientation [7,8]. As shown in Fig. R7(a), a global Cartesian coordinate system is defined with respect to the motion platform. For an arbitrary target point on the substrate surface, its spatial position is expressed as $P = (x_1, y_1, z_1)$, and the corresponding surface normal vector is given by $\boldsymbol{L} = (i, j, k)$, which is obtained directly from the surface mesh during trajectory planning. Maintaining alignment between the nozzle axis and the local surface normal is essential to ensure stable contact conditions and minimize bending-induced stress during OF assembly. Through coordinated rotations about the V- and W-axes, the substrate is reoriented such that the local surface normal aligns with the global z-axis, as schematically shown in Fig. R7(b). After rotation, the transformed normal vector becomes $\boldsymbol{L}' = [0, 0, (i^2 + j^2 + k^2)^{1/2}]$. The required rotation angles θ_V and θ_W are determined from the components of the surface normal vector by appling this alignment condition. The corresponding rotation matrices are denoted as $\mathbf{R}_V(\theta_V)$and $\mathbf{R}_W(\theta_W)$, which describe rotations about the V- and W-axes,

respectively. The overall normal transformation can thus be expressed as Eq. (S18) ~ Eq. (S21):

$$\boldsymbol{L}' = \mathbf{R}'(\theta_{\mathrm{W}}) \cdot \mathbf{R}(\theta_{\mathrm{v}}) \cdot \boldsymbol{L} \tag{S18}$$

$$\mathbf{R}'(\theta_{\mathrm{W}}) = \mathbf{R}(\theta_{\mathrm{w}}) \cdot \mathbf{R}(\theta_{\mathrm{v}}) \tag{S19}$$

$$\theta_{\mathrm{V}} = \arccos\left(\frac{k}{\sqrt{i^2 + j^2 + k^2}}\right) \tag{S20}$$

$$\theta_{\mathrm{w}} = \arctan(i/j) \tag{S21}$$

Accurate coordinate transformation is essential for maintaining spatial synchronization between the nozzle and the curved substrate during the DFP process. When the substrate undergoes a rotation of θ_{V} around V-axis, the underlying base coordinate system shifts, causing the W rotation axis to deviate from its initial alignment with the global z-axis. Consequently, the rotation around the W-axis must be described by the modified transformation matrix $\mathbf{R}'(\theta_{\mathrm{W}})$, as defined in Eq. (S19). By applying these sequential transformations, the target point P within the local frame is mapped to its absolute coordinates P' = (x, y, z) in the global reference frame, as shown in Eq. (S22):

$$P' = \mathbf{R}'(\theta_{\mathrm{w}}) \cdot \mathbf{R}(\theta_{\mathrm{v}}) \cdot P \tag{S22}$$

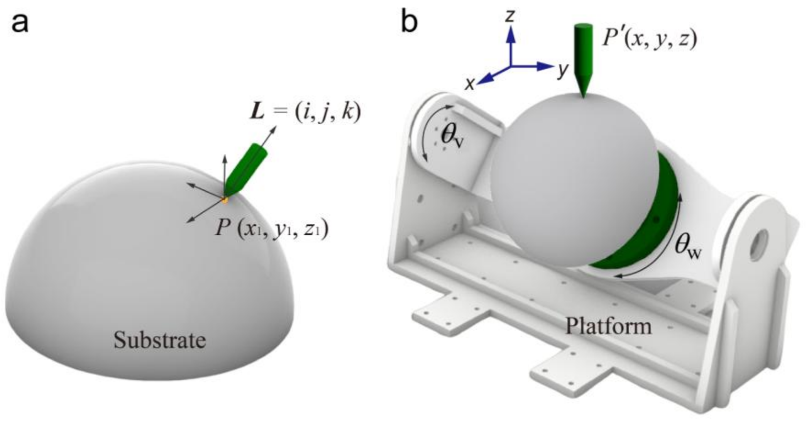

Fig. R7 Five-axis kinematic principle. (a) Definition of the target point $P = (x_1, y_1, z_1)$ on the substrate surface and its corresponding local surface normal vector $\boldsymbol{L} = (i, j, k)$ in the global coordinate system. (b) Schematic illustration of the coordinated rotations about the V- and W-axes that reorient the substrate such that the local surface normal

aligns with the global *z*-axis. The transformed target point P' = (x, y, z) and the corresponding rotation angles θ_V and θ_W are obtained after the kinematic transformation. **(Please see lines 288 to 327 of the revised Supplementary Material)**.

Second, regarding your assessment of the scientific novelty of our work in comparison to existing FBG technologies, we would like to provide further clarification. We fully agree that FBG inscription, bending-radius limits of fibers, and strain sensing using FBGs are well-established fields. Importantly, this work does not claim novelty in FBG physics, grating inscription, or basic sensing principles, and the revised manuscript now explicitly states this to avoid any misunderstanding. Instead, the novelty of this work lies in assembly science and integrating-enabled sensing, specifically:

A mechanics-optics coupled design framework is proposed that simultaneously accounts for fiber fracture during routing, interfacial adhesion failure, and bending-induced optical attenuation. These constraints that are typically treated independently but jointly limit practical FBG deployment.

A five-axis, programmable assembly strategy, developed within a mechanics-optics coupled design framework, that enables arbitrary 2D and 3D conformal routing of single-fiber multiplexed FBG arrays, including non-developable curved surfaces that are inaccessible to planar lamination or manual bonding.

A deterministic spatial cross-fiber and multi-layer patterning method that enables extreme spatial sensing density using a single continuous FBGs, far beyond what can be achieved by manual placement or discrete bonding.

These advances address long-standing practical bottlenecks in deploying high-density multiplexed FBG sensing networks and are not available in existing commercial or academic approaches.

Thirdly, regarding the reviewer’s concern that the manuscript was "superficial" and excessively long, we acknowledge that the original manuscript was overly long and included demonstrations that were not sufficiently contextualized in terms of their scientific purpose. In the revised version, we have substantially shortened and refocused the manuscript, removing non-essential demonstrations and repetitive descriptions. The

remaining application examples (e.g., structural deformation reconstruction and epidermal sensing) are retained not as claims of new sensing modalities, but as system-level validations showing patterns that are impractical or infeasible using conventional manual or planar integration methods. These demonstrations serve to verify the robustness, scalability, and practical relevance of the proposed assembly framework rather than to compete with existing commercial demonstrations. Furthermore, following your recommendations, we have streamlined the manuscript. By deleting non-essential materials or moving them to the Supplementary Material, we have made the central narrative and core innovations of this work more prominent.

Finally, we respectfully submit that this work represents an enabling, system-level advance, where a new manufacturing and integration paradigm unlocks sensing configurations that were previously impractical or unattainable. Nature Communications has a strong tradition of publishing such enabling technologies at the intersection of mechanics, manufacturing, and functional sensing systems, without requiring the introduction of new physical laws. To reflect this positioning with absolute clarity, the revised manuscript has been meticulously restructured to remove all misleading terminology related to FBG fabrication and to explicitly redefine our primary contribution as a programmable FBG assembly and integration strategy. This ensures a rigorous distinction between component-level grating inscription and the deterministic, system-level architectural integration of high-performance FBGs sensors. **(Please see the revised manuscript structure and Supplementary Material).**

Therefore, we sincerely hope that, in light of these clarifications and revisions, the reviewer will reconsider the manuscript's contribution and relevance.

Reviewer #3 (Remarks to the Author):

Overall Evaluation

This manuscript presents a comprehensive and technically solid study on the direct-FBG-writing (DFW) method for fabricating programmable, high-precision monofil fiber Bragg grating (FBG) sensors on curved surfaces. The work demonstrates high novelty by achieving one-step conformal manufacturing of FBG arrays with extreme

unit density and complex geometries, overcoming limitations of traditional manual FBG assembly methods. The experimental validation, theoretical modeling, and deep-learning-driven applications (speech and gesture recognition) are all of high interest to the optical sensing, additive manufacturing, and intelligent systems communities.

Response: We are sincerely grateful to the reviewer for their encouraging feedback and for recognizing the scientific novelty and technical solidity of our work. We are pleased that the reviewer appreciated the one-step conformal manufacturing and the extreme unit density of multiplexed FBGs sensor arrays achieved by our assembly strategy.

1. Noteworthy Results

The paper introduces a one-step DFW method capable of fabricating high-density, conformal FBG sensor arrays on both planar and curved substrates. The authors developed and experimentally validated three minimum bending radius models (R_{1min}, R_{2min}, R_{3min}) addressing fiber debonding, fracture, and optical degradation, respectively. This provides a robust theoretical foundation for DFW parameter optimization. Demonstrations include:

Torsional displacement reconstruction on wing-like structures for structural health monitoring (SHM). Phonation monitoring and gesture recognition using FBG arrays integrated with deep learning (MFCC–CRNN–LSTM architectures), achieving >95% accuracy. The integration of mechanical modeling, fabrication, and AI-driven sensing demonstrates strong interdisciplinary innovation.

Response: We sincerely thank the reviewer for the clear and accurate summary of our work and for recognizing the interdisciplinary nature of the contribution. We appreciate the reviewer's emphasis on the mechanics-optics coupled modeling framework, particularly the experimental validation of the three critical minimum bending radii ($R_{1\mathrm{min}}$, $R_{2\mathrm{min}}$, and $R_{3\mathrm{min}}$), which together establish a quantitative and practical design space for high-density, conformal FBG assembly.

We also thank the reviewer for acknowledging the system-level demonstrations, including torsional displacement reconstruction for structural health monitoring and intelligent phonation and gesture recognition enabled by deep learning. These examples

were intentionally selected to demonstrate how the proposed framework translates from theoretical constraints and programmable fabrication into reliable, application-relevant sensing systems.

In the revised manuscript, we have further clarified that the proposed one-step approach focuses on the programmable conformal assembly and routing of pre-inscribed, single-fiber multiplexed FBG arrays, rather than optical grating inscription itself, and have refined the terminology “direct-FBG-writing (DFW)” accordingly to avoid ambiguity. We believe this clarification, together with the integrated modeling-fabrication-AI workflow highlighted by the reviewer, better reflects the intended technical contribution and its interdisciplinary significance. Thank you again for your supportive comments and for recognizing the "strong interdisciplinary innovation" of our study. **(Please see the revised manuscript structure and Supplementary Material).**

2. Significance and Comparison to Literature

The work is of high significance for the advancement of FBG sensor integration and intelligent sensing technologies: Compared with traditional adhesive bonding methods [13–18], the DFW approach enables programmable, conformal, and large-area integration with enhanced precision and reproducibility. Previous reports (e.g., Guo et al., Composites Science and Technology, 2020; Lu et al., Soft Robotics, 2024) focused on flexible electronic or hybrid printed sensors, but none demonstrated direct optical fiber patterning with this level of mechanical and optical integrity. The proposed DFW platform bridges additive manufacturing and photonics, offering a route toward scalable smart-surface fabrication. The paper is well-positioned relative to state-of-the-art references in Nature Communications, Advanced Science, and IEEE Sensors Journal, showing clear original contribution.

Response: We are deeply grateful to the reviewer for their highly positive and professional evaluation of our work. We particularly appreciate the reviewer’s recognition of our study as a significant advancement in FBG sensor integration and their validation of our work’s positioning within the state-of-the-art research published

in *Nature Communications* and other high-impact journals. We thank the reviewer for pointing out that, compared with conventional adhesive bonding and manual integration strategies, the proposed approach enables programmable, conformal, and large-area integration of FBG sensors with substantially improved precision, reproducibility, and scalability. This capability constitutes one of the central innovations of the present work. By integrating path-defined automated assembly with a mechanics-optics coupled design framework, our method overcomes the intrinsic limitations of manual placement in terms of angular accuracy, unit density, and consistency, and enables, for the first time, deterministic routing and high-density integration of single-fiber multiplexed FBG arrays on complex two- and three-dimensional surfaces. This systematic and design-driven integration paradigm provides a practical and previously unattainable route for constructing large-area FBG sensing networks with complex topologies.

We also thank the reviewer for the thoughtful comparison with prior studies on flexible and hybrid printed sensors (e.g., *Guo et al.*, *Composites Science and Technology*, 2020; *Lu et al.*, *Soft Robotics*, 2024). While these works have made important contributions to printed and soft sensing systems, they do not address the deterministic routing and high-density integration of optical fibers under coupled mechanical and optical constraints. In contrast, our platform explicitly accounts for interfacial adhesion, fiber fracture limits, and bending-induced optical degradation, which are critical for preserving the functional integrity of FBG-based sensing networks. As highlighted by the reviewer, the proposed framework bridges concepts from additive manufacturing, mechanics, and photonic sensing, providing a practical pathway toward scalable fabrication of smart surfaces and conformal sensing architectures.

3. Validity of Data and Conclusions

The data analysis and interpretation are coherent and well supported by both theoretical and experimental results: The theoretical models of minimum bending radii are validated by empirical measurements with strong quantitative agreement (Table 2). Strain and displacement reconstruction experiments (Fig. 4) show consistent results

with finite element analysis. Machine-learning-based phonation and gesture recognition results are statistically well presented, with clear confusion matrices and accuracy curves (Figs. 5–6). However, two aspects could be improved:

Statistical robustness – The number of repetitions for sensor calibration and strain tests should be specified to assess variability.

Uncertainty quantification – Reporting standard deviations or error bars in optical attenuation and strain measurement data would enhance confidence in reproducibility.

Response: Thanks for this valuable suggestion. Clearly specifying the number of experimental repetitions is indeed crucial for demonstrating the statistical robustness and reliability of our results. Therefore, to enhance the statistical rigor of our study, we have explicitly specified the number of repetitions for sensor calibration and strain testing in the revised manuscript. Specifically, we have now listed the repetition counts for all critical calibration and testing steps in the revised manuscript section and Supplementary Material. The specific additions are as follows:

Parameter Measurement:

2.1 Characterization of mechanical and interfacial properties

The geometric and mechanical parameters required for model evaluation were experimentally characterized. As shown in Fig. S2(a), optical microscopy (OM) was used to determine the cross-sectional structure of the OF, revealing an acrylate coating and a silica cladding with diameters of d_c = 248.19±2.5 μm and d_f = 126.33±1.2 μm, respectively.

Quasi-static tensile tests were initially performed in triplicate following ASTM C1557-14 to estimate the elastic modulus. The linear elastic regime yielded a Young's modulus (E) of approximately 16880±733 MPa. However, post-peak stress reduction accompanied by coating-cladding separation indicated that tensile failure was governed by interfacial debonding rather than intrinsic fiber fracture (inset of Fig. S2(a)). Therefore, tensile tests were unsuitable for evaluating the intrinsic fracture strength of the OF (σ_{OF}). To accurately determine the σ_{OF}, two-point bending tests were conducted. The OF was constrained within FDM-printed grooved fixtures (d_g = 0.3 mm) and subjected to displacement-controlled compression until fracture occurred (Fig. S2(b)).

The fracture strength was calculated from the measured plate separation (d) at failure using the classical two-point bending relationship [4], as shown in following Eq. (S15):

$$\sigma_{\mathrm{OF}} = \frac{1.198E \cdot d_{\mathrm{f}}}{d - d_{\mathrm{c}} + 2d_{\mathrm{g}}}\left(1 + \frac{2.546d_{\mathrm{f}}}{d - d_{\mathrm{c}} + 2d_{\mathrm{g}}}\right) \tag{S15}$$

Based on five independent measurements, the values of d and the corresponding σ_{OF}, derived from a series of two-point bending experiments, are summarized in Fig. S2(c). The fracture strength was calculated as σ_{OF} = 1297±13 MPa, which was subsequently used for evaluating the $R_{2\mathrm{min}}$ model.

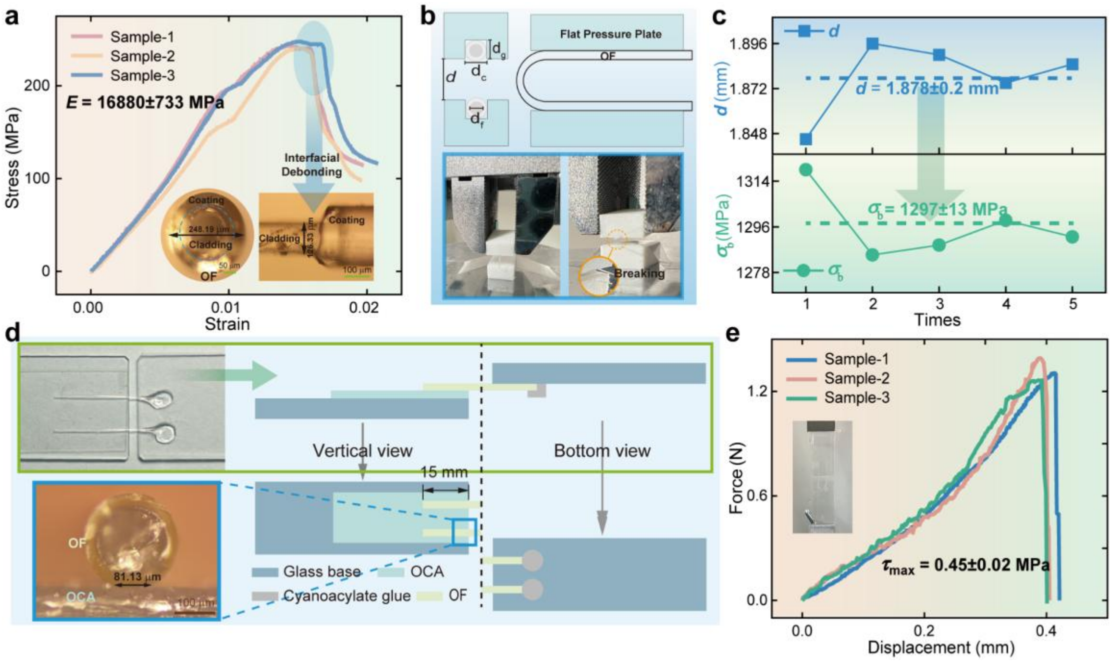

Fig. S2 (a) Quasi-static tensile stress–strain curves of the OF; (b) Schematic and photographic representation of the apparatus used to evaluate the intrinsic fracture strength of the OF; (c) Measured plate separation d and the corresponding calculated fracture strength σ_{OF}; (d) Schematic of the single-lap shear test setup and contact width b. (e) Force-displacement curves used to determine the maximum interfacial shear strength $\tau_{\max}$.

The $\tau_{\max}$ between the OF and various adhesive substrates was characterized using a standardized single-lap shear test [5]. As illustrated in Fig. S2(d), a double-sided optically clear adhesive (OCA, 3M8146-5) was bonded to a glass substrate. One end of the OF was bonded to the OCA film with a controlled contact length of L = 15 mm, while the opposing end was secured to a separate glass substrate using a cyanoacrylate

adhesive (YNT-30). To enhance the interfacial force signal and ensure measurement reliability, a dual-OF design was employed. The effective contact width for a single OF on the OCA film (b_{OCA}) was measured via OM as 81.13±4.58 μm. Prepared samples were loaded in a universal testing machine at a constant displacement rate of 0.5 mm/min. The τ_{max} was calculated from the peak tensile force (F_{max}) using the Eq. (S16):

$$\tau_{max} = \frac{F_{max}}{2Lb} \tag{S16}$$

Based on three valid samples, the $\tau_{max\text{-}OCA}$ for the OF/OCA interface was determined to be 0.45±0.02 MPa (Fig. S3(e)). The same methodology was applied to evaluate the adhesion strength at the OF/nanotape and OF/double-sided tape interfaces (Fig. S3(a)). OM revealed mean contact widths of b_{nano} = 201.26±0.56 μm and b_{double} = 112.67±7.28 μm, respectively. Representative force-displacement curves for these interfaces are demonstrated in Figs. S3(b) and S3(c). Consequently, the maximum interfacial shear strengths were determined to be $\tau_{max\text{-}nano}$ = 1.41±0.08 MPa and $\tau_{max\text{-}double}$ = 0.86±0.03 MPa, respectively.

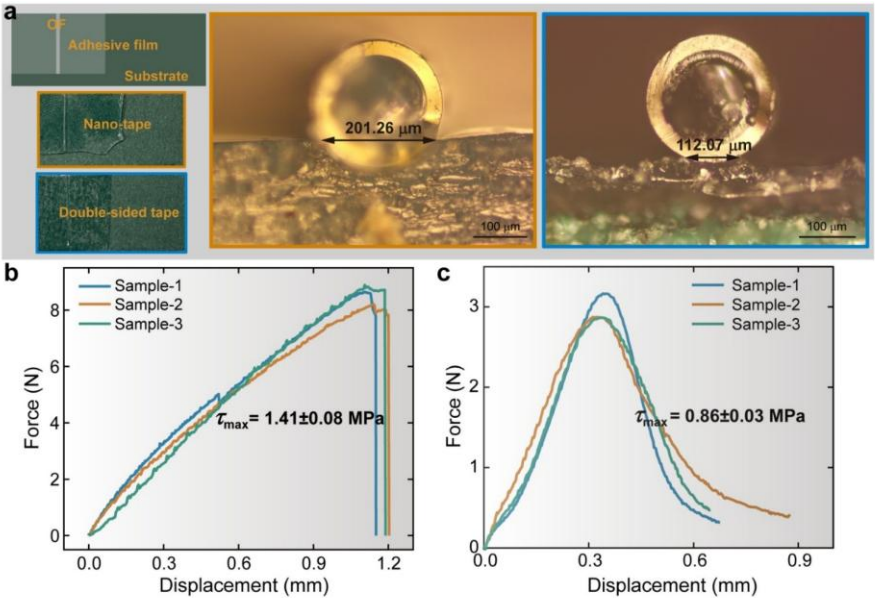

Fig. S3 (a) Contact widths at the interfaces between OF and nanotape or double-sided tape. The force-displacement curves of (b) OF/nanotape and (c) OF/double-sided tape tested by single-lap shear.

To determine the optical attenuation parameters involved in the macrobending loss model, independent bending experiments were first conducted on single-mode optical

fibers under controlled conditions. As shown in Fig. S4(a), the OF was wound once around cylindrical mandrels with different radii, and the transmitted optical power was measured using a constant-wavelength light source and an optical power meter. For each bending radius, measurements were repeated five times to ensure statistical reliability. The experimental data, representing bending loss per unit length versus the bend diameter, were plotted with error bars as shown in Fig. S2(c). By substituting these experimental results into the exponential relationship defined in Eq. (S11), the empirical parameters for the OF were determined as A = 13.0277 and B = -0.5391. Consequently, the calibrated formula for the loss per unit length is established as Eq. (S17):

$$\beta = \frac{13.0277}{\sqrt{R_3}} \cdot e^{-0.5391 \cdot R_3} \tag{S17}$$

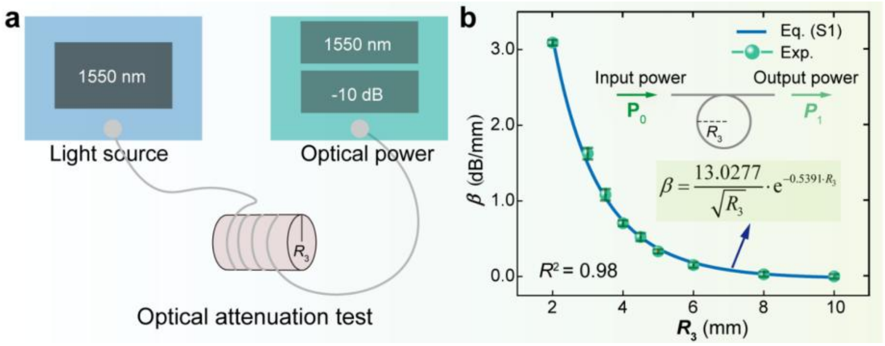

Fig. S4 (a) Schematic diagram of the bending-induced attenuation power test; (b) Relationship between attenuation per unit length and the bend radius of the OF. **(Please see the Section 2.1 of the revised Supplementary Material).**

Strain Tests (Functional Validation)

To validate the R_{3min} imposed by macrobending loss, optical power attenuation experiments were performed on the actual DFP-patterned structures. It is important to distinguish this validation from the initial calibration procedure. The initial calibration process involved winding the OF a full 360° around cylinders to extract parameters A and B, while this validation test measures the real-time loss of fabricated semicircular patterns (N = 0.5) with varying radii R_3. The experimentally measured loss values were compared with the theoretical predictions derived from the empirical attenuation model. As shown in Fig. S6(c), the measured optical loss closely follows the theoretical curve

over the tested bending radius range, with an average relative error of approximately 10%. The experimental results clearly demonstrate that as the bending radius R_3 decreases, the optical loss increases significantly, following the predicted exponential trend. The strong agreement between the measured data and the theoretical curve confirms that our model is reasonable and capable of accurately predicting optical attenuation across different radii. This confirms the model's reliability in determining the functional routing limits required to maintain signal integrity for multiplexed FBG arrays. **(Please see the lines 191 to 206 of the revised Supplementary Material).**

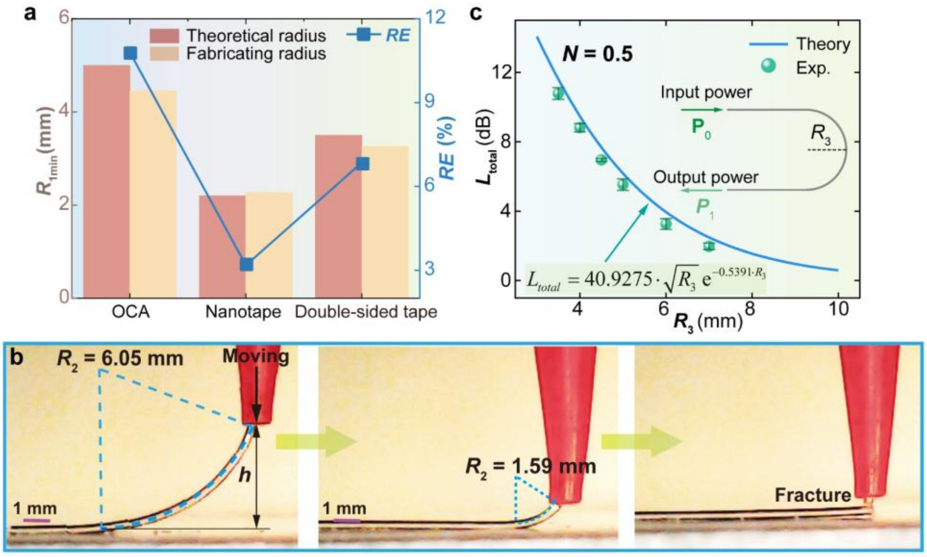

Fig. S6 (a) Comparison of theoretical (R_{1min-t}) and experimental (R_{1min-e}) values for three adhesive substrates. (b) Verification of the fracture limit R_{2min} through the controlled reduction of needle height h. (c) Validation of the functional limit R_{3min} by comparing measured optical loss with the theoretical curve.

To ensure statistical robustness and provide a comprehensive dataset for the deep learning model, each word was pronounced more than 200 times, with representative repetitions illustrated in the figures to demonstrate signal consistency. **(Please see the lines 410 to 212 of the revised Supplementary Material).**

Each sensing element captured distinct time-varying epidermal strain patterns associated with specific muscle activations, yielding repeatable and gesture-dependent signal signatures. To ensure statistical robustness, approximately 40 repetitions were recorded for each gesture type. The high strain sensitivity of the DFP-assembled FBG

sensors enables reliable detection of subtle musculoskeletal dynamics, providing suitable input data for gesture classification. **(Please see the lines 523 to 528 of the revised Supplementary Material).**

These additions do not alter the original conclusions, but rather reinforce the strong agreement between theoretical predictions, experimental observations, and numerical simulations.

4. Methodology and Reproducibility

The methodology is technically rigorous and well described. The use of continuum mechanics modeling, multi-axis DFW control, and deep learning for signal interpretation is appropriate and innovative. Still, for full reproducibility: The parameters for FBG laser inscription, adhesive curing conditions, and exact path-planning algorithm used in the 5-axis printing platform should be described in greater detail (perhaps in the Supplementary Material).

The training dataset size and augmentation details for the speech and gesture recognition models should also be provided for replication.

Response: Thanks for the positive assessment of the technical rigor and methodological soundness of our study. We agree that additional implementation details are important for ensuring full reproducibility.

First, regarding FBG laser inscription, we clarify that all FBGs used in this work are pre-inscribed using standard and well-established laser-writing techniques prior to assembly. As the focus of the manuscript is on programmable mechanical assembly and conformal integration rather than grating fabrication, the optical inscription process was not detailed. To improve clarity and reproducibility, the revised manuscript now clearly articulates this scope and provides an explanation in the material preparation section.

Materials and FBG specifications

Commercial single-mode OFs (SMF-28e+^{TM}, Corning Incorporated) were used throughout this study, either as bare fibers or with pre-inscribed fiber Bragg gratings (FBGs). The nominal fiber diameter, including the polymer coating, was approximately 242 ± 5 μm. Commercially pre-inscribed FBGs were employed for all functional

sensing demonstrations. Each individual grating within the multiplexed arrays featured a length of 5 mm, with their respective central Bragg wavelengths distributed in realistic 1525-1575 nm range. This work focuses on the programmable assembly and integration of pre-inscribed FBG sensors rather than on optical grating fabrication. Therefore, no additional grating inscription or post-processing steps, such as UV inscription, femtosecond laser inscription, or phase mask-based etching, were performed. All FBGs were used directly without further optical processing. Simultaneously, for preliminary geometric patterning and assembly process development, bare OFs without gratings were employed as mechanically representative and cost-effective substitutes. This strategy facilitated a systematic exploration of routing geometries, bending limits, and multilayer assembly protocols while avoiding the risk of damaging brittle grating regions during the initial path optimization phase. These surrogate OFs ensured that the established minimum-radius assembly framework was rigorously validated before being applied to the functional FBG sensors. **(Please see lines 365 to 382 of the revised manuscript)**.

Second, the description of the adhesive-assisted assembly process has been expanded in the Supplementary Material. This now includes the type of adhesive used and conditions, as well as their role within the proposed mechanics-optics coupled design framework.

Adhesive films and interfacial preparation

Three types of adhesive films were employed as substrates for fiber assembly: commercially available optically clear adhesive (OCA), nanotape, and double-sided adhesive tape. Prior to assembly, the adhesive films were laminated onto rigid or curved substrates and subjected to light uniform pressure to eliminate trapped air bubbles. No chemical surface modification or plasma treatment was applied. Importantly, these adhesive layers were not treated as passive bonding interfaces. Instead, they played an active mechanical role in the assembly process by providing sufficient interfacial adhesion to anchor the OF during extraction and assembly. The adhesive force ensures that the fiber can be continuously drawn from the feed and conformally deposited along the prescribed trajectory without slippage, buckling, or loss of positional accuracy,

thereby enabling deterministic path definition during five-axis assembly. The selection of adhesive films was based on the conformability and adhesion requirements of different application scenarios. OCA was primarily used for planar geometric patterning demonstrations due to its uniform thickness and optical clarity. Nanotape was employed for conformal assembly on curved and non-developable geometries owing to its enhanced compliance. For epidermal sensing applications on the laryngeal and wrist regions, double-sided adhesive tape was selected to ensure stable interfacial bonding under dynamic physiological deformation. All assembly processes were conducted under ambient laboratory conditions. **(Please see lines 384 to 403 of the revised manuscript)**.

Third, we have added a more detailed description of the path-planning and motion-control strategy implemented on the five-axis platform, including coordinate transformations, rotation sequencing, and kinematic constraints. Key algorithmic steps are now summarized in the Supplementary Material. The specific description is as follows:

4. Five-axis kinematic control and conformal trajectory planning for DFP assembly

4.1 Direct conformal trajectory planning on curved surfaces

Conformal fiber assembly on curved substrates requires motion trajectories that intrinsically follow surface geometry. Conventional approaches typically rely on a two-step strategy, where a planar trajectory is first designed and then projected onto a three-dimensional surface [6]. Such indirect projection inevitably introduces geometric distortion and trajectory twisting, which degrade placement accuracy and conformality. To address this limitation, a direct conformal trajectory planning strategy was adopted. As shown in Fig. S10, the procedure consists of four sequential steps. Step 1: Starting from the boundary of the curved surface, a set of concentric *V*-direction curves is generated by proportionally scaling the edge line toward the surface centroid. This operation preserves the global geometry of the surface while defining a structured set of reference curves. Step 2: Each *V*-direction curve is uniformly divided into the same number of discrete points. These points are organized into a matrix, denoted as *Point*,

where each row corresponds to a V-direction curve and each column represents points at the same parametric position along different curves. Step 3: By transposing the *Point* matrix to obtain $Point^T$, points sharing the same column index are interconnected, forming a family of W-direction curves. This matrix transposition operation establishes orthogonal curve sets without introducing geometric projection error. Step 4: The intersection of the W- and V-direction curves yields a structured surface mesh that conforms to the original geometry. Based on the desired sensing layout, selected mesh nodes are connected to define a polyline directly on the surface. The polyline is subsequently smoothed to obtain a continuous conformal trajectory. Finally, the conformal trajectory is offset along the local surface normal by a prescribed distance to define the motion path of the guiding nozzle during fiber assembly. This mesh-based planning approach enables precise, distortion-free trajectory generation on complex curved surfaces.

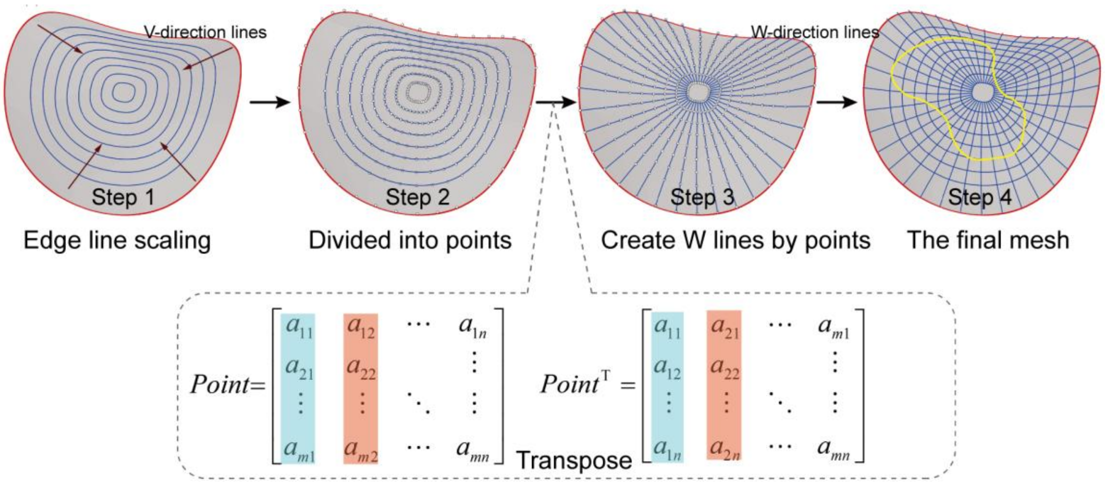

Fig. S10 Mesh-based conformal trajectory planning on curved surfaces.

4.2 Five-axis kinematic platform and motion principle

To realize conformal deposition along spatial trajectories, a five-axis motion system was employed. The system consists of three translational degrees of freedom (x, y, z) and two rotational degrees of freedom (V, W), enabling synchronized control of nozzle position and orientation [7,8]. As shown in Fig. S11(a), a global Cartesian coordinate system is defined with respect to the motion platform. For an arbitrary target point on the substrate surface, its spatial position is expressed as $P = (x_1, y_1, z_1)$, and the corresponding surface normal vector is given by $\boldsymbol{L} = (i, j, k)$, which is obtained directly

from the surface mesh during trajectory planning. Maintaining alignment between the nozzle axis and the local surface normal is essential to ensure stable contact conditions and minimize bending-induced stress during OF assembly. Through coordinated rotations about the *V*- and *W*-axes, the substrate is reoriented such that the local surface normal aligns with the global *z*-axis, as schematically shown in Fig. S11(b). After rotation, the transformed normal vector becomes $\boldsymbol{L}' = [0, 0, (i^2 + j^2 + k^2)^{1/2}]$. The required rotation angles θ_V and θ_W are determined from the components of the surface normal vector by appling this alignment condition. The corresponding rotation matrices are denoted as $\mathbf{R}_V(\theta_V)$and $\mathbf{R}_W(\theta_W)$, which describe rotations about the *V*- and *W*-axes, respectively. The overall normal transformation can thus be expressed as Eq. (S18) ~ Eq. (S21):

$$\boldsymbol{L}' = \mathbf{R}'(\theta_W) \bullet \mathbf{R}(\theta_V) \bullet \boldsymbol{L} \tag{S18}$$

$$\mathbf{R}'(\theta_W) = \mathbf{R}(\theta_W) \bullet \mathbf{R}(\theta_V) \tag{S19}$$

$$\theta_V = \arccos\left(\frac{k}{\sqrt{i^2 + j^2 + k^2}}\right) \tag{S20}$$

$$\theta_W = \arctan(i/j) \tag{S21}$$

Accurate coordinate transformation is essential for maintaining spatial synchronization between the nozzle and the curved substrate during the DFP process. When the substrate undergoes a rotation of θ_V around *V*-axis, the underlying base coordinate system shifts, causing the *W* rotation axis to deviate from its initial alignment with the global *z*-axis. Consequently, the rotation around the *W*-axis must be described by the modified transformation matrix $\mathbf{R}'(\theta_W)$, as defined in Eq. (S19). By applying these sequential transformations, the target point *P* within the local frame is mapped to its absolute coordinates *P*' = (x, y, z) in the global reference frame, as shown in Eq. (S22):

$$P' = \mathbf{R}'(\theta_W) \bullet \mathbf{R}(\theta_V) \bullet P \tag{S22}$$

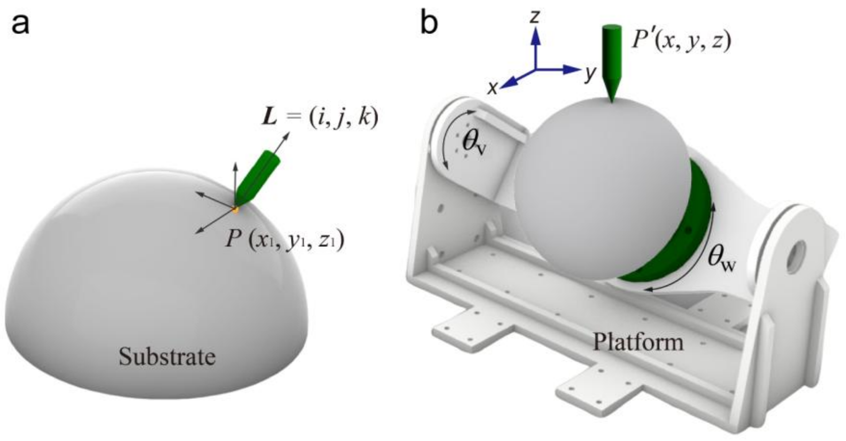

Fig. S11 Five-axis kinematic principle. (a) Definition of the target point $P = (x_1, y_1, z_1)$ on the substrate surface and its corresponding local surface normal vector $\boldsymbol{L} = (i, j, k)$ in the global coordinate system. (b) Schematic illustration of the coordinated rotations about the *V*- and *W*-axes that reorient the substrate such that the local surface normal aligns with the global *z*-axis. The transformed target point $P' = (x, y, z)$ and the corresponding rotation angles θ_V and θ_W are obtained after the kinematic transformation. **(Please see Section 4 of the revised Supplementary Material)**.

Finally, for the speech and gesture recognition tasks, the revised manuscript now specifies the training dataset size, data splitting strategy, and augmentation procedures (e.g., temporal windowing and noise perturbation). These additions ensure that both the sensing experiments and the deep-learning-based inference pipeline can be reliably reproduced. The specific details are as follows:

While these distinctive features provide a reliable foundation, the subtle differences in phonemic micro-vibrations and the high degree of feature coupling necessitate a sophisticated decoding framework to achieve high-accuracy, non-acoustic speech recognition in noisy or privacy-sensitive environments. Consequently, a non-acoustic speech recognition framework was developed based on epidermal strain signals acquired by a multi-channel FBG array. Fig. S19 illustrates in detail the signal processing flow, data augmentation strategies, and deep learning framework used for speech recognition.

A 3 × 2 FBG array conformally attached to the laryngeal region was used to capture multi-channel strain signals during phonation at a sampling rate of 1 kHz. Each sensing

channel recorded dynamic epidermal strain induced by laryngeal muscle activity during speech production. Raw strain signals were first subjected to baseline correction and denoising using discrete wavelet decomposition (Daubechies-4 wavelet, five-level decomposition) to suppress low-frequency drift and high-frequency noise while preserving speech-related vibration components. The processed signals were then amplitude-normalized to eliminate inter-trial variability and ensure consistency across samples. To fully characterize the temporal–spectral properties of the strain signals, both frequency-domain and time-domain features were extracted. Frequency-domain features were obtained using MFCC. The processing steps included pre-emphasis (coefficient 0.97), framing with a frame length of 40 ms and a frame shift of 20 ms. The first 13 cepstral coefficients were retained, followed by cepstral liftering (lifter coefficient = 22) to enhance high-frequency discrimination. In parallel, four statistical descriptors (mean, STD, skewness, and kurtosis) were computed for each channel in the time domain. The MFCC features and statistical descriptors were concatenated to form a composite feature representation. For each speech sample, the resulting feature tensor had a dimension of (6 channels, 17 features, 399 time frames), preserving both spatial and temporal information.

Given the limited size of the physiological speech dataset, a conditional Wasserstein generative adversarial network with gradient penalty (WGAN-GP) was employed for data augmentation. The generator received a 100-dimensional Gaussian noise vector conditioned on a 50-dimensional embedded class label and produced synthetic feature tensors with the same dimensionality as the real data ($6 \times 17 \times 399$). The network structure consisted of three successive upsampling blocks. The discriminator comprised four convolutional downsampling blocks with channel sizes of 32, 64, 128, and 256, followed by adaptive pooling and a fully connected layer to output the Wasserstein distance. Gradient penalty with a coefficient $\lambda = 10$ was applied to enforce the Lipschitz constraint, and the discriminator and generator were trained with a 5:1 update ratio. Using this strategy, 500 synthetic samples were generated for each speech category, expanding the training dataset by approximately one order of magnitude and effectively reducing overfitting. Speech classification was performed using a convolutional

recurrent neural network (CRNN) integrated with temporal and channel attention mechanisms. The input to the network was the standardized feature tensor with dimensions [B, 1, 6, 17, 399], where B denotes the batch size, and the second dimension represents a single input channel."

A 3D convolutional module was first employed to extract joint spatial–temporal features across sensor channels and time. The module consisted of two convolutional layers with 32 and 64 kernels of size 3 × 3 × 3, respectively. Each layer was followed by batch normalization, ReLU activation, dropout (0.3 and 0.2), and max pooling with a kernel size of 1 × 2 × 2. The output feature map was reduced to a dimension of [B, 64, 6, 4, 99]. The feature maps were then reshaped into a sequence of size [B × 6, 99, 256] and fed into a two-layer bidirectional long short-term memory (LSTM) with 256 hidden units and a dropout rate of 0.3 to model long-term temporal dependencies. To enhance discriminative capability, dual attention mechanisms were introduced. Temporal attention was implemented to assign adaptive weights to different time steps, allowing the model to focus on speech-critical segments. Channel attention was applied to dynamically reweight the contributions of the six FBG sensors, reflecting their relative importance during phonation. The attention-weighted features were aggregated into a 512-dimensional context vector. Finally, the aggregated features were passed through a fully connected classifier (512→ 256 → 128 → 6) with a Softmax output layer to predict the probability distribution over six target words (“And”, “No”, “Not”, “Ok”, “So”, and “Yes”).

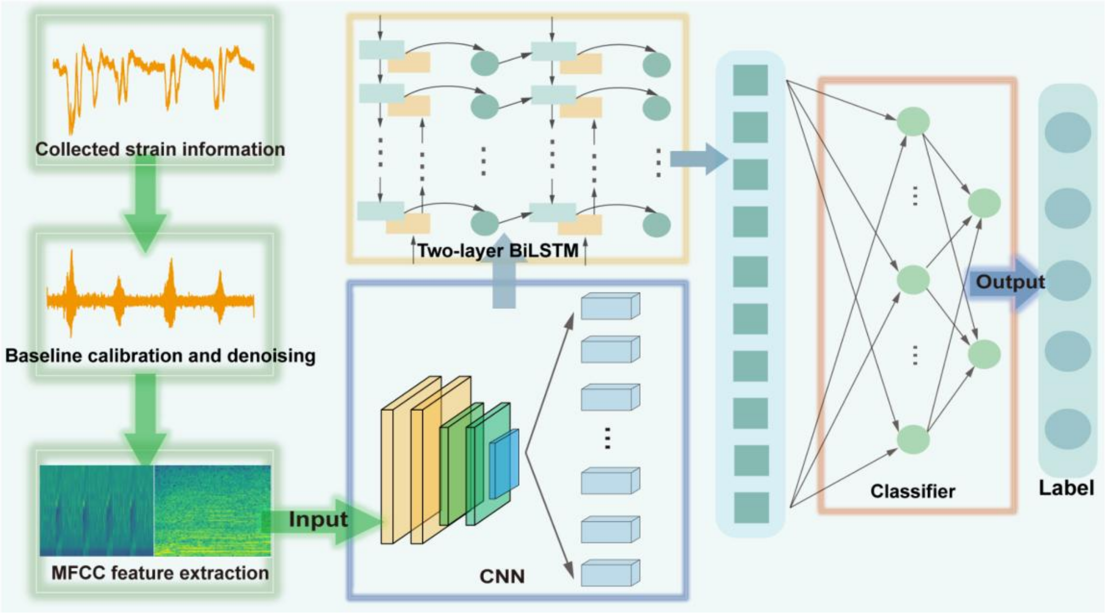

Fig. S19 Schematic of the hybrid deep learning framework for FBG-based signal recognition.

7.2 Intelligent hand gesture classification and kinematic decoding

As illustrated in Fig. S20(a), characteristic strain responses of the 3 × 2 FBG array were recorded during repetitive execution of three representative gestures (Gesture-4, Gesture-5, and Gesture-6). Each sensing element captured distinct time-varying epidermal strain patterns associated with specific muscle activations, yielding repeatable and gesture-dependent signal signatures. To ensure statistical robustness, approximately 40 repetitions were recorded for each gesture type. The high strain sensitivity of the DFP-assembled FBG sensors enables reliable detection of subtle musculoskeletal dynamics, providing suitable input data for gesture classification.

To decode the multi-channel temporal strain signals generated during complex hand movements, a hybrid deep learning framework combining convolutional neural networks (CNNs) and long short-term memory (LSTM) networks was developed [13,14], as shown in Fig. S20(b). The architecture is designed to jointly capture spatial distribution characteristics across sensing channels and temporal evolution patterns within each gesture. The input to the network consists of six-channel raw temporal strain signals with a dimensionality of [B, 6, T], where B denotes the batch size and T the number of time steps. The signals are first processed by a CNN-based feature extraction branch. A one-dimensional convolutional layer with a kernel size of 11 and

stride of 2 expands the feature space to 64 channels, enabling the capture of coarse temporal patterns over broad time windows.

Subsequently, the features are passed through three cascaded temporal feature extraction blocks based on depthwise separable convolutions, which decompose standard convolutions into depthwise and pointwise operations to reduce parameter complexity while preserving expressive power. These blocks progressively increase the channel dimensions to 128, 256, and 512, forming a multi-scale representation capable of modeling both short-term fluctuations and long-term gesture dynamics. Each block incorporates residual connections to stabilize training and integrated spatio-temporal attention mechanisms to adaptively emphasize informative time steps and feature channels. To accommodate variable-length gesture inputs, an adaptive max-pooling layer compresses the temporal dimension to a fixed length of 128, yielding a feature map of size [B, 512, 128]. This design ensures consistent computational complexity and facilitates real-time deployment. The feature map is then transposed to [B, 128, 512] and fed into a four-layer bidirectional LSTM (BiLSTM) network with a hidden size of 256. The bidirectional structure enables the model to capture contextual dependencies from both past and future temporal directions, which is critical for accurately modeling the complete progression of gesture execution. The BiLSTM output is further refined using dual attention mechanisms. Temporal attention assigns importance weights to different time steps to emphasize discriminative motion phases, while channel attention recalibrates the contributions of different feature dimensions. The resulting attention-weighted representation is subsequently passed through a three-layer fully connected classifier incorporating batch normalization, GELU/SiLU activation functions, and dropout regularization to map the extracted features to specific gesture categories. To ensure stable and efficient training, Kaiming normal initialization was applied to convolutional layers, and orthogonal initialization was used for LSTM weights, with the forget gate bias set to 1.0 to enhance long-term memory retention. By combining CNN-based spatial feature extraction with BiLSTM-based temporal modeling, the proposed framework provides an effective and computationally efficient solution for decoding multi-channel physiological micro-strain signals into distinct hand gesture

labels. **(Please see lines 456 to 571 of the revised Supplementary Material)**.

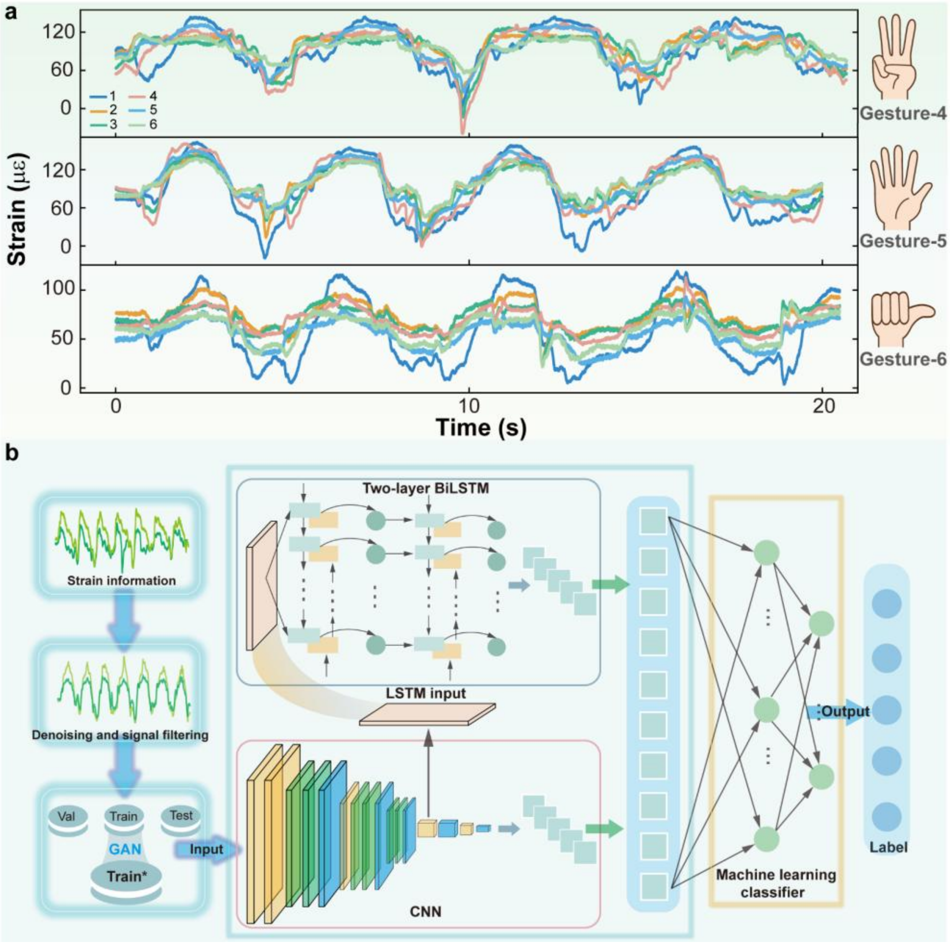

Fig. S20 (a) Multi-channel real-time strain signal responses corresponding to different hand gestures; (b) Schematic of the intelligent gesture recognition algorithm.

5. Potential Flaws and Suggestions

Model limitations: The models assume isotropic adhesive and uniform fiber stress distribution; non-uniform interfacial properties in nanotape could affect local debonding. Discussion on these simplifications would strengthen the analysis. Temperature compensation: Although tests are at 26 °C, real-world applications may face temperature variations. Incorporating a compensation model or noting the limitation would be valuable. Broader validation: Demonstrating DFW fabrication on non-polymeric substrates (e.g., metals or composites) could show wider applicability.

Response: Thanks for these constructive and insightful comments, which help further clarify the scope and limitations of the proposed framework.

First, model assumptions and interfacial non-uniformity are discussed. We acknowledge that the analytical models assume isotropic adhesive behavior and a quasi-uniform stress distribution along the fiber–adhesive interface. These assumptions were adopted to enable a tractable mechanics–optics coupled design framework that can provide clear, conservative design bounds for minimum bending radius and assembly feasibility. In practice, compliant adhesive media such as nanotape may exhibit local heterogeneity in thickness or viscoelastic response, which could induce spatially non-uniform debonding behavior. To address this point, we have added a discussion in the revised manuscript explicitly acknowledging this limitation and clarifying that the proposed models are intended to provide lower-bound design criteria rather than to capture all local interfacial phenomena. The specific details are described below:

A central outcome of this study is the development of a mechanics-optics-coupled framework that defines three critical minimum bending radii: $R_{1\mathrm{min}}$ (interfacial debonding), $R_{2\mathrm{min}}$ (fiber fracture), and $R_{3\mathrm{min}}$ (bending induced optical attenuation). These constraints collectively delineate the feasible design space for DFP-assembled FBG patterns, governing the minimum feature size and routing curvature. While the derived models treat adhesive layers as isotropic and homogeneous for theoretical clarity, we acknowledge that practical materials such as nanotape may exhibit viscoelastic behavior and local heterogeneity. Nevertheless, the strong agreement between our predictions and experimental results indicates that this framework provides a reliable design guideline for automated FBG assembly. **(Please see lines 334 to 343 of the revised manuscript)**.

Second, temperature effects and compensation have also been discussed and analyzed. We agree that temperature variation is an important consideration for real-world deployment of FBG sensors. In the present study, all experiments were conducted under controlled laboratory conditions (26 °C) to isolate and validate the mechanics–optics coupling associated with bending, adhesion, and routing. Temperature compensation coefficient was therefore not explicitly quantified in the current model. We have now clarified this point in the manuscript and noted that standard temperature compensation strategies for FBGs, such as reference gratings or differential

measurement schemes, can be readily integrated with the proposed assembly framework without modifying the mechanical design principles. Meanwhile, temperature-induced wavelength shifts in optical fibers and FBGs have been extensively studied, and well-established thermo-optic and thermo-mechanical models are available in the literature. Related models and discussions involving temperature coefficients have been summarized in the supplementary information to clarify their compatibility with the proposed framework. The specific description is shown in the following section:

Temperature sensitivity remains an inherent consideration for the real-world deployment of FBG sensing networks. Although all validation experiments in this study were performed under controlled laboratory conditions at 26 °C where temperature-induced wavelength drift was negligible, practical environments may require active or passive compensation strategies. Importantly, the DFP assembly paradigm is intrinsically compatible with established temperature compensation approaches, including reference gratings, differential configurations, and co-located sensing structures. The ability to precisely and programmatically position both sensing and reference FBGs within the same conformal layout provides a stable hardware foundation for reliable signal decoupling and downstream data processing. **(Please see lines 344 to 353 of the revised manuscript)**.

The optical responses of the DFP-assembled FBG arrays were assembled using a commercial FBG interrogator (ZX-SL-C16-1K). All Bragg wavelength signals were recorded at a constant sampling frequency of 1 kHz, enabling the capture of both quasi-static and dynamic strain responses. To convert the measured Bragg wavelength shifts into axial strain, a linear strain–wavelength relationship was adopted as Eq. (S23) [9-11]:

$$\varepsilon = \frac{[\Delta\lambda_{\varepsilon} - \Delta\lambda_{T} \cdot a]}{K_{\varepsilon}} \tag{S23}$$

where K_{ε} represents the strain sensitivity coefficient of the FBG, approximately 1.2 pm/με; $\Delta\lambda_{\varepsilon}$ and $\Delta\lambda_{T}$ denote the Bragg wavelength shifts induced by strain and temperature variations, respectively; and a is the temperature-dependent grating

coefficient. As all experiments were conducted under a constant ambient temperature of approximately 26°C, the temperature variation can be approximated as zero. Consequently, the influence of temperature on the Bragg wavelength shift is considered negligible in this study, and the sensing response is primarily attributed to mechanical strain. For applications involving large thermal fluctuations, the DFP assembly strategy is fully compatible with conventional temperature compensation schemes, such as reference gratings or differential measurement patterns. **(Please see lines 329 to 345 of the revised Supplementary Material).**

Finally, we thank the reviewer for the valuable suggestion to validate the fabrication on non-polymeric substrates, as such demonstrations would indeed broaden the applicability of the proposed approach. In response to this comment, we have conducted additional experiments to explicitly address this point. The corresponding results and descriptions have been incorporated into the revised manuscript and are detailed as follows:

As shown in Fig. S9(a) and S9(b), on planar composite substrates, DFP supports the assembly of complex layouts, including orthogonal grid and spiral patterns. The spiral pattern demonstrates that path-defined routing can accommodate structural features such as perforated laminates, allowing the sensing layout to be adaptively designed according to the actual geometry of the substrate. In addition, conformal assembly on curved substrates is achieved through coordinated multi-axis motion, enabling precise OF placement on stainless-steel and foam hemispheres (Fig. S9(c) and S9(d)). These demonstrations indicate that DFP is compatible with a wide range of substrate materials and geometries, and is not limited to planar fabrication, providing a general assembly strategy for integrating fiber-based sensors on complex engineering surfaces. **(Please see lines 243 to 257 of the revised Supplementary Material)**

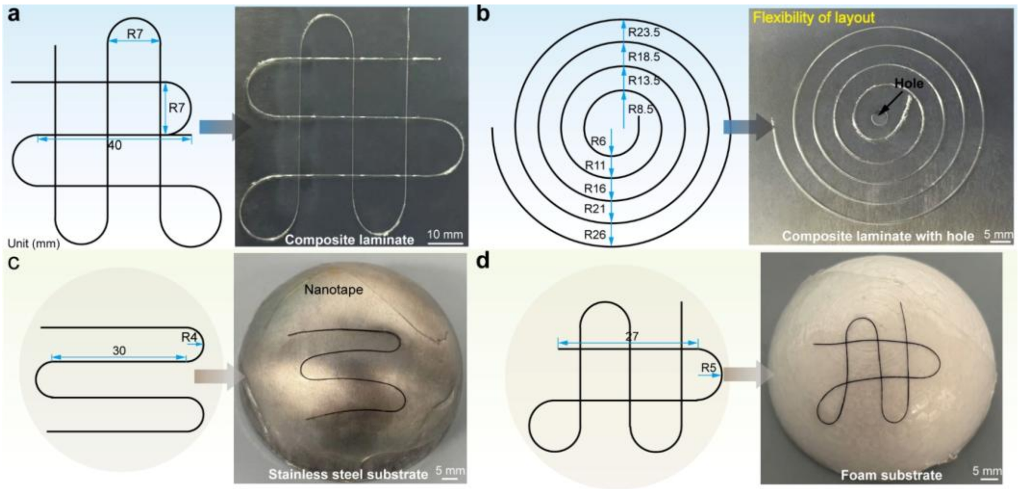

Fig. S9 OF patterning on composite laminates: (a) an orthogonal serpentine array and (b) a spiral layout navigating around a central hole, demonstrating layout flexibility. Conformal assembly on non-planar surfaces: (c) a hemispherical stainless-steel substrate and (d) a hemispherical foam substrate.

Overall, we believe these clarifications strengthen the rigor and transparency of the manuscript while maintaining its central contribution: mechanics-optics coupled, programmable assembly paradigm for high-density, conformal integration of single-fiber multiplexed FBG arrays

6. Recommendation

The manuscript presents a substantial and original contribution to optical sensor manufacturing and intelligent system integration. The methodology is sound, data interpretation is convincing, and the results are of broad interdisciplinary impact.

I recommend minor revisions before publication to address the following:

Provide statistical uncertainty or error estimates for key measurements.

Clarify FBG fabrication and ML model parameters for reproducibility.

Response: We sincerely thank the reviewer for the positive evaluation of the manuscript and for recognizing the originality, technical soundness, and interdisciplinary impact of this work. We also appreciate the constructive suggestions for further strengthening the manuscript.

In response, we have revised the manuscript to explicitly report statistical uncertainty

and error estimates for key measurements, including optical attenuation, strain response, and displacement reconstruction, by providing standard deviations and repeated-test statistics where applicable. These additions enhance the transparency and reproducibility of the experimental results. In addition, we have clarified the parameters associated with FBG fabrication and the deep-learning models to improve methodological reproducibility. Specifically, detailed descriptions of the FBG inscription process, adhesive preparation and curing conditions, multi-axis path-planning strategy, as well as the training dataset size, data augmentation procedure, and model hyperparameters for the machine-learning frameworks have been added to the Methods section, Results and Supplementary Material. We believe that these revisions fully address the reviewer's concerns and further strengthen the clarity and reproducibility of the manuscript.

Response to reviewers' comments

We sincerely thank the reviewers for their constructive and insightful comments, which have greatly helped to improve the quality and clarity of this manuscript. In response to the reviewers' suggestions, we have carefully revised the manuscript and addressed all comments point by point. For clarity, the reviewers' comments are presented in *black* italics, while our responses are in standard black font. All corresponding corrections in the revised manuscript and supplementary material are highlighted in blue font. We hope that these revisions satisfactorily address the concerns raised and that the manuscript is now suitable for publication in ***Nature Communications***. Thank you once again for your time and professional guidance.

Reviewer #2 (Remarks to the Author):

I would first like to thank and congratulate the authors for revising their manuscript exceptionally well. The manuscript, its aims and achievements are now very clear. I would like to suggest a few very minor changes before publication.

Response: We are deeply grateful for the reviewer's encouraging feedback and for the recognition of our revision efforts. It is heartening to know that the revised manuscript now clearly communicates the primary objectives and scientific achievements of this study. The reviewer's constructive suggestions in the previous round were indeed instrumental in improving the quality and clarity of our work. Regarding the "very minor changes" mentioned, we have addressed them point-by-point as follows and incorporated the necessary refinements into the revised manuscript.

1. *Table S2 presenting the theoretical and experimental minimum radii of curvature is an important finding, and therefore should be presented in the main manuscript, and not the supplementary material. I suggest to place the Table in the main manuscript and refer the reader to the supplementary material for derivation and explanation.*

   Response: Thank you for this insightful suggestion. The quantitative comparison between the theoretically predicted and experimentally measured minimum bending radii indeed provides important support for the proposed mechanics-optics coupled

framework. Following the reviewer's suggestion, Table S2 has been moved from the Supplementary Material to the main manuscript and is now presented as **Table 1**. The corresponding text in Section 2.1 has been revised to explicitly refer to this table, while the detailed analytical derivations and validation process remain provided in the Supplementary Material. The specific revisions are as follows:

Together, this coupled mechanics-optics framework defines the achievable feature size, routing density, and functional integrity of quasi-distributed DFP-assembled FBG arrays. To validate its predictive capability, experimental measurements were conducted on several adhesive substrates, including optically clear adhesive (OCA), nanotape, and double-sided tape. The experimentally determined critical minimum bending radii are summarized in Table 1. The close agreement between experimental measurements and theoretical predictions confirms the robustness of the proposed framework. Detailed analytical derivations and experimental procedures are provided in Sections 1 and 2 of the Supplementary Material. These thresholds establish practical design constraints for the automated DFP assembly process, ensuring structural stability, mechanical integrity, and signal fidelity. **(Please see the blue words on page 5 of the revised manuscript).**

Table 1 The minimum radius values on different adhesive films.

Adhesive film materials	$R_{1\text{min-e}}$ / $R_{1\text{min-t}}$ (mm)	$R_{2\text{min-e}}$ / $R_{2\text{min-t}}$ (mm)	$R_{3\text{min-t}}$ (mm)
OCA	5 / 4.48	1.59 / 1.64	4.7 (N = 0.5)<br>6.3 (N = 1)
Nanotape	2.2 / 2.25		
Double-sided tape	3.5 / 3.23		

Note: $R_{1\text{min-e}}$ and $R_{1\text{min-t}}$ represent the experimental and theoretical minimum radii for interfacial stability, respectively. $R_{2\text{min}}$ and $R_{3\text{min}}$ denote the limits for mechanical integrity and optical attenuation. N represents the proportion of the total bending angle of the optical fiber relative to the entire circumference. OCA, optically clear adhesive.

In summary, the critical minimum radii derived from the proposed mechanics–optics coupled framework have been validated across various adhesive substrates. A comprehensive comparison between the theoretical predictions and experimental

measurements is presented in Table 1 of the main manuscript. These validated thresholds provide essential design criteria and operational guidelines to ensure the structural and functional integrity of the FBG sensors during the automated DFP process. **(Please see the blue words on page 11 of the revised Supplementary Material).**

2. *Please state that the experimental demonstrations of mechanical vibration modes, speech recognition and hand gestures are presented to verify that the DFP process does not damage or degrade the FBGs, and that the adhesion to the substrate is good, enabling correct strain and motion determination. All these FBG applications have been reported previously, and appropriate references should be given. The manuscript contains sufficient references to FBG mechanical vibration mode determination, but references to earlier reports of detecting speech recognition and hand gestures using FBGs should be added.*

   Response: We sincerely thank the reviewer for this insightful comment, which helps to clarify the underlying motivation and scientific rigor of our experimental demonstrations. We fully agree that the primary objective of these system-level demonstrations is to verify the mechanical and optical integrity of the FBGs post-assembly, as well as the effectiveness of the interfacial adhesion for accurate strain transfer. Therefore, in the revised manuscript, we have addressed these points by explicitly stating this verification purpose at the beginning of **Section 2.3** and providing further elaboration within the **Discussion section**. We have also clarified that while these FBG applications (vibration, speech, and gesture recognition) have been explored in earlier studies, they serve here as a rigorous validation of the structural and functional integrity of the DFP-integrated arrays. Furthermore, we have expanded our reference list to include several studies on FBG-based speech recognition and hand gesture monitoring to provide a more comprehensive academic context. The specific details of the revisions are as follows:

To evaluate the practical reliability and functional integrity of FBG arrays integrated via the DFP process, we conduct a series of system-level demonstrations under diverse

mechanical conditions. These experiments are designed not only to illustrate potential applications but, more importantly, to verify that the automated DFP assembly does not damage or degrade the optical and mechanical performance of the FBG sensors. While the use of FBGs for mechanical vibration, speech recognition, and hand gesture monitoring has been previously established [Van Meerbeek, et al. *Science Robotics* 3, eaau2489, doi:10.1126/scirobotics.aau2489; Liu, Y. et al. *Nano Research* 17, 7593-7602, doi:10.1007/s12274-024-6686-2; Tang, J. et al. *IEEE Sensors Journal* 26, 8188-8195, doi:10.1109/JSEN.2026.3656544.] these demonstrations are implemented here to confirm that the DFP paradigm preserves the sensors' full functionality. After assembly, the reflected spectra of the FBG arrays were measured to confirm stable and reliable optical responses, indicating that the programmed fiber routing does not introduce significant spectral distortion or signal loss. In addition, the following demonstrations in structural health monitoring and biomechanical sensing further confirm that the interfacial adhesion between the fiber and substrate is sufficiently robust to enable accurate strain transfer and reliable motion determination under both rigid and soft mechanical loading conditions. **(Please see the blue words on pages 7 and 8 of the revised manuscript).**

Critically, while bending typically compromises FBG performance through optical loss, our framework transforms these physical constraints into quantifiable design advantages. By establishing the $R_{3\min}$ threshold, the DFP paradigm bypasses traditional trial-and-error manual methods, maximizing integration density without compromising signal fidelity. Spectral characterization confirms that the automated routing induces negligible crosstalk or wavelength drift, proving the process preserves the structural and functional integrity of brittle sensors. The reliability of this approach is evidenced by high-fidelity strain transfer in structural displacement reconstruction (Fig. 3e) and superior classification accuracies in speech (95%) and gesture (90%) recognition. Ultimately, the DFP paradigm offers a scalable, programmatic route for integrating fragile fiber electronics onto complex, non-developable surfaces, overcoming the geometric constraints of conventional assembly and paving the way for fully automated

manufacturing of intelligent skins and soft robotics. **(Please see the blue words on pages 10 and 11 of the revised manuscript).**

Reviewer #3 (Remarks to the Author):

After reviewing the revised manuscript, I confirm that the authors have adequately addressed the concerns raised in my initial report. The revisions have clarified the scope of the work and improved the presentation of the proposed methodology.

In my opinion, the issues identified in the first review have been satisfactorily resolved.

Updated recommendation: Accept.

Response: We sincerely thank the reviewer for the positive recommendation and for the time and expertise devoted to evaluating our work. We are pleased that the revisions have clarified the scope and improved the presentation of our methodology. The reviewer's constructive feedback throughout the review process has been invaluable in improving the quality and scientific rigor of this manuscript. We gratefully acknowledge these helpful comments.

Reviewer #4 (Remarks to the Author):

This manuscript presents a programmable Direct-FBG-Patterning (DFP) assembly paradigm for the one-step, high-precision, conformal integration of pre-inscribed, multiplexed Fiber Bragg Grating (FBG) sensor arrays onto arbitrary planar and non-developable curved surfaces. The approach is mechanically inspired by Direct Ink Writing (DIW) in additive manufacturing: a custom-built five axis motion platform-comprising three translational axes (x, y, z) on the upper print needle and two rotational axes (V, W) on the lower substrate—continuously guides a single optical fiber containing multiple pre-inscribed FBGs along programmed trajectories, bonding it to an adhesive substrate layer. The key contribution is the development of a procedure that automates FBG placement. FO sensors have long been assembled manually, and the automated procedure here minimizes positioning error associated with the measurements. The framework is experimentally validated on three adhesive substrates

(optically clear adhesive (OCA), nanotape, and double-sided tape), with assembly speeds of 2–5 mm/s and a guiding needle of internal diameter 260 μm. The paper then demonstrates spatial cross-fiber and multilayer assembly strategies enabling highdensity, single-fiber multiplexed FBG sensor networks. Three system-level application demonstrations are presented:

1. Torsional displacement reconstruction in an aerospace-inspired wing-like structure using three FBG strain rosettes and the Modal Superposition Method (MSM);

2. Non-acoustic phonation monitoring on the laryngeal region with a 3 × 2 FBG array, a 3D convolutional recurrent neural network (3D-CRNN) with a dual-attention mechanism;

3. Epidermal hand-gesture recognition at the volar wrist using a CNN-GRU architecture.

The authors have successfully addressed all previous comments comprehensively.

The revised manuscript is notably cleaner in scope and framing. The responses to Reviewers 1 and 2 are thorough and scientifically honest, directly acknowledging the cross-disciplinary terminological ambiguity that caused the original confusion. The novelty lies in assembly science and integration engineering, not in grating physics or deep-learning architectures. This is now clearly articulated.

Response: We sincerely thank the reviewer for the careful evaluation of our manuscript and for the positive assessment of our work. We are particularly grateful for the reviewer's recognition that the core novelty of this study lies in the proposed assembly paradigm and integration engineering, and for acknowledging the refined scope and framing of the revised manuscript. The reviewer's professional insights and constructive feedback have been invaluable in strengthening the clarity and scientific rigor of our work. We appreciate the time and expertise you have dedicated to evaluating our research.

The following comments are minor points:

1. *Single-subject biological data: The phonation and gesture recognition experiments are performed on a single subject (implied by the protocol). Inter-subject variability is a*

well-known limitation in epidermal sensing; acknowledging this explicitly and flagging it as future work would improve scientific rigor.

Response: We sincerely thank the reviewer for this constructive comment regarding scientific rigor. We fully acknowledge that inter-subject variability, arising from differences in skin morphology, tissue stiffness, and individual motion patterns, is an important factor affecting the generalization of epidermal sensing systems. In this study, the biological demonstrations were primarily intended as a functional validation of the DFP assembly paradigm, demonstrating that the integrated sensor arrays can maintain their mechanical and optical integrity while capturing high-fidelity signals on complex and dynamic surfaces. As suggested, we have explicitly added a discussion of this limitation in the revised manuscript and highlighted the investigation of inter-subject variability as an important direction for future work. Specific details are as follows:

Beyond the specific demonstrations presented here, the generality of the DFP assembly paradigm suggests broad applicability to a wide range of fiber-based sensing systems and intelligent structures. The programmatic assembly of high-density sensor networks on complex, non-developable surfaces opens new opportunities in SHM, wearable electronics, soft robotics, and human-machine interaction, where conventional manual assembly imposes severe geometric and functional constraints. While these results underscore the technical versatility of the DFP paradigm, its application in human-centric sensing currently serves as a proof of concept. It should be noted that the biological data for phonation and gesture recognition were collected from a single subject. Since inter-subject variability remains a known challenge in epidermal electronics, future work will focus on multi-subject studies and the development of more robust machine learning models to account for physiological variations. Ultimately, by combining these refined algorithms with technical advancements such as adaptive path optimization and real-time feedback, we can achieve the fully automated assembly of smart sensor surfaces with unprecedented spatial resolution and functional complexity. **(Please see the blue words on page 12 of the revised manuscript).**

2. *Long-term adhesive stability: The DFP assembly relies entirely on pressure-sensitive adhesives, yet no data on adhesion durability over time (e.g., peel strength after days or weeks, or under repeated bending cycles) are presented. For the aerospace SHM demonstration in particular, durability is a critical practical concern that deserves at least a brief comment.*

Response: Thanks for the valuable comment regarding the long-term stability of the adhesive interface. Adhesion durability is indeed an important practical consideration, particularly for structural health monitoring applications. In response, we have added a brief discussion in the revised manuscript to emphasize the importance of long-term adhesion durability and to clarify that systematic investigations of long-term performance, including environmental aging and cyclic mechanical loading, will be an important direction for future work. The specific revisions are as follows:

In addition to environmental temperature considerations, the long-term stability of the adhesive interface is equally crucial for practical reliability. While the current demonstrations validate the robustness of DFP-integrated sensors under immediate loading, their resistance to environmental aging and mechanical fatigue under cyclic bending remains an essential avenue for future characterization. In aerospace applications, where bonding must maintain integrity over extended service lives, systematic durability testing will be essential. Future work will focus on quantifying the time-dependent peel strength and interfacial stability to ensure the performance of DFP-assembled networks in demanding field environments. **(Please see the blue words on pages 11 and 12 of the revised manuscript).**

3. *Temperature compensation in practice: While the authors correctly note that all experiments were conducted at a stable 26 ± 0.5 °C and that reference-grating-based compensation is compatible with the DFP layout, no experimental demonstration of temperature decoupling is provided. The Discussion acknowledgement is appropriate, but a back-of-envelope estimate of temperature-induced strain crosstalk in realistic operating conditions would be informative for readers targeting field deployment.*

Response: Thank you for your valuable comment. The reviewer highlights a critical aspect of practical field deployment. To provide the requested quantitative perspective, we have incorporated a "back-of-the-envelope" estimation in the revised Discussion section. This analysis evaluates temperature-induced strain crosstalk under realistic operating conditions and provides a clear benchmark for the errors that might arise without compensation. The specific response and revised text are provided below:

In this study, all experiments were conducted at a constant ambient temperature of approximately 26 °C. Under these stable thermal conditions, the $\Delta\lambda_T$ remains within a very small range and can be reasonably neglected, allowing the sensing response to be attributed primarily to mechanical strain. Despite this laboratory-scale simplification, temperature sensitivity remains an inherent factor for the practical deployment of FBG sensors. To provide a quantitative assessment for field applications, based on the typical temperature sensitivity of silica FBGs (10 pm/°C), a mere 1°C temperature fluctuation would introduce an apparent strain crosstalk of approximately 8.3 με [Bhaskar et al., *Results in Optics* 5, doi:10.1016/j.rio.2021.100130; Yassin et al., *Discover Civil Engineering* 1, doi:10.1007/s44290-024-00141-4.]. While this value is relatively minor compared to the high-amplitude strains recorded in our wing structural monitoring, it poses impact on capturing subtle physiological micro-vibrations. Consequently, in our current experimental validation for phonation and gesture recognition, maintaining a constant laboratory ambient temperature to eliminate thermal interference is essential for ensuring signal integrity and verifying process reliability. Furthermore, for complex application scenarios involving drastic thermal fluctuations, the DFP assembly paradigm exhibits excellent compatibility. Its high-precision programmable layout allows for the precise integration of reference gratings or the construction of differential measurement arrays within the same conformal layer, providing a robust hardware foundation for reliable signal decoupling. **(Please see the blue words on pages 18 and 19 of the revised Supplementary Material).**

4. *Throughput and scalability: The assembly speed (2–5 mm/s needle feed) is mentioned*

in the Methods but the total time required to pattern a complete sensing array is not reported. For readers considering practical adoption, a comparison of DFP assembly time against manual bonding would provide useful context.

Response: We sincerely thank the reviewer for this insightful suggestion. Providing the total assembly time indeed offers a more practical context for readers evaluating the DFP paradigm for real-world applications.

In response, we have selected representative patterns for integration experiments in the revised manuscript and systematically recorded the assembly times at various feed speeds. As illustrated in Fig. R1(a) and (b), we evaluated the total assembly times for two typical layouts: a serpentine structure on a flat substrate (total length of 211.4 mm) and an annular structure on a complex curved surface (total length of 350 mm), with feed speeds set at 2 mm/s and 5 mm/s, respectively. For the 211.4 mm serpentine structure on the flat substrate, the total assembly times were 41 s and 107 s, respectively; for the 350 mm annular structure on the curved surface, the assembly times were recorded as 76 s and 187 s. Concurrently, as shown in Fig. R1(c) a comparative analysis was performed between the measured assembly times and the theoretical predictions. The results demonstrate that while the experimental values align closely with the theoretical predictions under planar conditions, a slight deviation is observed under curved surface conditions. This discrepancy is primarily attributed to the structural compliance of our custom-built 5-axis platform and the kinematic synchronization constraints during multi-axis coordinated motion. Overall, these deviations remain within an acceptable range, suggesting that the total assembly time can be reasonably estimated by the ratio of the total path length to the corresponding feed speed. Consequently, the total assembly time of the system can be reliably estimated by the ratio of the total integrated path length to the corresponding feed speed, demonstrating robust linear scalability. Furthermore, regarding the comparison with manual assembly, no direct comparative experiments were conducted, as the high-precision patterned integration achieved by the DFP paradigm exceeds the physical limits of conventional manual positioning and manual bonding. This further highlight the unique advantages of the DFP process for high-precision and conformal

integration on complex curved surfaces. The relevant descriptions have been accordingly incorporated into the main text and Supplementary Information, as follows:

Under these parameters, the DFP paradigm demonstrates good assembly efficiency. Representative serpentine arrays (~ 211 mm) and annular paths on complex curved surfaces (~ 350 mm) are precisely integrated within 110 s and 190 s, respectively. Except for deviations resulting from structural compliance and kinematic synchronization constraints, the assembly time remains consistent with theoretical linear predictions (Section 4 of the Supplementary Material). Since the high precision achieved by DFP on non-developable surfaces fundamentally exceeds the physical limits of manual operation, manual deployment cannot serve as a performance benchmark, highlighting DFP's unique technical barriers in the digital manufacturing of complex, conformal sensing networks. **(Please see the blue words on pages 14 and 15 of the revised manuscript).**

4.3 Assembly efficiency analysis of the DFP paradigm

To establish a quantitative benchmark for process scalability, the total assembly time was systematically analyzed for diverse geometric trajectories. As illustrated in Fig. S12(a) and (b), two representative layouts were investigated: a planar serpentine trajectory (total length $L \approx 211$ mm) and a complex curved annular trajectory (total length $L \approx 350$ mm), with feed speeds (v) set at 2 mm/s and 5 mm/s, respectively. For the planar structure, the measured assembly times were 107 s and 41 s at feed speeds of 2 mm/s and 5 mm/s, respectively. For the curved structure, the corresponding assembly times were 187 s and 76 s. A comparison between the experimental results and theoretical predictions (L/v, Fig. S12(c)) shows good agreement under planar conditions. The slight deviations observed during curved surface integration are mainly attributed to the structural compliance of the custom-built five-axis platform and kinematic synchronization constraints during multi-axis coordinated interpolation. While minor discrepancies exist within acceptable limits, the assembly time remains highly predictable and scales linearly with path length and feed speed. Such consistency demonstrates the robust scalability of the DFP paradigm for large-area integrated

sensing networks. Furthermore, a direct comparison with manual assembly was not performed, as the high-precision patterned integration achieved here, particularly on non-developable surfaces, fundamentally exceeds the physical limitations of conventional manual positioning and bonding. This highlights the unique advantage of the DFP process in enabling high-fidelity conformal sensing networks that are unattainable through traditional manual approaches. **(Please see the blue words on pages 17 and 18 of the revised Supplementary Material).**

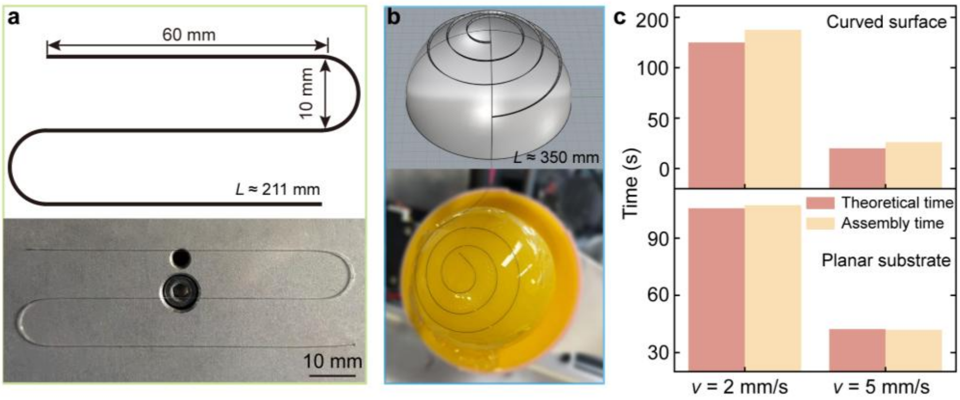

Fig. R1 Evaluation of assembly efficiency and scalability for the DFP paradigm. (a) Representative serpentine trajectory designed and fabricated on a planar substrate with a total path length L of approximately 211 mm. (b) Conformal annular trajectory designed and integrated on a hemispherical curved surface with an extended path length L of approximately 350 mm. (c) Quantitative comparison between the measured assembly times and theoretical predictions at feed speeds of 2 mm/s and 5 mm/s respectively.

The revised manuscript represents a scientifically sound and significant contribution to the field of FBG sensor integration. The authors have responded thoroughly to the first round of reviews, resolving the major conceptual confusion regarding terminology, adding substantive optical and mechanical characterization data, and substantially improving reproducibility documentation. The three-radius design framework is original and practically useful. The system-level demonstrations are impressive in scope, spanning rigid aerospace structures and compliant human tissue within a single unified assembly paradigm. The paper is well-written, clearly structured, and the

figures are of high quality.

Response: We are deeply grateful to the reviewer for the highly encouraging feedback and for recognizing the efforts made in our revision. We are particularly heartened that the clarifications on terminology, the additional data, and the refined documentation on reproducibility met the reviewer's expectations.

Reviewer #5 (Remarks to the Author):

I co-reviewed this manuscript with one of the reviewers who provided the listed reports. This is part of the Nature Communications initiative to facilitate training in peer review and to provide appropriate recognition for Early Career Researchers who co-review manuscripts.

Response: We would also like to express our sincere gratitude for your time and effort in participating in the *Nature Communications* co-review initiative. We appreciate the constructive insights you provided, which have been instrumental in enhancing the overall quality of this manuscript.

Response to reviewers' comments

We are grateful for the positive and encouraging feedback from the reviewers. We are pleased that our revisions have addressed the previous concerns and that the manuscript is now considered suitable for publication.

In response to the remaining minor suggestions and technical points, we have further refined the manuscript and supplementary material. For clarity, the reviewers' comments are presented in bold italics, followed by our responses in standard black font.

Thank you again for your constructive guidance throughout this process.

Reviewer #2 (Remarks to the Author):

The authors have made all revisions/changes suggested by the reviewers. The manuscript is suitable for publication.

Response: We sincerely thank the reviewer for their positive assessment and for recognizing the improvements made to our manuscript.

1. *I believe that there is a small typographical error on Page 7 of the supplementary material. The text appears as "The experimental data, representing bending loss per unit length versus the bend diameter, were plotted with error bars as shown in Fig. S2(c)." I think it should be Fig S4(b).*

Response: We sincerely thank the reviewer for their meticulous reading of our manuscript and for pointing out this error. We have corrected the reference in the Supplementary Information (Page 7) from Fig. S2(c) to Supplementary Fig. 4b.

2. *DOI Not Found*

   10.6084/m9.figshare.31770808

   This DOI cannot be found in the DOI System. Possible reasons are:

   The DOI is incorrect in your source. Search for the item by name, title, or other metadata using a search engine. The DOI was copied incorrectly. Check to see that the string includes all the characters before and after the slash and no sentence

punctuation marks. The DOI has not been activated yet. Please try again later, and report the problem if the error continues.

Response: We sincerely apologize for the initial technical delay in activating the DOI. We have now fully published the repository and confirmed that the DOI (https://doi.org/10.6084/m9.figshare.31817992) is fully active and functional.

Reviewer #4 (Remarks to the Author):

The authors have addressed all the comments from the reviewer and this manuscript now can be accepted for publication in its current form.

Response: We are very grateful to the reviewer for their positive recommendation and for the time they invested in evaluating our work. We are pleased that the current version has met the reviewer's standards for publication.

1. *the DOI did not work*

   Response: We sincerely apologize for the inconvenience caused by the technical issue with the DOI link. We have now officially published the dataset and activated the DOI (https://doi.org/10.6084/m9.figshare.31817992) in the Figshare database.

Reviewer #5 (Remarks to the Author):

I co-reviewed this manuscript with one of the reviewers who provided the listed reports. This is part of the Nature Communications initiative to facilitate training in peer review and to provide appropriate recognition for Early Career Researchers who co-review manuscripts.

Response: We sincerely appreciate your participation in the co-review of our manuscript. Thank you for the time and professional effort invested in evaluating our work.
